# Overexpression of a modified eIF4E regulates potato virus Y resistance at the transcriptional level in potato

**DOI:** 10.1186/s12864-019-6423-5

**Published:** 2020-01-06

**Authors:** Pablo A. Gutierrez Sanchez, Lavanya Babujee, Helena Jaramillo Mesa, Erica Arcibal, Megan Gannon, Dennis Halterman, Molly Jahn, Jiming Jiang, Aurélie M. Rakotondrafara

**Affiliations:** 1 Laboratorio de Microbiología Industrial, Facultad de Ciencias, Universidad Nacional de Colombia Sede Medellín, Calle 59 A N 63-20, Medellín, Colombia; 2 Department of Plant Pathology, University of Wisconsin-Madison, 1630 Linden Drive, Madison, WI 53706 USA; 3 U.S. Department of Agriculture-Agricultural Research Service, Madison, WI 53726 USA; 4 Department of Agronomy, University of Wisconsin-Madison, Moore Hall, 1575 Linden Drive, Madison, WI 53706 USA; 5 Department of Plant Biology, Department of Horticulture, Michigan State University, East Lansing, MI 48824 USA

**Keywords:** Potato virus Y, eIF4E, Recessive resistance, Potyviruses, Oxidative stress, Feedback regulation

## Abstract

**Background:**

Potato virus Y (PVY) is a major pathogen of potatoes with major impact on global agricultural production. Resistance to PVY can be achieved by engineering potatoes to express a recessive, resistant allele of eukaryotic translation initiation factor eIF4E, a host dependency factor essential to PVY replication. Here we analyzed transcriptome changes in eIF4E over-expressing potatoes to shed light on the mechanism underpinning eIF4E-mediated recessive PVY resistance.

**Results:**

As anticipated, modified eIF4E-expressing potatoes demonstrated a high level of resistance, eIF4E expression, and an unexpected suppression of the susceptible allele transcript, likely explaining the bulk of the potent antiviral phenotype. In resistant plants, we also detected marked upregulation of genes involved in cell stress responses.

**Conclusions:**

Our results reveal a previously unanticipated second layer of signaling attributable to eIF4E regulatory control, and potentially relevant to establishment of a broader, more systematic antiviral host defense.

## Background

Resistance to viruses can be conferred by disrupting key virus-host interfaces essential to viral replication [1]. In plants, there are several examples of recessive resistance wherein a recessive gene mutation for a specific viral host factor evolves, thereby preventing viral infection or genome replication through loss-of-function [2–4]. This defense strategy contrasts with dominant resistance wherein pathogens are detected based on avirulence determinants, termed ‘effectors’ [5]. Upon interception of the effector, recognition results in active inhibition of viral replication and movement by triggering cell death response, thus confining the virus to the site of entry [6].

While recessive resistance can, in theory, be attributed to mutations in any gene essential to viral replication, recessive viral resistance genes often encode translation initiation factors [4, 7]. A prominent example in plants is the eukaryotic translation initiation factor 4E (eIF4E) and its isoform eIFiso4E, variants of which can represent potent loss-of-susceptibility determinants affecting many viruses, in particular members of the *Potyviridae* family. In both plants and animals, eIF4E is the small subunit and the cap-binding protein in the eIF4F complex, which is also comprised of an RNA helicase (eIF4A) and a large scaffold factor (eIF4G) [8]. The recruitment of the ribosomal subunit to the 5′ end of the mRNA is directed by eIF4E, which is bound to the 5′ m7GpppG-cap of the mRNA. In plants, eIF4E and eIF4G are also present as eIFiso4E and eIFiso4G isoforms that share similar functions in translation [9, 10]. Another member of the eIF4E multigene family is the novel cap binding protein (nCBP) or 4EHP, which is distantly related to eIF4E and eIFiso4E with a weaker cap-binding function [11].

Allelic variants of plant eIF4E and eIFiso4E that confer virus resistance typically differ from susceptible alleles due to their limited number of amino acid substitutions that cluster near the cap-binding pocket [7, 12, 13]. Importantly, these variants have no discernible effect on plant viability despite their potent antiviral activities [14]. For potyviruses, antiviral eIF4E variants disrupt the ability of the virus to recruit ribosomes to the VPg protein linked to the 5′ end of the viral (+) strand genome [2, 3]. These alleles are found in nature [7] but can also be engineered directly into crops of importance or particular high susceptibility, using modern CRISPR/Cas9, ethyl methanesulfonate- or transposon-mediated mutagenesis, or inhibitory RNA (RNAi) strategies, [15–17]. The nature of the eIF4E/eIFiso4E mutations and genetic backgrounds of plants can affect the efficacy and the spectrum of the resistance [14, 18, 19]. Analysis of eIF4E-engineered loss-of-function plants revealed the feedback regulation between members of the eIF4E multigene family, at least at a post-translational level [14], that may hamper broad-spectrum effectiveness of the deployed resistance [18, 19].

The potyvirus *Potato virus Y* (PVY) is the most important viral pathogen of potatoes and the most common source of seed lot rejection in North America [20]. The spread of PVY can cause tuber yield reductions of up to 80% depending on variety and time of incubation [21, 22]. PVY^O^ is the most frequently found strain in circulation, with one of the major challenges to agriculture being detection and control of new PVY recombinants including PVY^N:O^ and PVY^NTN^ [23–26]. We and other groups have demonstrated various degrees of resistance to PVY for otherwise highly susceptible commercial potato cultivars after transgenic ectopic expression of eIF4E alleles [27–29]. Constitutive expression of potato4E:*pvr*1^2^, a modified Russet Burbank potato eIF4E that contained three mutations (I70N, L82R and D112N) similar to the amino acid substitutions in the natural PVY-resistance *pvr*1^2^ allele in *Capsicum annuum,* protected tetraploid Russet Burbank, Russet Norkotah, and Atlantic potato cultivars from PVY^O^, PVY^N:O^ and PVY^NTN^ infection [27, 28, 30]. No virus was found in the inoculated leaves, newly emerged leaves, or sprouted tubers in most of the transgenic potato lines, in spite of the susceptible genetic background of the potato cultivars. Crosses between the transformed and the parental lines demonstrated that the engineered resistance gene can be inherited in a dominant manner [28]. Intriguingly, not all combinations of amino acid substitutions from naturally occurring eIF4E alleles found in PVY-resistant pepper and tomato transferred resistance in potatoes [27], suggesting the existence of additional species-specific pathogenicity determinants. Consistent with this notion, Russet Burbank potatoes over-expressing *Eva1*, a natural variant of eIF4E-1 allele from *S. chacoense* that bears a 10-amino acid substitution predicted to fully disrupt the crucial eIF4E-VPg interaction, only showed a delay in symptom development and remained susceptible to PVY infection unless the endogenous susceptible eIF4E allele was simultaneously suppressed [29].

The above observations demonstrate that the mechanism(s) of recessive resistance conferred by modified *eIF4E* alleles require(s) a better understanding before attempting to deploy these genes into new cultivars. It remains to be investigated to which extent the ratio of the modified versus native alleles, the nature of the sequence substitutions, and/or the regulatory effect within the eIF4E gene family, contribute in the efficacy of the synthetic eIF4E-mediated resistance. The core hypothesis underpinning eIF4E antiviral activity in the context of recessive resistance has been that the transgene be expressed at levels much higher than the endogenous protein, thus monopolizing the translation machinery [31]. Here, we directly test this hypothesis by subjecting wild-type and potato4E:*pvr*1^2^ transgenic Atlantic potato lines [28] to global transcriptome analysis using Illumina TruSeq. Our results confirm that eIF4E-engineered resistance to PVY correlates with high levels of potato4E:*pvr*1^2^ expression but also reveal that potato4E:*pvr*1^2^ expression correlates with a potent suppression of the endogenous, susceptible *eIF4E* allele, at the transcriptional or post-transcriptional level. Moreover, we uncover that potato4E:*pvr*1^2^ overexpression induces deregulation of some genes encoding cell stress response factors, suggesting both a previously unanticipated possible role for eIF4E as gene regulator in plants, as reported in animals [32, 33], and possibly revealing a supplementary layer of indirect, systemic resistance relevant to the potency of the antiviral phenotype.

## Results

### Over-expression of potato4E:pvr1^2^ represses the transcription of native eIF4E mRNAs

**Fig. 1:**
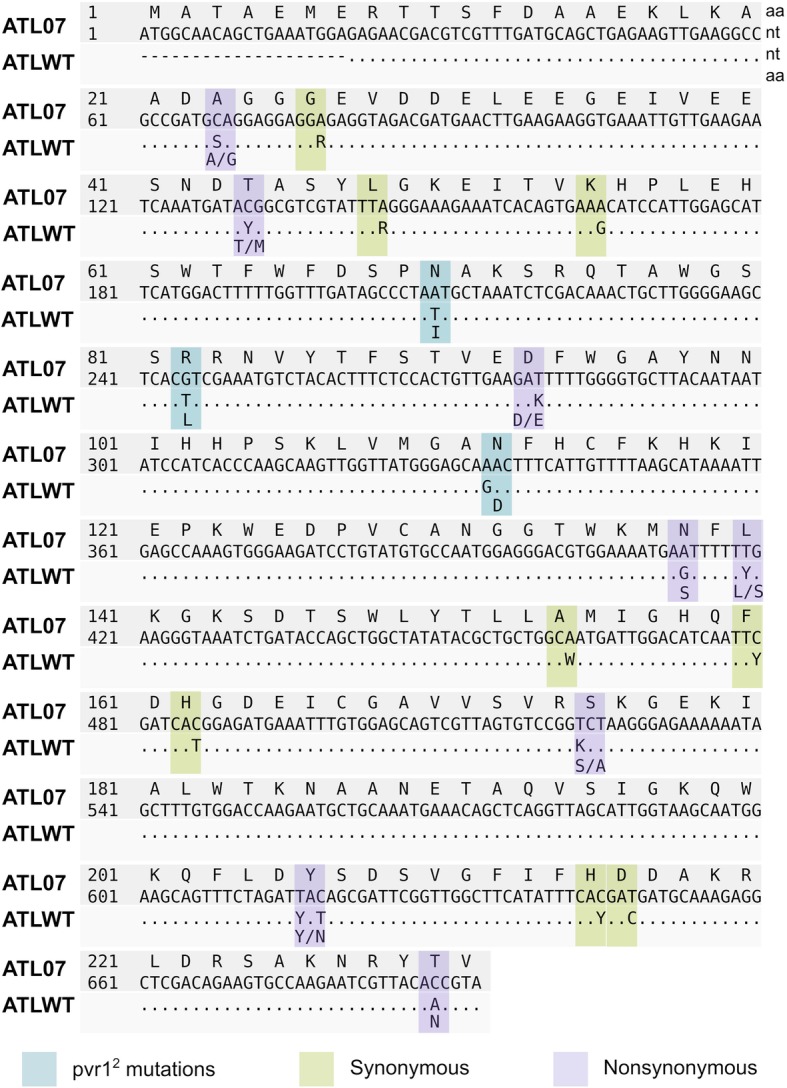
Sequence alignment of the eIF4E gene family in modified ATL07 and non-transformed ATLWT tetraploid Atlantic potatoes. The first two lines represent the consensus eIF4E amino acid sequence and its corresponding nucleotide coding sequence as obtained from the ATL07 RNAseq data. The third line highlights sequence similarities (dots) and differences found with the ATLWT dataset. Polymorphic sites are represented using IUPAC nucleotide ambiguity codes. Changes in the predicted amino acid sequence of the eIF4E protein from ATLWT are shown in the fourth line. Sequence changes representing the pepper PVY-resistance *pvr*1^2^ eIF4E allele mutations, synonymous and non-synonymous substitutions are highlighted in blue, yellow, and purple, respectively. The specific nucleotide sequences of the eIF4E multigene family are found in Additional file 2: Figure S2

**Fig. 2:**
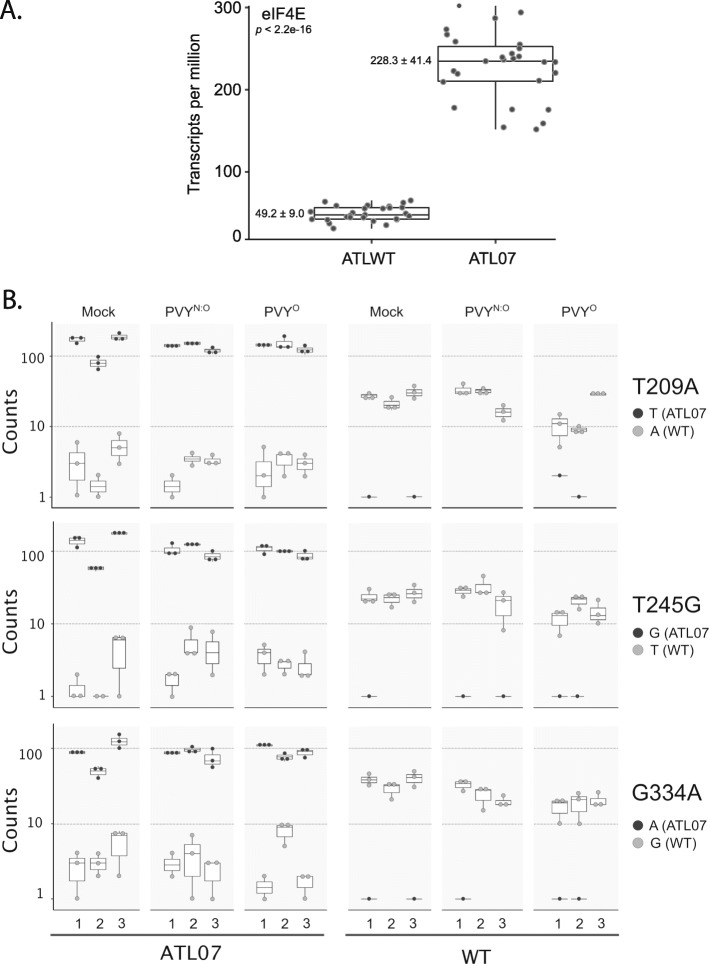
Abundance of native and endogenous eIF4E allele transcripts in the ATLWT and ATL07 datasets. All samples presented in this study are shown in the boxplot. Transcript abundance was measured as transcripts per million (TPM). Average values and standard deviation for each dataset are shown to the left of each box. The central horizontal lines in each box represent the median while the bottom and top lines represent the first and third quartile, respectively. **a** Overall abundance of the eIF4E transcript levels in ATLWT and ATL07 plants. Each point represents the TPM value for each treatment (Mock, PVY^O^, and PVY^N:O^). The levels of eIF4E were significantly different in both treatments (*p* > 2.2 e-16). **b** comparison of the abundance of potato4E:*pvr*1^2^ transcripts bearing the T209A (top), T245G (middle), and G334A (bottom) mutations, or native (WT) eIF4E transcripts, in transgenic ATL07 (left) or ATLWT (right) plants following mock inoculation or inoculation with PVY^O^ or PVY^N:O^

**Fig. 3:**
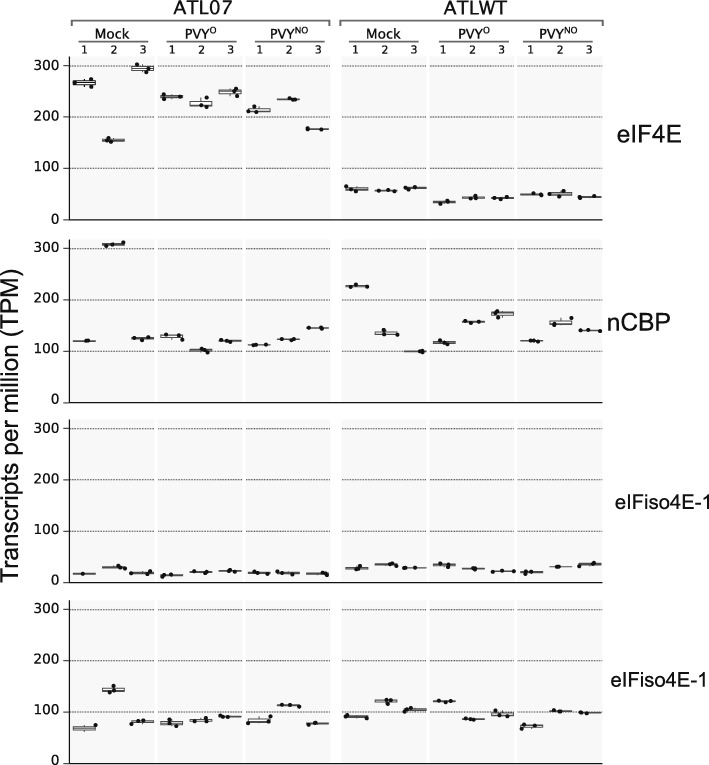
Comparison of transcription levels between eIF4E homologs in the ATL07 and ATLWT plants. Each panel represents the transcript levels of translation initiation factor eIF4E, novel cap-binding protein (nCBP), and the two eIFiso4E alleles in the modified ATL07 plants and in the susceptible ATLWT plants. Horizontal lines in each box represent the median (center), first (bottom) and third (top) quartiles of the TPM values. Each boxplot corresponds to three technical repeats for each biological treatment repetition in mock- and PVY^O^/PVY^N:O^-inoculated plants. For the TPM counts, the eIF4E homologs were mapped to the *S. tuberosum* reference sequences available at NCBI with accession codes NM_001288408 (eIFiso4E-1), NM_001288204 (eIFiso4E-2), and NM_006351298 (nCBP)

We previously described transgenic Atlantic and Russet Norkotah potato lines that were transformed to express potato4E:*pvr*1^2^ and exhibited varying degrees of resistance to a variety of PVY strains [27, 28]. Due to the limited number of nucleotide polymorphisms (base pairs 209, 245, and 334) between the transgene and the endogenous *eIF4E* alleles, we were not able to differentiate expression of each allele using real-time RT-qPCR. Hence, to gain further insight on the factors that regulate the efficacy of the eIF4E-mediated resistance and to study the impact of potato4E:*pvr*1^2^ expression on the host transcriptome, we compared one of the transgenic Atlantic cultivars, ATL07, that showed low copy of potato4E:*pvr*1^2^ insertion (Additional file 1: Figure S1) and an inheritable resistance phenotype against PVY [28], to the parental non-transformed line (ATLWT) using next-generation RNA sequencing (Illumina TruSeq). For each plant, we generated ~ 1 billion reads for three biological replicates (three experimental repeats each); with reads per library ranging from 14 to 20 million (Additional file 3: Table S1). We first identified the different *eIF4E* gene family members in ATLWT and ATL07 RNA datasets by comparing them to the *S. tuberosum* potato eIF4E NCBI reference sequence (NM_001288431) that shows a single *eIF4E* gene located on chromosome 3, a single eIFiso4E gene located on chromosome 9, and a single novel cap binding protein (nCBP) gene located on chromosome 10. For the Atlantic cultivar, we also identified a single *nCBP* allele but detected two eIF4E alleles (*eIF4Ea* and *eIF4Eb*), with the most abundant eIF4E variant representing about 72.2 ± 11.3% of the total eIF4E transcripts based on the polymorphic sites (Table 1), and two eIFiso4E alleles (Fig. 1 and Additional file 2: Figure S2). This reveals that the tetraploid cultivar Atlantic is heterozygous for both eIF4E and eIFiso4E, and homozygous for nCBP. For the ATL07 line, we confirmed that the Russet Burbank potato4E:*pvr*1^2^ transgene differed from the native *eIF4E* homologs by detecting the anticipated three *pvr*1^2^ mutations at nucleotides T209A, G245T, and A334G, and also at six homozygous and 11 heterozygous nucleotide positions, characteristic of the Russet Burbank *eIF4E* allele backbone (Fig. 1 and Table 1). In line with constitutive expression of potato4E:*pvr*1^2^, a significant increase (4.6-fold, *P*-value < 2.2e-16) in overall *eIF4E* expression was observed for ATL07 plants relative to ATLWT plants, with an average of 228.3 ± 41.4 transcripts per million (TPM) in ATL07 to contrast to the 49.2 ± 9.0 TPM in ATLWT (Fig. 2a and Additional file 4: Table S2). Based on the total nucleotide counts at the polymorphic sites (Table 1), 94.9 ± 3.1% of the total ATL07 eIF4E transcripts corresponded to the potato4E:*pvr*1^2^ gene. Compared to ATLWT plants, the expression of native *eIF4E* alleles, normalized to the average values of reads at the mutated sites, was severely reduced in all ATL07 plants assayed, down to 13–15% of that in the ATLWT plants (Table 2 and Additional file 5: Table S3), representing 4.8% of the total *eIF4E* transcripts in all ATL07 plants. In contrast, expression of the other *eIF4E* paralogs, including *eIFiso4E* and the *nCBP*, was largely indistinguishable between ATLWT and ATL07 plants (Fig. 3). Accordingly, the potato4E:*pvr*1^2^ transgene not only outcompeted the native *eIF4E* locus in ATL07 plants for net gene expression but also, somehow, was able to suppress native eIF4E transcript abundance.

**Table 1:** Sequence coverage at variable nucleotide positions between eIF4E sequences in the ATL07 and ATLWT plants. Sequence coverages of the eIF4E *pvr*1^2^ mutations (T209A, G245T, A334G) are represented in bold. Raw depth corresponds to the total nucleotide count at each position. A1 represents the most frequent nucleotide observed at that position and A2 the second most abundant. The relative abundance of each nucleotide is shown in parentheses

Position	ATL07					ATLWT				
	Raw Depth	A1	Depth (%)	A2	Depth (%)	Raw Depth	A1	Depth (%)	A2	Depth (%)
68	978	C	976 (99.8)	G	1 (0.1)	130	C	107 (82.3)	G	22 (16.9)
78	987	A	973 (98.6)	G	13 (1.3)	168	A	128 (76.2)	G	40 (23.8)
131	2233	C	2207 (98.8)	T	20 (0.9)	602	C	454 (75.4)	T	146 (24.3)
144	1530	A	1503 (98.2)	G	27 (1.8)	521	G	270 (51.8)	A	251 (48.2)
165	182	A	163 (89.6)	G	18 (9.9)	301	G	300 (99.7)	–	
**209**	**1538**	**A**	**1464 (95.2)**	**T**	**71 (4.6)**	**611**	**T**	**611 (100)**	**–**	
**245**	**2924**	**G**	**2847 (97.4)**	**T**	**72 (2.5)**	**590**	**T**	**587 (99.5)**	**–**	
279	3009	T	2946 (97.9)	G	60 (2.0)	646	G	394 (61.0)	T	250 (38.7)
**334**	**2240**	**A**	**2144 (95.7)**	**G**	**90 (4.0)**	**702**	**G**	**695 (99.0)**	**–**	
413	7614	A	7359 (96.6)	G	245 (3.2)	1487	G	1469 (98.8)	–	
419	7464	T	7357 (98.6)	C	98 (1.3)	1436	T	886 (61.7)	C	550 (38.3)
462	494	A	466 (94.3)	T	27 (5.5)	427	T	372 (87.1)	A	54 (12.6)
480	2302	C	2250 (97.7)	T	48 (2.1)	797	T	566 (71.0)	C	231 (29.0)
486	7114	C	6972 (98.0)	T	137 (1.9)	1489	T	1252 (84.1)	C	233 (15.6)
523	8630	T	8559 (99.2)	G	59 (0.7)	1376	T	1035 (75.2)	G	338 (24.6)
616	316	T	294 (93.0)	C	21 (6.6)	370	C	220 (59.5)	T	149 (40.3)
618	316	C	281 (88.9)	T	35 (11.1)	381	T	359 (94.2)	–	
645	1210	C	1172 (96.9)	T	37 (3.1)	351	T	286 (81.5)	C	64 (18.2)
648	1440	T	1386 (96.2)	C	51 (3.5)	337	C	331 (98.2)	–	
690	3415	C	3398 (99.5)	A	17 (0.5)	64	A	61 (95.3)	–

**Table 2:** Average values of reads per million (RPM) for the three *Pvr*1^2^ mutations at nucleotides A209T, G245T, and A334G in the eIF4E assembly for ATL07 and ATLWT data sets

	Position 209				Position 245				Position 334			
	ATL07		WT		ATL07		WT		ATL07		WT	
	A	T	A	T	G	T	G	T	A	G	A	G
Average (RPM)	7.9	0.2	0	1.3	6.1	0.2	0	1.3	5	0.2	0	1.5

### Resistance against PVY correlated with extremely low level of viral RNAs

**Fig. 4:**
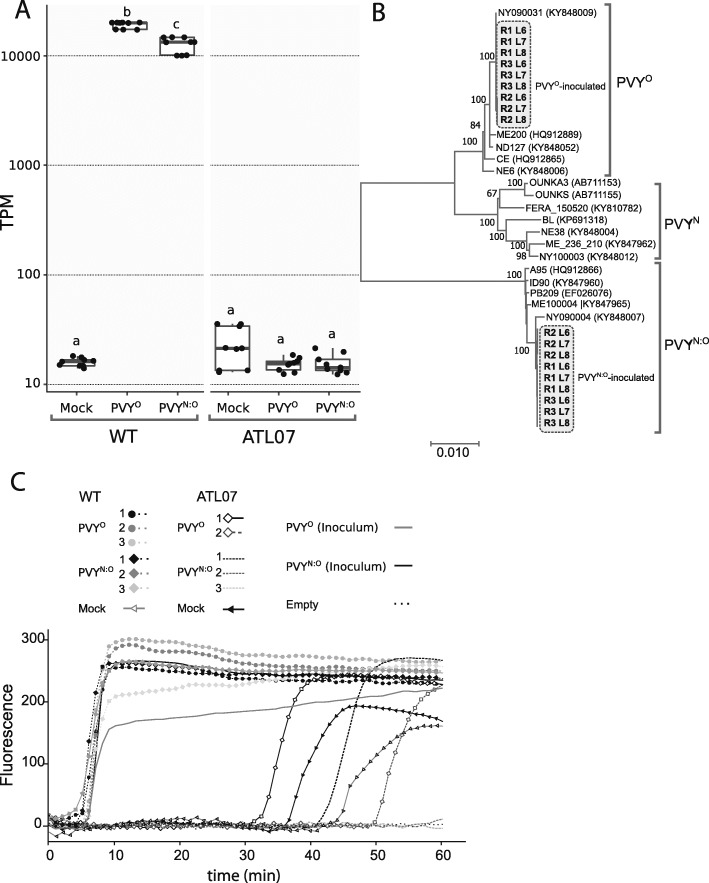
Potato virus Y levels in the ATL07 and ATLWT plants. **a** Boxplot showing the transcript per million (TPM) of PVY^O^ and PVY^N:O^ with respect to the *S. tuberosum* reference transcriptome in the ATL07 and ATLWT plants following mock- and/or PVY-inoculation. Each box is represented by three repetitions with three technical replicates each. Letters represent groups that showed significant mean TPM differences using Tukey’s Honestly Significant Difference (HSD) Test (*P*-value < 0.001). **b** Neighbor-Joining tree showing the phylogenetic affinity of the PVY assemblies from the PVY-inoculated WT plants. PVY genomes were assembled with NCBI Magic-BLAST RNAseq mapping tool using the reference PVY genome (NC_001616) as mapping template. Assemblies and consensus sequences were analyzed using IGV [34]. **c** Comparison of the amplification speeds in the RT-LAMP assay for PVY coat protein detection from total RNA isolated from ATL07 and ATLWT plants following mock- or PVY-inoculation. We used no template as a negative control. As a positive control, we included total RNA from PVY^O^ and PVY^N:O^ inoculum sources

To study PVY-host interactions in these plants, we first analyzed changes in the level of expression of eIF4E upon viral infection. PVY infection had negligible effect in the ATL07 plants on the overall transcript ratio of the eIF4E transgene versus native allele, with the level of the endogenous *eIF4E* transcripts remaining at relatively low level as in the mock-treated plants (Fig. 2b and Additional file 5: Table S3), and had also no impact on the expression of the other eIF4E gene families (Fig. 3). We next quantified levels of host and viral RNAs 21 days post inoculation in the ATLWT and ATL07 plants challenged with the PVY^O^ and necrotic recombinant PVY^N:O^ strains. We measured viral RNA levels by de novo assembly of the PVY genomes using the reference PVY genome (NC_001616) as a mapping template. The abundance of PVY reads revealed that 1.8% of total reads mapping to the PVY genome from the infected ATLWT plants (Additional file 6: Table S4). The assembly of the PVY genome in the inoculated WT plants validated that each tested plant was infected with the intended viral strains (Fig. 4a and b). As anticipated, only background levels of PVY^O^ and PVY^N:O^ RNAs were detected in ATL07 plants relative to ATLWT, confirming particularly strong resistance to PVY replication potential (Fig. 4a). The susceptible ATLWT plants showed TPM values of 12,705 and 19,133 for PVY^N:O^ and PVY^O^ (*P* value <1e-10), respectively. This represented about a 400- to 600-fold increase when compared to those in the transformed ATL07 plants, with TPM values of 15.6 for PVY^N:O^ and 15.3 for PVY^O^, which was similar to that of all mock-inoculated control plants (average of 16.5 TPM), which we considered as background level (Fig. 4a and Additional file 6: Table S4). We obtained similar results using isothermal reverse transcriptase loop-mediated amplification (RT-LAMP) for the detection of the viral coat protein in inoculated and non-inoculated leaf tissues (Fig. 4c).

Taken together, these data demonstrate that over-expression of the *pvr*1^2^–like *eIF4E* allele establishes strong resistance to two independent PVY strains. Resistance could map to either the abundance of modified eIF4E, which the virus cannot utilize; to the relative paucity of endogenous, susceptible *eIF4E* gene expression, which the virus requires; or a combination of both potato4E:*pvr*1^2^ effects. On a related note, the data also suggest that PVY must be unable to utilize the other eIF4E variants in the presence of potato4E:*pvr*1^2^, while their levels remained similar in both ATL07 and ATLWT lines, at least at the RNA transcript level.

### Marked global changes to gene expression in response to potato4E: *pvr*1^2^ and PVY infection

**Fig. 5:**
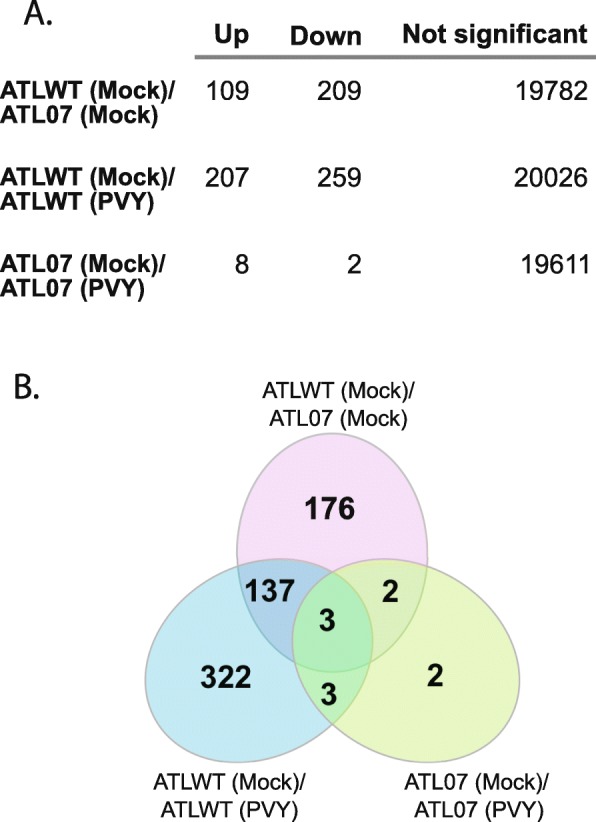
Differential expression analysis of transcripts between ATL07 and ATLWT mock- or PVY-inoculation. **a** Total number of upregulated and downregulated transcripts with at least a 2-fold change between mock-inoculated ATLWT and ATL07 plants, mock- and PVY-inoculated ATLWT, and mock- and PVY-inoculated ATL07 plants. **b** Venn diagram representing the total number of differentially expressed genes shared between each comparison

**Fig. 6:**
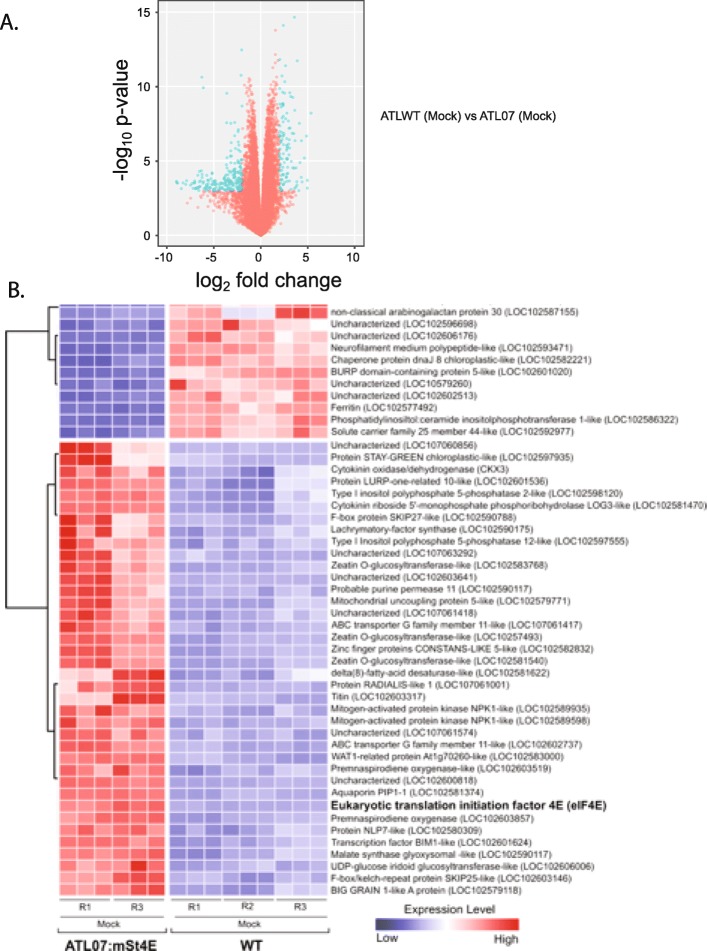
Differential expression analysis of transcripts between mock-inoculated ATL07 and ATLWT plants. **a** Volcano plot showing the differentially expressed genes (in blue) with at least a 2-fold change between mock-inoculated ATLWT and ATL07 plants. **b** The correlation heatmap diagram of the top 50 most differentially expressed (DE) genes between the mock-inoculated ATLWT and ATL07 plants, revealing genes with expression correlated to the presence of potato4E:*pvr*1^2^. Each column represents a single technical replicate. Biological repetitions for each treatment are labelled from R1 to R3. A hierarchical cluster of genes with similar expression patterns is shown to the left. Expression levels are colored from dark blue to red to represent high and low levels, respectively. Gene annotations and locus names are shown to the right

That endogenous eIF4E transcript accumulation was suppressed in the ATL07 lines prompted us to next investigate the global effects of potato4E: *pvr*1^2^ overexpression on the plant transcriptome. Differentially expressed genes (DEG) in ATLWT vs. ATL07 strains were determined by changes in TPM calculated using a combination of log2FC and *P*-value criteria, mapping individual reads against the potato genome as a reference (Figs. 5 and 6). Overall, 318 genes were differentially expressed with at least a 2-fold change in expression in the ATL07 plants relative to those in ATLWT (Figs. 5 and 6a). Of these, 109 genes were upregulated and 209 genes were downregulated (Fig. 5 and Additional file 7: Table S5). Illustrated in the heatmap in Fig. 6 were the 50 most DEGs whose expressions were strongly correlated to the over-expression of eIF4E, revealing a potential eIF4E-regulon (Fig. 6b). Gene Ontology (GO) enrichment analysis yielded 138 unique GO functional annotation terms, with 90 in the biological process category and the rest within the cellular component (11) and molecular function categories (37). Intra-group analysis of the biological process category revealed that reactive oxygen processes and responses to stresses were the major enriched GO terms (summarized in Table 3). The categories included stress response (GO:0006950), response to stimuli (GO:0050896), genes related to response to reactive oxygen species (GO:0000302), response to oxygen-containing compound (GO:1901700), response to hydrogen peroxide (GO:0042542), response to oxidative stress (GO:0006979), and response to various abiotic stimulus (GO:0009628), heat (GO:0009408) and temperature (GO:0009266). Combined, this analysis suggested that potato4E: *pvr*1^2^ overexpression could potentially deregulate the expression of genes involved in sensing, signaling or controlling levels of oxidative species, and in buffering against specific stress conditions (Fig. 6b and Table 3).

**Table 3:** List of the differentially expressed genes in mock-ATL07 within the most enriched GO terms, which were related to oxidative pathways and stress responses. Highlighted as A are the genes that were differentially expressed in the mock-treated ATL07 plants, AB are the genes that were differentially expressed in both ATL07 mock and ATLWT PVY-inoculated plants

Related to oxidative pathway							
A: ATL07mock B:ATLWT PVY	Response	Go Term	Category	Description	GeneID	ProteinID	Annotation
AB	Down/Down	GO:0006979	P	response to oxidative stress	102,578,720	XP_015170961.1	L-ascorbate_peroxidase_cytosolic(LOC102578720)
AB	Down/Down	GO:0006979	P	response to oxidative stress	102,594,742	XP_015162428.1	uncharacterized (LOC102594742)
AB	Down/Down	GO:0006979	P	response to oxidative stress	102,596,945	XP_006341175.1	uncharacterized (LOC102596945)
AB	Down/Down	GO:0042542	P	response to hydrogen peroxide	102,578,969	XP_006346594.1	26.5_kDa_heat_shock_protein_mitochondrial(LOC102578969)
AB	Down/Down	GO:0042542	P	response to hydrogen peroxide	102,583,475	XP_015170470.1	heat_shock_70_kDa_protein_8(LOC102583475)
AB	Down/Down	GO:0042542	P	response to hydrogen peroxide	102,591,190	XP_006342905.1	17.4_kDa_class_III_heat_shock_protein(LOC102591190)
AB	Down/Down	GO:0000302	P	response to reactive oxygen species	102,594,103	XP_006346757.1	15.7_kDa_heat_shock_protein_peroxisomal(LOC102594103)
AB	Down/Down	GO:0000304	P	response to singlet oxygen	102,599,238	NP_001305587.1	(EE)-geranyllinalool_synthase(LOC102599238)
A	Up	GO:0016491	F	oxidoreductase activity	102,581,622	XP_006345577.1	delta(8)-fatty-acid_desaturase-like (LOC102581622)
A	Up	GO:0016491	F	oxidoreductase activity	102,581,792	XP_006357900.1	gibberellin_20_oxidase_1(LOC102581792)
A	Up	GO:0016491	F	oxidoreductase activity	102,581,872	XP_006350059.1	protein_ECERIFERUM_3-like (LOC102581872)
A	Down	GO:0016705	F	oxidoreductase activity	102,597,820	XP_006363763.1	cytochrome_P450_CYP72A219-like (LOC102597820)
A	Down	GO:0016705	F	oxidoreductase activity	102,604,056	XP_006367342.1	cytochrome_P450_83B1-like (LOC102604056)
A	Up	GO:0055114	F	oxidative -reduction process	102,592,722	XP_006358835.1	cytochrome_b561_and_DOMON_domain-containing_protein_At3g25290-like (LOC102592722)
AB	Up/Up	GO:0016709	F	oxidoreductase activity	102,598,017	XP_006362126.1	cytochrome_P450_78A6-like (LOC102598017)
A	Down	GO:00055114	F	oxidative -reduction process	102,579,798	XP_006358956.1	homogentisate_12-dioxygenase (LOC102579798)
A	Up	GO:0016705	F	oxidoreductase activity	102,603,519	XP_006350395.1	premnaspirodiene_oxygenase-like (LOC102603519)
A	Up	GO:0016705	F	oxidoreductase activity	102,603,857	NP_001305614.1	premnaspirodiene_oxygenase(LOC102603857)
A	Up	GO:0016705	F	oxidoreductase activity	102,577,568	NP_001275219.1	CYP86A33_fatty_acid_omega-hydroxylase (LOC102577568)
Related to stress response							
AB	Down/Down	GO:0006950	P	response to stress	102,596,667	XP_015166769.1	low-temperature-induced_65_kDa_protein-like (LOC102596667)
AB	Down/Down	GO:0006950	P	response to stress	102,597,686	XP_015160032.1	late_embryogenesis_abundant_protein-like (LOC102597686)
AB	Down/Down	GO:0006950	P	response to stress	102,602,565	XP_006361910.1	low-temperature-induced_78_kDa_protein-like (LOC102602565)
AB	Down/Down	GO:0006950	P	response to stress	107,057,685	XP_015160026.1	abscisic_acid_and_environmental_stress-inducible_protein_TAS14-like (LOC107057685)
AB	Down/Down	GO:0009611	P	response to wounding	102,599,238	NP_001305587.1	(EE)-geranyllinalool_synthase(LOC102599238)
AB	Down/Down	GO:0009408	P	response to heat	102,578,969	XP_006346594.1	26.5_kDa_heat_shock_protein_mitochondrial(LOC102578969)
AB	Down/Down	GO:0009408	P	response to heat	102,583,475	XP_015170470.1	heat_shock_70_kDa_protein_8(LOC102583475)
AB	Down/Down	GO:0009408	P	response to heat	102,584,371	XP_006345019.1	22.7_kDa_class_IV_heat_shock_protein-like (LOC102584371)
AB	Down/Down	GO:0009408	P	response to heat	102,587,639	XP_006338640.1	18.1_kDa_class_I_heat_shock_protein-like (LOC102587639)
AB	Down/Down	GO:0009408	P	response to heat	102,589,078	XP_015170489.1	17.4_kDa_class_I_heat_shock_protein-like (LOC102589078)
AB	Down/Down	GO:0009408	P	response to heat	102,589,396	XP_006360822.1	17.4_kDa_class_I_heat_shock_protein-like (LOC102589396)
AB	Down/Down	GO:0009408	P	response to heat	102,591,190	XP_006342905.1	17.4_kDa_class_III_heat_shock_protein(LOC102591190)
AB	Down/Down	GO:0009408	P	response to heat	102,594,103	XP_006346757.1	15.7_kDa_heat_shock_protein_peroxisomal(LOC102594103)
AB	Down/Down	GO:0009408	P	response to heat	102,601,494	XP_006349271.1	22.7_kDa_class_IV_heat_shock_protein-like (LOC102601494)
A	Down	GO:0009416	P	response to light stimulus	102,600,485	XP_015164872.1	protein_LIGHT-DEPENDENT_SHORT_HYPOCOTYLS_10-like (LOC102600485)
AB	Down/Down	GO:0009644	P	response to high light intensity	102,583,475	XP_015170470.1	heat_shock_70_kDa_protein_8(LOC102583475)
AB	Down/Down	GO:0043617	P	cellular response to sucrose starvation	102,577,576	XP_006344055.1	asparagine_synthetase_[glutamine-hydrolyzing](LOC102577576)

**Fig. 7:**
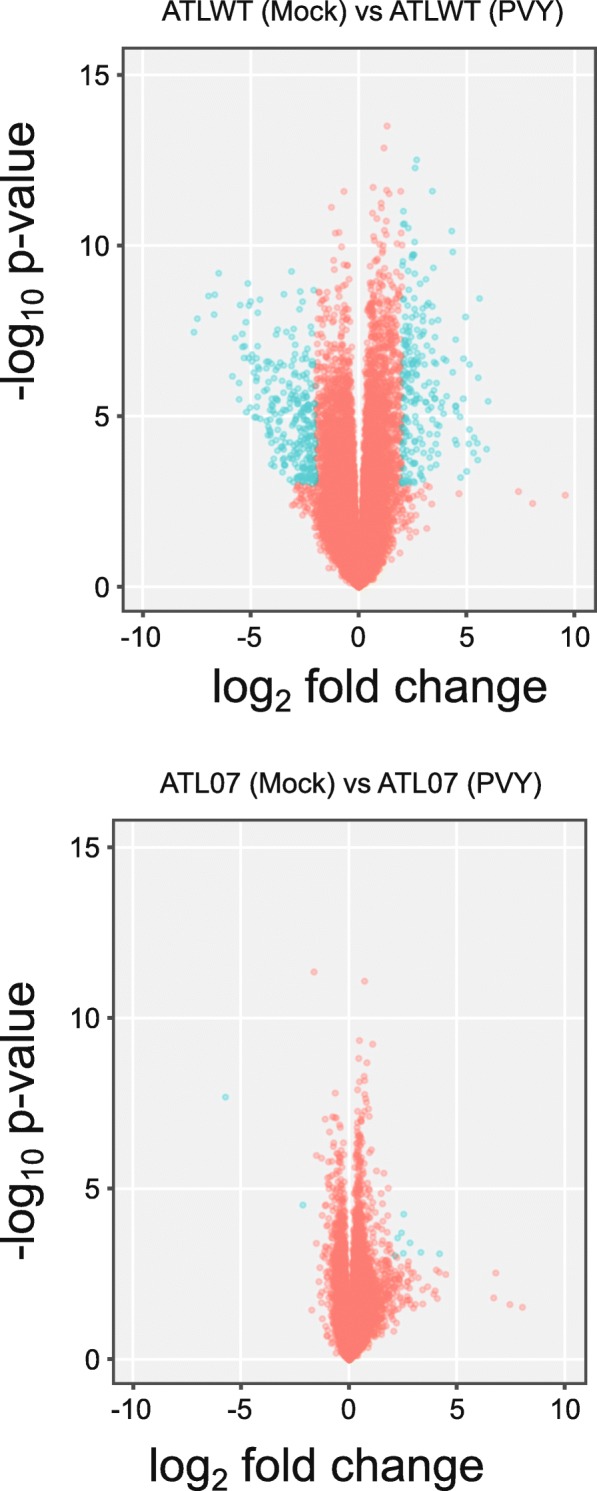
Volcano plot comparing the differentially expressed genes upon PVY inoculation of the ATLWT and ATL07 plants. The plot highlights the difference in the transcriptional changes between the ATL07 and ATLWT plants in response to PVY infection, consistent with the ATL07 plants’ resistance to PVY infection

**Fig. 8:**
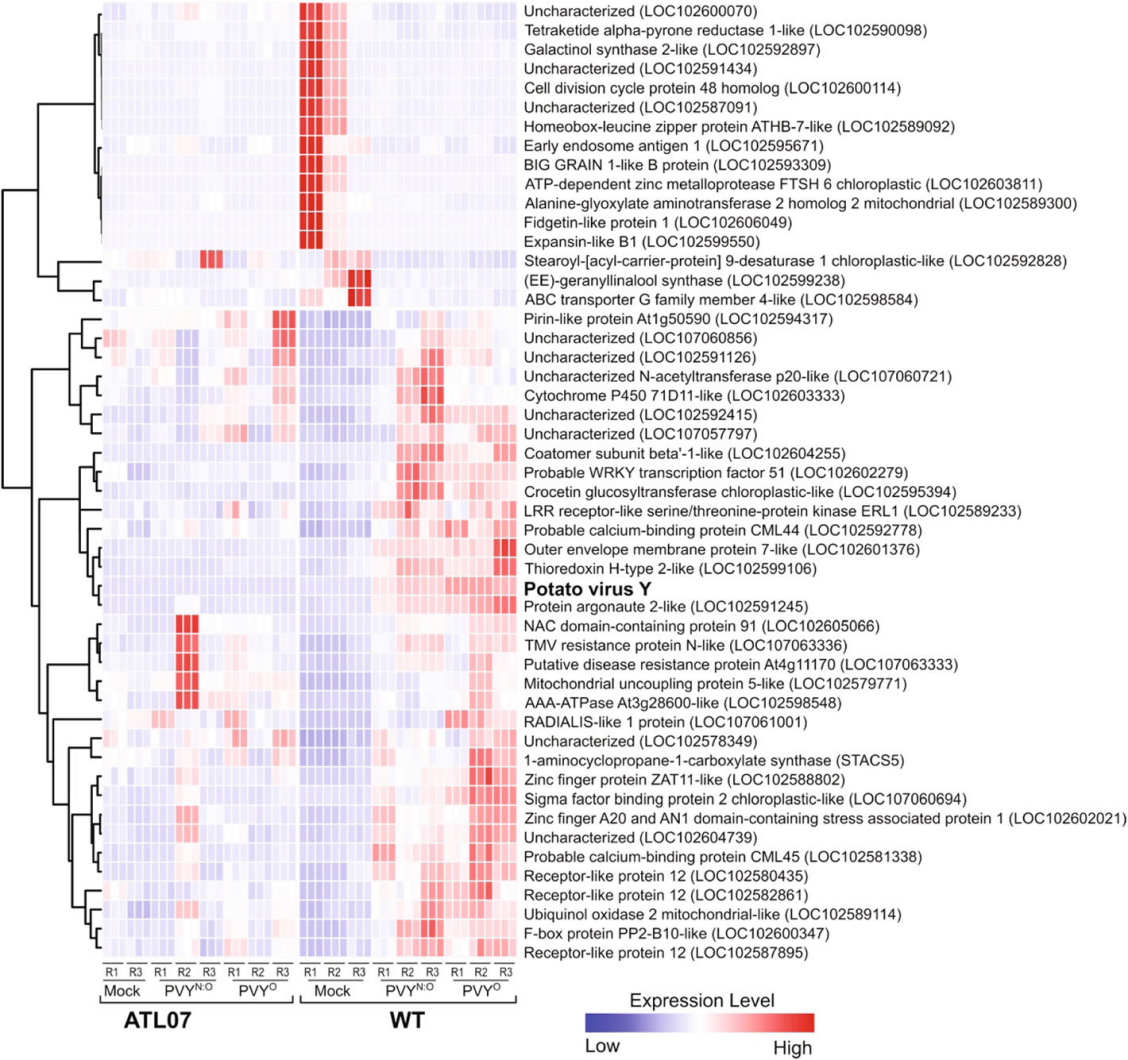
Correlation heatmap diagram of the 50 top most differentially expressed genes in the ATLWT-mock vs ATLWT-PVY comparison. The diagram illustrates the wide re-programming of the ATLWT plants in response to virus infection. Expression levels of the corresponding genes in the resistant ATL07 plants were included for comparison. Each column represents a single technical replicate. Biological repetitions for each treatment are labelled from R1 to R3. A hierarchical cluster of genes with similar expression patterns is shown to the left. Expression levels are colored from dark blue to red to represent high and low levels, respectively. Gene annotations and locus names are shown to the right

**Fig. 9:**
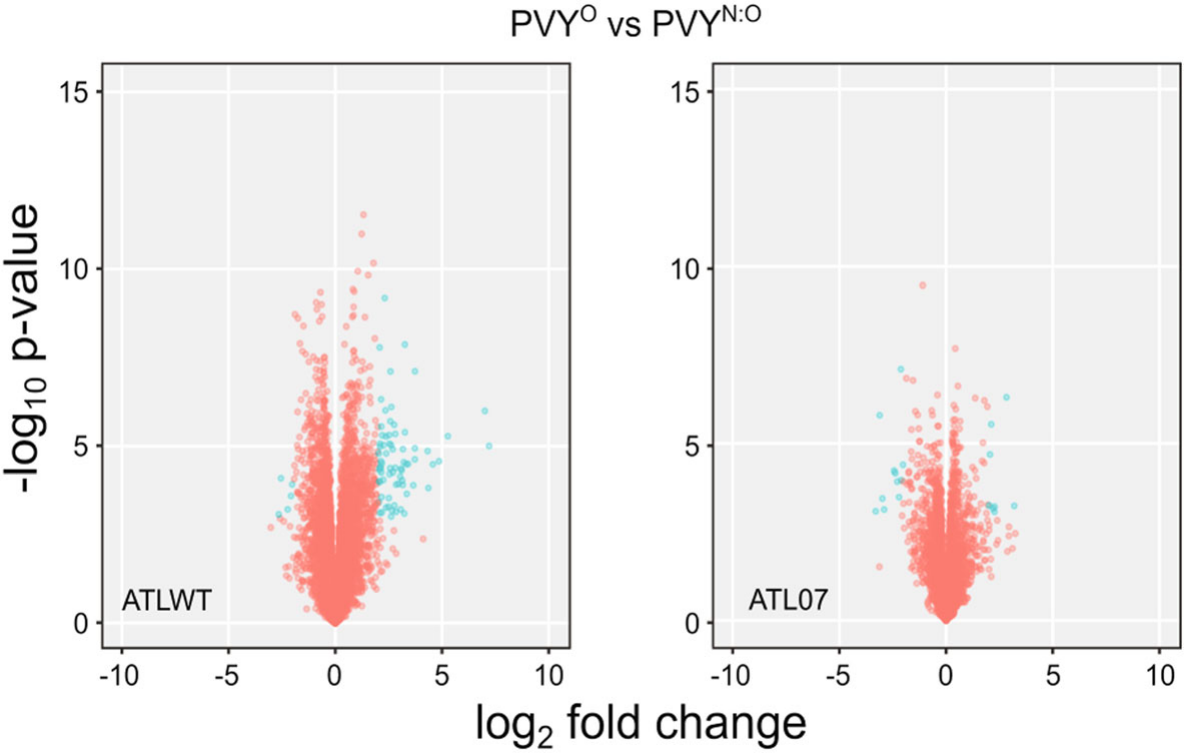
Comparison of differentially expressed genes between PVY^O^ and PVY^N:O^ in the ATL07 and ATLWT plants. Volcano plot showing the differentially expressed genes (in blue) with at least a 2-fold change when comparing PVY^O^ and PVY^N:O^ inoculated ATLWT and ATL07 plants

We next compared the global transcriptome changes upon PVY infection of the ATLWT and ATL07 lines to that of the mock-inoculated transgenic plants at 21 days post-infection (Fig. 7). As anticipated, PVY infection significantly altered the abundance of 466 transcripts in the ATLWT plants, in line with a broader range re-programming of the host transcriptome (Figs. 7 and 8). Intra-group analysis of the biological process category revealed that 60% of the top DEGs in this category (38 genes) were linked to oxidative reduction processes, similar to changes observed with potato4E: *pvr*1^2^ overexpression (Additional file 8: Table S6 and Additional file 5: Table S3, see “AB” labelled genes that corresponded to the genes that were differentially expressed in both ATL07 mock and ATLWT PVY-inoculated plants). The second top category was cell wall synthesis and related processes (35 genes), in line with the reports of cytological and histological changes that occur upon PVY infection [35, 36] (Additional file 7: Table S5). The top GO categories in the molecular function group were linked to DNA binding and transcription factor activity (55 genes), with many genes involved in phytohormone and ethylene responses [37] (Additional file 8: Table S6). Notably, out of the 466 transcripts that were differentially expressed upon PVY infection, 152 genes responded differentially to PVY^O^ and to PVY^N:O^ infection (Figs. 8 and 9).

To contrast, we observed few transcriptional changes in the transformed ATL07 plants in response to PVY. Of a total 19,621 transcripts detected, only 10 genes (based on a 2-fold cut-off change) were differentially regulated in response to PVY infection, regardless of the strain type (Fig. 7). These included 5 heat-shock related genes and two DNA-binding factors (all upregulated, see Table 3).

A comparison of the transcriptome data between the mock-inoculated ATL07 and the PVY infected ATLWT plants revealed that 44% of the DEGs (140 out of 318 genes) associated with potato4E:pvr1^2^ over-expression were also differentially expressed in ATLWT upon viral infection (Fig. 5 and Table 3). In particular, 25 out of the top 36 DEGs for both conditions were associated with the oxidative pathway and stress responses (Table 3, labelled as “AB”). Accordingly, an alternative explanation for the antiviral effects of potato4E: *pvr*1^2^ against PVY infection may be its capacity to yield constitutive, systemic host antiviral immune signaling.

## Discussion

The eIF4E gene has emerged as a major factor governing host susceptibility to positive sense RNA viruses. Resistance to infection can sometimes be conferred by expressing structural variants of eIF4E postulated to impede the stage of viral translation initiation [7, 12, 13]. eIF4E-conferred resistance to members of the *Potyviridae* family, which includes PVY, has been widely reported for both monocot and dicot plants [4, 38]. Herein we exploited next-generation sequencing to study the mechanism conferring PVY resistance in transgenic Atlantic potato cultivar modified to express high levels of the potato4E:pvr1^2^ resistance allele.

Our analysis revealed three key observations. First, we found that increased expression of potato4E:pvr1^2^, which differs from the susceptible eIF4E allele by three point mutations, caused marked down-regulation of endogenous susceptible *eIF4Ea* and *eIF4Eb* gene expression while having little to no effect on other eIF4E isoforms or nCBP. We have yet to discern the mechanism involved, and test it in the other established transgenic lines. While it remains to be tested whether the decrease in these transcripts resulted from RNA silencing, we postulate that potato4E:pvr1^2^ overexpression may be capable of triggering an concentration-dependent auto-regulatory feedback loop, similar to a mechanism previously suggested for *Arabidopsis thaliana* wherein knockout of the *At–eIFiso4E* triggered marked increases of At–eIF4E1 protein synthesis [14]. Our result shows a specific down-regulation of the native eIF4E transcripts at either a transcriptional or post-transcriptional level. Duan et al. (2012) [29] previously showed that expression of *Eva1*, a natural variant of potato *eIF4E* that carries a 10-amino acid substitution predicted to disrupt the eIF4E -VPg interaction, was insufficient to confer PVY resistance without additional depletion of native eIF4E expression. Accordingly, it is reasonable to hypothesize that downregulation of native eIF4E in ATL07 plants contributes also to the efficacy of the resistance phenotype, even if the mechanism remains yet to be determined. This is in line with the observation that the resistance phenotype resulting from *eIF4E* gene knock-out approaches can confer a broader spectrum of resistance in other crops against different potyviruses [18, 19].

Second, because eIFiso4E and nCBP levels are unchanged in cells expressing potato4E:pvr1^2^, in the context of native eIF4E depletion, it seems unlikely that either of these eIF4E orthologs play a role in PVY infection, at least in potatoes.

Third, a surprising aspect of this analysis was that while the overexpression of potato4E:pvr1^2^ was designed to physically disrupt virus-eIF4E interaction, our transcriptome analysis uncovered a potential set of the eIF4E-regulons that could possibly be contributing to the resistance phenotype. Moreover, many of the same genes involved in cell stress responses were also found to be deregulated in PVY-infected plants. In animals, beyond a role in translation, eIF4E has been shown to regulate a subset of genes involved in key stress responses, including the detoxification of reactive oxygen species (ROS) for normal cellular function and control of oxidative stress [32, 33]. This function is particularly important in cancer and tumor development, which is often associated with a major increase in eIF4E levels to protect the cells from ROS accumulation [32]. Oxidative activity in plants is proposed to be required for recognition and processing of stress factors, and is part of a protective mechanism against pathogens to trigger cell death [33, 39]. Considering the strong association of oxidative and cell stress pathways in the context of natural plant defenses against viruses, it is compelling to consider that potato4E:pvr1^2^ overexpression could actually be operating indirectly, at least in part, to suppress PVY infection through triggering host stress responses.

## Conclusions

Based on the results of our comparative transcriptome analysis we propose that the failure of PVY to infect ATL07 plants results from the combinatory effect of, at least, (1) the abundance of the eIF4E resistant allele, which the virus cannot recruit; (2) the inability of PVY to access the product of the native susceptible allele, whose expression is repressed, and (3), plausibly, the capacity of potato4E:pvr1^2^ overexpression to upregulate expression of additional antiviral pathways. Further studies of these resistant plant species are warranted considering the potential relevance of these mechanisms to broad-spectrum control of positive sense RNA viruses that cause profoundly impact agricultural production.

## Methods

### Plant material and PVY strains

All plant materials used in this study consisted of the potato cultivar Atlantic. Multiple-node in vitro plants were obtained from the Potato Tissue Culture Laboratory (Wisconsin Seed Potato Certification program, University of Wisconsin-Madison). The untransformed plants and the ATL07 eIF4E-transgenic-potato Atlantic line that was previously characterized [28] were clonally propagated by planting stem cuttings taken from different regions of tissue-cultured mother plantlets. The cuttings were planted in a greenhouse for 2 weeks to promote rooting.

### PVY inoculation

Three clonally propagated plants were used for each mock and viral inoculation treatments 4 weeks after the cuttings were planted. Each plant corresponded to one biological repeat. They were inoculated either with different Potato virus Y strains or with water (control) on two consecutive days, as described previously [28]. Frozen PVY^O^ (isolate NY090031) and PVY^NTN^ (isolate NY090004) maintained in tobacco leaves were used as source of viral inoculum. Twenty-one days post inoculation, newly emerged systemic leaves were harvested for total RNA extraction.

### DNA blot

DNA blotting was performed to assess copy number variation of the transgene in the transformed ATL07 line, using non-transformed Atlantic wild type (ATLWT) as a negative control. Total genomic DNA from ATL07 and susceptible ATLWT was extracted using the modified CTAB method [40]. Next, 10 μg of purified genomic DNA was digested with *EcoRI* restriction enzyme, separated on a 1% agarose gel, and blotted on a nylon membrane overnight (Hybond-N+, GE Healthcare Life Sciences). *EcoRI* is expected to cleave once within the neomycin phosphotransferase II (*NPTII*) gene region positioned upstream of the eIF4E transgene. The probe, which corresponds to the *NPTII* gene (795 bp) [41], was PCR amplified from the Potato4E:pvr1^2^ cDNA clone using the primer set (Forward-TGGCTATATACGCTGCTGGC; Reverse-CGGGAGCGGCGATACCGTAAAGC) and 5′ end-labeled with ^32^P dCTPs by use of the Prime-It® RmT Random Primer Labeling Kit. Following hybridization, the gel blot was visualized using a phospho-imager.

### RNA extraction

Total RNA was extracted from leaves of each of the three treated and control plants using the RNeasy Plant mini kit (Qiagen) according to the manufacturer’s directions, with a total of 9 samples. An additional DNase I treatment (Ambion) was included to remove contaminating genomic DNA. Quality of the RNA was assessed by agarose gel electrophoresis.

### RNA-seq

One microgram of total RNA from each of the three biological repeats from both the inoculated and mock-treated samples was sent to the Biotechnology facility at Michigan State University for sequencing library preparation and RNA sequencing. Libraries were prepared using the Illumina TruSeq Stranded mRNA Library Preparation Kit on a Perkin Elmer Sciclone G3 robot following manufacturer’s recommendations. Completed libraries that passed quality control were quantified using a combination of Qubit dsDNA HS and Caliper LabChipGX HS DNA assays. Based on this quantitation, all 18 libraries were pooled in equimolar amounts for multiplexed sequencing. This pool was quantified using the Kapa Biosystems Illumina Library Quantification qPCR kit and then loaded on three lanes of an Illumina HiSeq 4000 flow cell and sequenced in a 1 × 50 bp single read format using HiSeq 4000 SBS reagents. Base calling was done by Illumina Real Time Analysis (RTA) v2.7.6 and output of RTA was demultiplexed and converted to FastQ format with Illumina Bcl2fastq v2.19.0.

### RT-LAMP assay

The RT-LAMP was performed as previously described [42] using the Y4 primer set, which targeted the PVY coat protein. The reaction mixture (10 μl) contained 0.375 μM each of outer primer (F3, B3), 1.5 μM each of inner primer (FIP, BIP), 0.75 μM each of loop primer (LB), 1X Isothermal Master Mix containing proprietary fluorescent dye (Novazym Polska sc.), 0.25 U AMV reverse transcriptase (Superscript III, Invitrogen) with 100 pg of total RNA as a template. The thermal profile in a CFX96 Touch™ Real-Time PCR Detection System (BioRad Ltd) included 60 cycles of 30 s at 65 °C.

#### Read mapping

A database comprising all reference RNA sequences from *S. tuberosum* available at NCBI plus the PVY^N:O^ and PVY^O^ consensus sequences obtained from the data were used to map the reads using the program RSEM [43]. The dataset comprised a total of 43,173 sequences: 2 viruses; 37,676 mRNAs; 315 partial mRNAs; 215 microRNAs; 3344 ncRNAs and 1641 other RNAs. Reference sequences were prepared using Bowtie2 [44]. Expression levels were quantified per locus, a table mapping all sequence isoforms to genes is provided in Additional file 5: Table S3. Nucleotide variants were identified using VCFtools [45].

#### Differential expression analysis

Differential expression analysis was performed with edgeR [46] using the expected count data obtained with RSEM. Only genes with at least two counts per million (CPM) in at least three samples were used in the analysis. A trimmed mean of M values (TMM) normalization was performed to correct for composition biases between the libraries [47]. Significance of differential expression for each gene was determined with the QL F-test, a gene was considered Differential expressed if *P*-value < 0.01 and abs (Log2FC) > 2. Analyses of variance and Multiple comparison of means using Tukey’s honestly significant difference tests were performed using RStudio (http://www.rstudio.com/). It is worth noting that one of the clonally propagated ATL07 repeats (R2) showed a lower expression level of all eIF4E transcripts compared to repeat 1 (R1) and repeat 3 (R3), with a TPM of 154.9 ± 3.6. This represented a significant deviation with respect to R1 (*P* < 0.001) and R3 (P < 0.001). Therefore, our subsequent analyses, unless noted, were focused on R1 and R3 repeats, which included a combined total of more than 113 million reads (Additional file 3: Table S1).

### Gene ontology annotation and analysis

A database comprising all reference proteins from *S. tuberosum* at NCBI, consisting of 38,055 entries, was used for the analysis (File: Stuberosum_proteins.fasta). GO terms were transferred from a database comprising all GO annotated proteins (3,305,440 proteins) from angiosperms available at Uniprot using Blastp and customs perl scripts written for that purpose. The correspondence between NCBI protein accession codes and the Angiosperm database can be found in the file: Stuberosum_NCBI_vs_uniprot_Angiosperms.txt. A total of 37,647 *S. tuberosum* proteins were annotated with GO terms. A GO annotation file (GAF) containing annotations made to the GO can be found in the file S_tuberosum_GAF.txt. Enrichment analysis for GO terms was performed with the topGO package [48] using Fisher’s exact test. The gene universe consisted all protein coding genes detected in the RNAseq experiments which corresponds to a total of 19,658 GO annotated genes. The file containing all the GO annotations can be found in: Additional file 7: Table S5 and Additional file 8: Table S6.

#### Phylogenetic analysis

Evolutionary distances were computed using the Tamura-Nei method [49] and are in the units of the number of base substitutions per site. The tree was calculated in MEGA7 using 1000 bootstrap replicates [50]. PVY genomes were assembled with NCBI Magic-BLAST using the reference PVY genome (NC_001616) as mapping template. Assemblies and consensus sequences were analyzed using IGV [34].

## Supplementary information

**Additional file 1: Figure S1.** Southern blot of untransformed ATLWT and transformed ATL07 lines probing for the neomycin phosphotransferase II (NPTII) gene. **a.** Diagram of the gene cassette used in transformation and the NPTII region targeted using a 794 nt probe for transgenic verification by Southern Blot probe (gray arrows). **b.** The southern blot with lane 1: 1kb ladder, lane 2: Atlantic non-transformed control (ATLWT), lane 3: Atlantic transgenic plant (ALT07).

**Additional file 2: Figure S2.** Specific nucleotide sequences of the eIF4E multigene family found in Atlantic potato cultivars including eIF4E homologs (eIF4E a and eIF4E b), and the eIF4E isoforms.

**Additional file 3 : Table S1.** Total number of reads obtained in each RNA-seq run

**Additional file 4 : Table S2.** Abundance of eIF4E sequences in each sample in Transcripts per million (TPM) and Reads per Kilobase per Million (RPKM, in parentheses).

**Additional file 5 : Table S3.** Table of counts for each nucleotide at the potato4E:pvr12 sites in the eIF4E assembly for each data set.

**Additional file 6 : Table S4.** Abundance of PVY sequences in each sample in Transcripts per million (TPM) and Reads per Kilobase per Million (RPKM, in parentheses).

**Additional file 7 : Table S5.** Differentially expressed genes in various comparisons

**Additional file 8 : Table S6.** GO enrichment analysis on gene sets in various treatments

## Data Availability

All data analyzed during this study are included in this published article and its additional information files. The raw data are available from the corresponding author on reasonable request.

## Abbreviations

ATL: Atlantic cultivar
eIF4E: Eukaryotic translation initiation factor 4E
nCPB: Novel cap binding protein
PVY: Potato virus Y
ROS: Reactive oxygen species
