# Supplementary figures and images for: Overexpression of a modified eIF4E regulates potato virus Y resistance at the transcriptional level in potato
Source: BMC Genomics. 2020 Jan 6;21:18. doi: 10.1186/s12864-019-6423-5 (PMC6945410; doi:10.1186/s12864-019-6423-5)

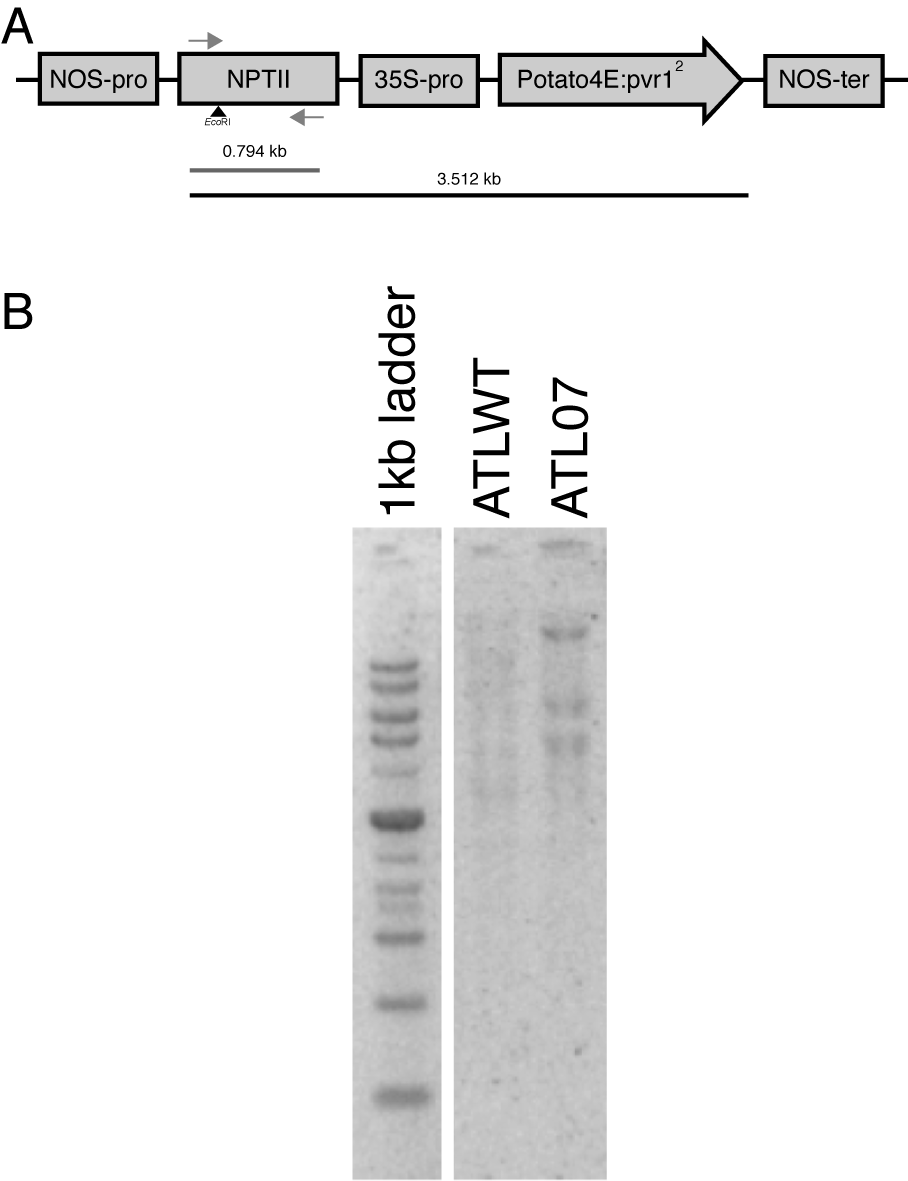

Supplement: Supplementary file 1 — Additional file 1: Figure S1. Southern blot of untransformed ATLWT and transformed ATL07 lines probing for the neomycin phosphotransferase II (NPTII) gene. a. Diagram of the gene cassette used in transformation and the NPTII region targeted using a 794 nt probe for transgenic verification by Southern Blot probe (gray arrows). b. The southern blot with lane 1: 1kb ladder, lane 2: Atlantic non-transformed control (ATLWT), lane 3: Atlantic transgenic plant (ALT07). [file 12864_2019_6423_MOESM1_ESM.tif]

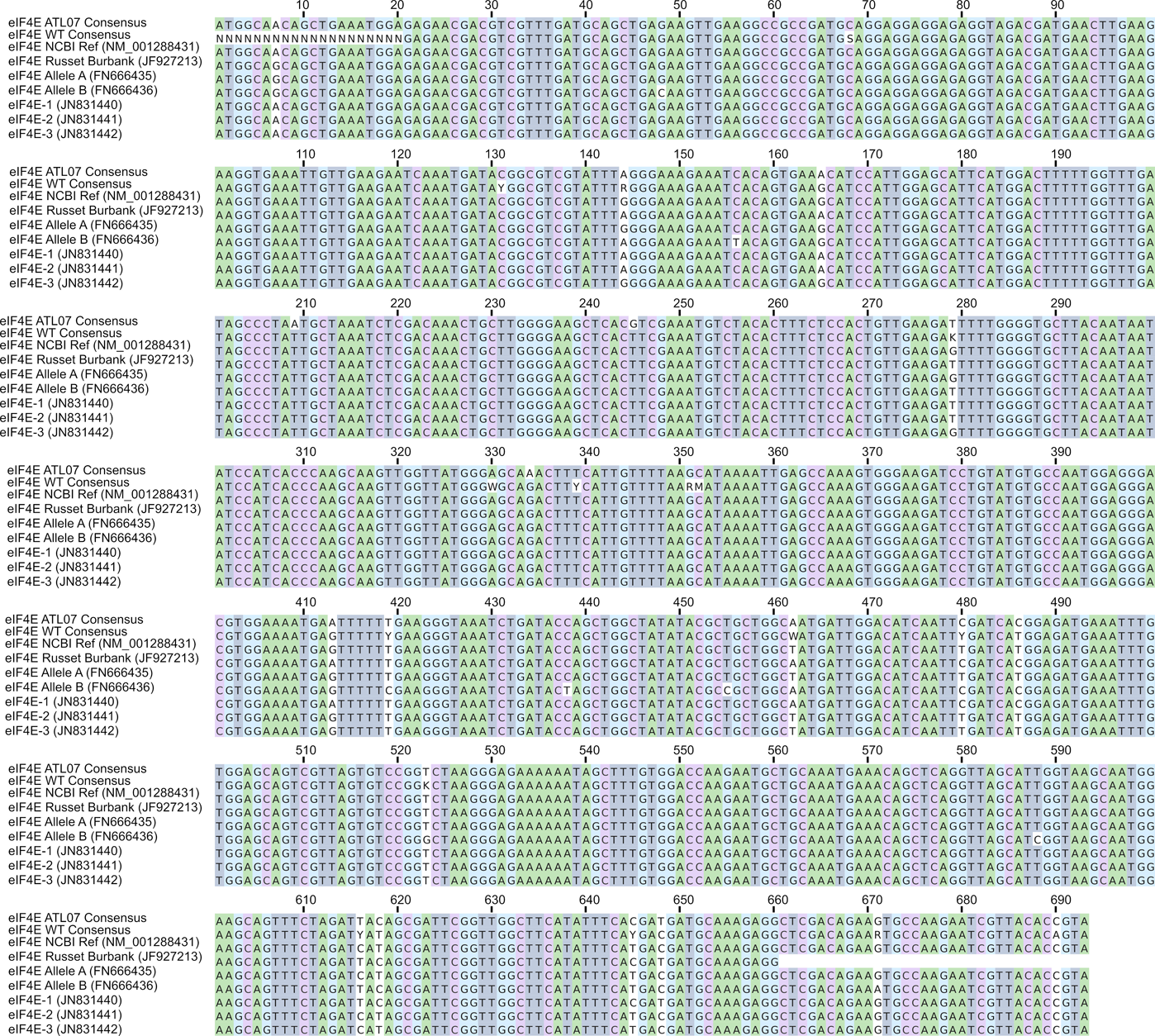

Supplement: Supplementary file 2 — Additional file 2: Figure S2. Specific nucleotide sequences of the eIF4E multigene family found in Atlantic potato cultivars including eIF4E homologs (eIF4E a and eIF4E b), and the eIF4E isoforms. [file 12864_2019_6423_MOESM2_ESM.tif]
