# Supplementary material for: Overexpression of a modified eIF4E regulates potato virus Y resistance at the transcriptional level in potato
Source: BMC Genomics. 2020 Jan 6;21:18. doi: 10.1186/s12864-019-6423-5 (PMC6945410; doi:10.1186/s12864-019-6423-5)
Supplement: Supplementary file 3 — Additional file 3 : Table S1. Total number of reads obtained in each RNA-seq run [file 12864_2019_6423_MOESM3_ESM.docx]

**Additional Table 1.** Total number of reads obtained in each RNA-seq run

|  |  |  | Mock |  | PVY^N:O^ |  | PVY^O^ |
| --- | --- | --- | --- | --- | --- | --- | --- |
|  |  |  |  |  |  |  |  |
| ATL07 | R1 | L6 | 19,323,900 |  | 18,573,888 |  | 15,450,644 |
|  |  | L7 | 18,635,058 |  | 18,293,421 |  | 15,253,013 |
|  |  | L8 | 16,877,217 |  | 16,569,750 |  | 13,758,441 |
|  |  |  |  |  |  |  |  |
|  | R2 | L6 | 18,560,892 |  | 20,825,984 |  | 18,866,595 |
|  |  | L7 | 18,055,118 |  | 20,369,910 |  | 18,359,725 |
|  |  | L8 | 16,370,779 |  | 18,390,439 |  | 16,525,123 |
|  |  |  |  |  |  |  |  |
|  | R3 | L6 | 20,766,822 |  | 20,744,350 |  | 18,523,493 |
|  |  | L7 | 20,037,172 |  | 20,274,087 |  | 17,964,974 |
|  |  | L8 | 18,132,341 |  | 18,319,681 |  | 16,285,823 |
|  |  |  |  |  |  |  |  |
| ATLWT | R1 | L6 | 20,124,230 |  | 18,368,892 |  | 17,039,622 |
|  |  | L7 | 19,553,207 |  | 17,934,066 |  | 16,670,954 |
|  |  | L8 | 17659702 |  | 16,263,302 |  | 15,068,747 |
|  |  |  |  |  |  |  |  |
|  | R2 | L6 | 18,421,604 |  | 16,523,128 |  | 17,582,811 |
|  |  | L7 | 18,011,909 |  | 16,185,141 |  | 17,050,799 |
|  |  | L8 | 16,282,447 |  | 14,597,196 |  | 15,508,626 |
|  |  |  |  |  |  |  |  |
|  | R3 | L6 | 19,459,082 |  | 19,836,060 |  | 14,875,270 |
|  |  | L7 | 18,952,052 |  | 19,418,110 |  | 14,667,737 |
|  |  | L8 | 17,148,179 |  | 17,560,442 |  | 13,231,279 |
