# Supplementary material for: Overexpression of a modified eIF4E regulates potato virus Y resistance at the transcriptional level in potato
Source: BMC Genomics. 2020 Jan 6;21:18. doi: 10.1186/s12864-019-6423-5 (PMC6945410; doi:10.1186/s12864-019-6423-5)
Supplement: Supplementary file 4 — Additional file 4 : Table S2. Abundance of eIF4E sequences in each sample in Transcripts per million (TPM) and Reads per Kilobase per Million (RPKM, in parentheses). [file 12864_2019_6423_MOESM4_ESM.docx]

**Additional Table 2.** Abundance of eIF4E sequences in each sample in Transcripts per million (TPM) and Reads per Kilobase per Million (RPKM, in parentheses).

|  |  |  | ATLWT |  |  |  | ATL07 |  |  |
| --- | --- | --- | --- | --- | --- | --- | --- | --- | --- |
|  |  | Mock | PVY^N:O^ | PVY^O^ |  | Mock | PVY^N:O^ | PVY^O^ |  |
|  |  |  |  |  |  |  |  |  |  |
| Rep1 | L6 | 59.2 (45.6) | 47.7 (36.2) | 31.1 (21.5) |  | 267.1 (213.1) | 220.4 (174.8) | 239.4 (193.9) |  |
|  | L7 | 65.2 (50.3) | 51.4 (39.0) | 37.2 (25.8) |  | 258.5 (206.5) | 210.9 (167.4) | 243.9 (197.7) |  |
|  | L8 | 55.4 (42.7) | 49.2 (37.3) | 35.2 (24.3) |  | 273.5 (218.2) | 209.5 (166.1) | 234.7 (190.0) |  |
|  |  |  |  |  |  |  |  |  |  |
| Rep2 | L6 | 55.4 (42.8) | 50.2 (36.2) | 41.6 (28.5) |  | 158.9 (117.7) | 233.7 (186.0) | 222.7 (173.6) |  |
|  | L7 | 57.9 (44.8) | 56.2 (40.6) | 42.0 (28.8) |  | 151.7 (112.5) | 233.6 (186.1) | 219.4 (171.1) |  |
|  | L8 | 56.7 (43.8) | 45.2 (32.6) | 46.7 (32.0) |  | 154.2 (114.2) | 236.4 (188.1) | 237.7 (185.3) |  |
|  |  |  |  |  |  |  |  |  |  |
| Rep3 | L6 | 58.8 (44.6) | 46.2 (32.5) | 44.5 (33.0) |  | 302.1 (232.6) | 178.0 (141.6) | 255.0 (206.3) |  |
|  | L7 | 62.5 (47.5) | 42.3 (29.9) | 42.6 (31.6) |  | 287.0 (221.2) | 176.0 (140.2) | 240.1 (194.9) |  |
|  | L8 | 63.6 (48.3) | 44.5 (31.4) | 40.0 (29.7) |  | 294.0 (226.4) | 175.6 (139.8) | 250.2 (202.5) |  |
