# Supplementary material for: Overexpression of a modified eIF4E regulates potato virus Y resistance at the transcriptional level in potato
Source: BMC Genomics. 2020 Jan 6;21:18. doi: 10.1186/s12864-019-6423-5 (PMC6945410; doi:10.1186/s12864-019-6423-5)
Supplement: Supplementary file 5 — Additional file 5 : Table S3. Table of counts for each nucleotide at the potato4E:pvr12 sites in the eIF4E assembly for each data set. [file 12864_2019_6423_MOESM5_ESM.docx]

**Additional Table 3.** Table of counts for each nucleotide at the potato4E:pvr12 sites in the eIF4E assembly for each data set.

|  |  | **Position 209** | | | | | | | | |  | **Position 245** | | | | | | | | |  | **Position 334** | | | | | | | | |
| --- | --- | --- | --- | --- | --- | --- | --- | --- | --- | --- | --- | --- | --- | --- | --- | --- | --- | --- | --- | --- | --- | --- | --- | --- | --- | --- | --- | --- | --- | --- |
|  |  | **ATLO7** | | | |  | **ATLWT** | | | |  | **ATLO7** | | | |  | **ALTWT** | | | |  | **ATLO7** | | | |  | **ATLWT** | | | |
|  |  | **A** | **C** | **G** | **T** |  | **A** | **C** | **G** | **T** |  | **A** | **C** | **G** | **T** |  | **A** | **C** | **G** | **T** |  | **A** | **C** | **G** | **T** |  | **A** | **C** | **G** | **T** |
| **Mock** |  |  |  |  |  |  |  |  |  |  |  |  |  |  |  |  |  |  |  |  |  |  |  |  |  |  |  |  |  |  |
| R1 |  | 185 | 0 | 0 | 3 |  | 1 | 0 | 0 | 24 |  | 0 | 0 | 163 | 1 |  | 0 | 0 | 0 | 29 |  | 88 | 0 | 4 | 0 |  | 0 | 0 | 45 | 0 |
|  |  | 97 | 0 | 0 | 0 |  | 0 | 0 | 0 | 17 |  | 0 | 1 | 63 | 1 |  | 0 | 0 | 0 | 17 |  | 58 | 0 | 3 | 0 |  | 1 | 0 | 32 | 1 |
|  |  | 213 | 0 | 0 | 3 |  | 0 | 0 | 0 | 37 |  | 0 | 0 | 172 | 7 |  | 0 | 0 | 0 | 26 |  | 152 | 0 | 8 | 0 |  | 0 | 0 | 30 | 0 |
|  |  |  |  |  |  |  |  |  |  |  |  |  |  |  |  |  |  |  |  |  |  |  |  |  |  |  |  |  |  |  |
| R2 |  | 182 | 0 | 0 | 1 |  | 0 | 0 | 0 | 29 |  | 0 | 0 | 141 | 1 |  | 0 | 0 | 0 | 20 |  | 94 | 0 | 1 | 0 |  | 0 | 0 | 38 | 0 |
|  |  | 79 | 0 | 0 | 2 |  | 0 | 0 | 0 | 26 |  | 0 | 0 | 53 | 0 |  | 0 | 0 | 0 | 27 |  | 50 | 0 | 2 | 0 |  | 0 | 0 | 34 | 0 |
|  |  | 186 | 1 | 0 | 8 |  | 0 | 0 | 0 | 30 |  | 0 | 1 | 190 | 6 |  | 0 | 0 | 0 | 34 |  | 122 | 0 | 2 | 0 |  | 0 | 0 | 49 | 0 |
|  |  |  |  |  |  |  |  |  |  |  |  |  |  |  |  |  |  |  |  |  |  |  |  |  |  |  |  |  |  |  |
| R3 |  | 147 | 0 | 0 | 6 |  | 0 | 0 | 0 | 28 |  | 0 | 0 | 113 | 2 |  | 0 | 0 | 1 | 22 |  | 83 | 0 | 3 | 0 |  | 0 | 0 | 32 | 0 |
|  |  | 65 | 0 | 0 | 1 |  | 0 | 0 | 0 | 20 |  | 0 | 0 | 59 | 1 |  | 0 | 0 | 0 | 23 |  | 40 | 1 | 4 | 0 |  | 0 | 0 | 21 | 0 |
|  |  | 165 | 1 | 0 | 5 |  | 0 | 0 | 0 | 25 |  | 0 | 0 | 172 | 1 |  | 0 | 0 | 0 | 20 |  | 101 | 1 | 7 | 0 |  | 0 | 0 | 41 | 0 |
|  |  |  |  |  |  |  |  |  |  |  |  |  |  |  |  |  |  |  |  |  |  |  |  |  |  |  |  |  |  |  |
|  |  |  |  |  |  |  |  |  |  |  |  |  |  |  |  |  |  |  |  |  |  |  |  |  |  |  |  |  |  |  |
| **PVY^N:O^** |  |  |  |  |  |  |  |  |  |  |  |  |  |  |  |  |  |  |  |  |  |  |  |  |  |  |  |  |  |  |
| R1 |  | 150 | 0 | 0 | 2 |  | 0 | 0 | 0 | 28 |  | 0 | 0 | 127 | 2 |  | 0 | 0 | 0 | 24 |  | 93 | 0 | 4 | 0 |  | 0 | 0 | 27 | 0 |
|  |  | 153 | 0 | 0 | 3 |  | 0 | 0 | 0 | 34 |  | 0 | 0 | 118 | 9 |  | 0 | 0 | 0 | 27 |  | 105 | 0 | 4 | 0 |  | 1 | 0 | 28 | 0 |
|  |  | 128 | 1 | 0 | 3 |  | 0 | 0 | 0 | 20 |  | 0 | 0 | 102 | 8 |  | 0 | 0 | 1 | 27 |  | 99 | 0 | 3 | 0 |  | 0 | 0 | 23 | 0 |
|  |  |  |  |  |  |  |  |  |  |  |  |  |  |  |  |  |  |  |  |  |  |  |  |  |  |  |  |  |  |  |
| R2 |  | 143 | 0 | 0 | 0 |  | 1 | 0 | 0 | 40 |  | 0 | 0 | 97 | 2 |  | 0 | 0 | 0 | 32 |  | 82 | 0 | 2 | 0 |  | 0 | 1 | 34 | 1 |
|  |  | 163 | 0 | 0 | 0 |  | 0 | 0 | 0 | 33 |  | 0 | 1 | 125 | 4 |  | 0 | 0 | 0 | 45 |  | 87 | 0 | 7 | 0 |  | 0 | 0 | 29 | 0 |
|  |  | 123 | 0 | 0 | 4 |  | 0 | 0 | 0 | 12 |  | 0 | 0 | 84 | 0 |  | 0 | 0 | 0 | 21 |  | 68 | 0 | 3 | 0 |  | 0 | 0 | 18 | 0 |
|  |  |  |  |  |  |  |  |  |  |  |  |  |  |  |  |  |  |  |  |  |  |  |  |  |  |  |  |  |  |  |
| R3 |  | 130 | 0 | 0 | 1 |  | 0 | 0 | 0 | 31 |  | 0 | 0 | 90 | 1 |  | 0 | 0 | 1 | 29 |  | 87 | 0 | 0 | 0 |  | 1 | 0 | 38 | 0 |
|  |  | 140 | 0 | 0 | 4 |  | 0 | 0 | 0 | 28 |  | 0 | 1 | 127 | 4 |  | 0 | 0 | 0 | 27 |  | 96 | 0 | 1 | 0 |  | 0 | 0 | 15 | 0 |
|  |  | 107 | 0 | 0 | 3 |  | 0 | 0 | 0 | 16 |  | 0 | 0 | 72 | 2 |  | 0 | 0 | 0 | 8 |  | 56 | 1 | 1 | 0 |  | 0 | 0 | 18 | 0 |
|  |  |  |  |  |  |  |  |  |  |  |  |  |  |  |  |  |  |  |  |  |  |  |  |  |  |  |  |  |  |  |
| **PVY^O^** |  |  |  |  |  |  |  |  |  |  |  |  |  |  |  |  |  |  |  |  |  |  |  |  |  |  |  |  |  |  |
| R1 |  | 149 | 0 | 0 | 5 |  | 0 | 0 | 0 | 11 |  | 0 | 0 | 120 | 4 |  | 0 | 0 | 0 | 13 |  | 107 | 0 | 1 | 0 |  | 0 | 0 | 10 | 0 |
|  |  | 192 | 0 | 0 | 4 |  | 0 | 0 | 0 | 10 |  | 0 | 0 | 96 | 2 |  | 0 | 0 | 0 | 22 |  | 86 | 0 | 5 | 0 |  | 0 | 0 | 21 | 0 |
|  |  | 123 | 0 | 0 | 3 |  | 0 | 0 | 0 | 31 |  | 0 | 0 | 105 | 2 |  | 0 | 0 | 0 | 13 |  | 97 | 0 | 2 | 0 |  | 0 | 0 | 26 | 0 |
|  |  |  |  |  |  |  |  |  |  |  |  |  |  |  |  |  |  |  |  |  |  |  |  |  |  |  |  |  |  |  |
| R2 |  | 149 | 0 | 0 | 1 |  | 0 | 0 | 0 | 15 |  | 0 | 0 | 113 | 5 |  | 0 | 0 | 1 | 15 |  | 108 | 0 | 2 | 0 |  | 0 | 0 | 19 | 0 |
|  |  | 136 | 1 | 0 | 2 |  | 2 | 0 | 0 | 9 |  | 0 | 0 | 99 | 3 |  | 0 | 0 | 0 | 24 |  | 67 | 0 | 10 | 0 |  | 0 | 0 | 10 | 0 |
|  |  | 139 | 0 | 0 | 2 |  | 1 | 0 | 0 | 28 |  | 0 | 0 | 83 | 2 |  | 0 | 0 | 0 | 21 |  | 91 | 0 | 2 | 0 |  | 0 | 0 | 18 | 0 |
|  |  |  |  |  |  |  |  |  |  |  |  |  |  |  |  |  |  |  |  |  |  |  |  |  |  |  |  |  |  |  |
| R3 |  | 130 | 0 | 0 | 2 |  | 0 | 0 | 0 | 5 |  | 0 | 0 | 92 | 2 |  | 0 | 0 | 0 | 7 |  | 116 | 0 | 0 | 0 |  | 0 | 0 | 21 | 0 |
|  |  | 131 | 0 | 0 | 4 |  | 0 | 0 | 0 | 8 |  | 0 | 0 | 107 | 3 |  | 0 | 0 | 0 | 16 |  | 77 | 0 | 9 | 0 |  | 1 | 0 | 25 | 0 |
|  |  | 105 | 1 | 0 | 4 |  | 0 | 0 | 1 | 28 |  | 0 | 0 | 74 | 4 |  | 0 | 0 | 0 | 10 |  | 74 | 0 | 1 | 0 |  | 1 | 0 | 18 | 0 |
