# Supplementary material for: Overexpression of a modified eIF4E regulates potato virus Y resistance at the transcriptional level in potato
Source: BMC Genomics. 2020 Jan 6;21:18. doi: 10.1186/s12864-019-6423-5 (PMC6945410; doi:10.1186/s12864-019-6423-5)
Supplement: Supplementary file 6 — Additional file 6 : Table S4. Abundance of PVY sequences in each sample in Transcripts per million (TPM) and Reads per Kilobase per Million (RPKM, in parentheses). [file 12864_2019_6423_MOESM6_ESM.docx]

**Additional Table 4.** Abundance of PVY sequences in each sample in Transcripts per million (TPM) and Reads per Kilobase per Million (RPKM, in parentheses).

|  |  |  | ATLWT |  |  |  | ATL07 |  |
| --- | --- | --- | --- | --- | --- | --- | --- | --- |
|  |  | Mock | PVY^N:O^ | PVY^O^ |  | Mock | PVY^N:O^ | PVY^O^ |
|  |  |  |  |  |  |  |  |  |
| Rep1 | L6 | 15.1 (11.6) | **10,043.0 (7,614.8)** | **20,051.6 (13,878.5)** |  | 13.5 (10.7) | 19.9 (15.7) | 13.6 (11.0) |
|  | L7 | 14.8 (11.4) | **10,026.2 (7,605.3)** | **19,941.2 (13,822.7)** |  | 13.4 (10.7) | 16.9 (13.4) | 12.4 (10.1) |
|  | L8 | 16.2 (12.5) | **10,114.9 (7,664.4)** | **20,107.5 (13,908.5)** |  | 13.0 (10.4) | 21.4 (17.0) | 12.8 (10.4) |
|  |  |  |  |  |  |  |  |  |
| Rep2 | L6 | 14.8 (11.4) | **14,774.8 (10,663.4)** | **20,069.7 (13,750.0)** |  | 35.7 (26.4) | 14.3 (11.4) | 15.7 (12.3) |
|  | L7 | 14.0 (10.9) | **14,694.4 (10,619.7)** | **19,926.9 (13,684.3)** |  | 35.7 (26.5) | 13.8 (11.0) | 15.4 (12.1) |
|  | L8 | 16.4 (12.7) | **14,744.1 (10,640.1)** | **20,108.7 (13,775.4)** |  | 34.1 (25.2) | 15.3 (12.1) | 15.2 (11.8) |
|  |  |  |  |  |  |  |  |  |
| Rep3 | L6 | 18.2 (13.8) | **13,329.5 (9,395.0)** | **17,318.3 (12,835.8)** |  | 21.6 (16.6) | 12.4 (9.80) | 17.5 (14.2) |
|  | L7 | 16.8 (12.7) | **13,280.1 (9,377.3)** | **17,288.6 (12,816.6)** |  | 21.1 (16.3) | 12.9 (10.2) | 16.3 (13.2) |
|  | L8 | 17.7 (13.4) | **13,338.9 (9,407.9)** | **17,390.7 (12,877.6)** |  | 21.4 (16.5) | 13.5 (10.7) | 18.6 (15.1) |
