# Supplementary material for: Overexpression of a modified eIF4E regulates potato virus Y resistance at the transcriptional level in potato
Source: BMC Genomics. 2020 Jan 6;21:18. doi: 10.1186/s12864-019-6423-5 (PMC6945410; doi:10.1186/s12864-019-6423-5)
Supplement: Supplementary file 7 — Additional file 7 : Table S5. Differentially expressed genes in various comparisons [file 12864_2019_6423_MOESM7_ESM.docx]

**Additional Table 5. Differentially expressed genes in various comparisons**

**A. Upregulated genes between ATLWT (Mock) and ATL07(Mock)**

| Rank | GeneID | LogFC | LogCPM | F | P_value | Annotation | RNAtype |
| --- | --- | --- | --- | --- | --- | --- | --- |
|  |  |  |  |  |  |  |  |
| 1 | 102600818 | 3.576 | 5.053 | 1046.181 | 2.16E-15 | uncharacterized(LOC102600818) | mRNA |
| **2** | **102580433** | **2.4** | **6.279** | **881.345** | **7.78E-15** | **eukaryotic_translation_initiation_factor_4E(EIF4E)** | **mRNA** |
| 3 | 102601624 | 2.019 | 4.493 | 431.029 | 1.54E-12 | transcription_factor_BIM1-like(LOC102601624) | mRNA |
| 4 | 102603641 | 3.873 | 4.779 | 426.775 | 1.85E-12 | uncharacterized(LOC102603641) | mRNA |
| 5 | 102603857 | 2.631 | 5.043 | 378.945 | 4.00E-12 | premnaspirodiene_oxygenase(LOC102603857) | mRNA |
| 6 | 102582832 | 2.259 | 7.444 | 304.928 | 1.92E-11 | zinc_finger_protein_CONSTANS-LIKE_5-like(LOC102582832) | mRNA |
| 7 | 107061417 | 2.556 | 2.939 | 269.019 | 4.73E-11 | ABC_transporter_G_family_member_11-like(LOC107061417) | mRNA |
| 8 | 102603146 | 2.01 | 3.287 | 231.97 | 1.37E-10 | F-box/kelch-repeat_protein_SKIP25-like(LOC102603146) | mRNA |
| 9 | 102602737 | 2.322 | 7.021 | 214.695 | 2.39E-10 | ABC_transporter_G_family_member_11-like(LOC102602737) | mRNA |
| 10 | 102581374 | 3.086 | 6.756 | 214.557 | 2.62E-10 | aquaporin_PIP1-1(LOC102581374) | mRNA |
| 11 | 102579771 | 2.54 | 5.848 | 212.337 | 2.79E-10 | mitochondrial_uncoupling_protein_5-like(LOC102579771) | mRNA |
| 12 | 102587493 | 3.185 | 4.889 | 197.762 | 4.68E-10 | zeatin_O-glucosyltransferase-like(LOC102587493) | mRNA |
| 13 | 102581540 | 3.407 | 5.093 | 193.678 | 5.41E-10 | zeatin_O-glucosyltransferase-like(LOC102581540) | mRNA |
| 14 | 102606006 | 2.354 | 2.674 | 166.433 | 1.43E-09 | UDP-glucose_iridoid_glucosyltransferase-like(LOC102606006) | mRNA |
| 15 | 102589062 | 2.326 | 2.319 | 166.034 | 1.45E-09 | probable_purine_permease_11(LOC102589062) | mRNA |
| 16 | 102580309 | 2.029 | 1.261 | 165.121 | 1.51E-09 | protein_NLP7-like(LOC102580309) | mRNA |
| 17 | 102603519 | 2.334 | 3.138 | 165.132 | 1.52E-09 | premnaspirodiene_oxygenase-like(LOC102603519) | mRNA |
| 18 | 102590117 | 2.376 | 5.682 | 161.66 | 1.90E-09 | malate_synthase_glyoxysomal-like(LOC102590117) | mRNA |
| 19 | 102598120 | 2.155 | 2.996 | 154.813 | 2.36E-09 | type_I_inositol_polyphosphate_5-phosphatase_2-like(LOC102598120) | mRNA |
| 20 | 102583768 | 3.613 | 3.25 | 145.412 | 3.95E-09 | zeatin_O-glucosyltransferase-like(LOC102583768) | mRNA |
| 21 | 107061001 | 5.346 | 0.777 | 134.757 | 6.14E-09 | protein_RADIALIS-like_1(LOC107061001) | mRNA |
| 22 | 102579118 | 2.769 | 2.619 | 135.437 | 6.15E-09 | protein_BIG_GRAIN_1-like_A(LOC102579118) | mRNA |
| 23 | 107060856 | 2.398 | 4.653 | 126.952 | 9.95E-09 | uncharacterized(LOC107060856) | mRNA |
| 24 | 102590788 | 2.254 | 1.784 | 120.702 | 1.30E-08 | F-box_protein_SKIP27-like(LOC102590788) | mRNA |
| 25 | 102583000 | 2.633 | 5.302 | 119.815 | 1.47E-08 | WAT1-related_protein_At1g70260-like(LOC102583000) | mRNA |
| 26 | 102581470 | 2.936 | 6.214 | 113.846 | 2.07E-08 | cytokinin_riboside_5'-monophosphate_phosphoribohydrolase_LOG3-like(LOC102581470) | mRNA |
| 27 | 102603317 | 2.373 | 7.998 | 111.391 | 2.36E-08 | titin(LOC102603317) | mRNA |
| 28 | 102589598 | 4.102 | 1.044 | 106.933 | 2.95E-08 | mitogen-activated_protein_kinase_kinase_kinase_NPK1-like(LOC102589598) | mRNA |
| 29 | 102601536 | 2.028 | 5.848 | 101.645 | 4.40E-08 | protein_LURP-one-related_10-like(LOC102601536) | mRNA |
| 30 | 107061574 | 3.115 | 0.208 | 95.802 | 6.09E-08 | uncharacterized(LOC107061574) | mRNA |
| 31 | 107061418 | 2.989 | 2.278 | 95.687 | 6.47E-08 | uncharacterized(LOC107061418) | mRNA |
| 32 | 102581622 | 2.015 | 2.89 | 88.599 | 1.05E-07 | delta(8)-fatty-acid_desaturase-like(LOC102581622) | mRNA |
| 33 | 102590175 | 2.252 | 1.329 | 86.049 | 1.23E-07 | lachrymatory-factor_synthase(LOC102590175) | mRNA |
| 34 | 107063292 | 3.348 | 2.481 | 85.297 | 1.38E-07 | uncharacterized(LOC107063292) | ncRNA |
| 35 | 102597555 | 2.464 | 0.512 | 84.207 | 1.42E-07 | type_I_inositol_polyphosphate_5-phosphatase_12-like(LOC102597555) | mRNA |
| 36 | 102589935 | 3.521 | 1.698 | 84.513 | 1.46E-07 | mitogen-activated_protein_kinase_kinase_kinase_NPK1-like(LOC102589935) | mRNA |
| 1 | 102577493 | 2.026 | 2.757 | 80.828 | 1.90E-07 | cytokinin_oxidase/dehydrogenase(CKX3) | mRNA |
| 2 | 102597935 | 2.449 | 5.871 | 81.075 | 1.92E-07 | protein_STAY-GREEN_chloroplastic-like(LOC102597935) | mRNA |
| 3 | 107057655 | 4.847 | 3.358 | 79.905 | 2.10E-07 | probable_xyloglucan_endotransglucosylase/hydrolase_protein_23(LOC107057655) | mRNA |
| 4 | 102594317 | 2.313 | 1.922 | 79.004 | 2.16E-07 | pirin-like_protein_At1g50590(LOC102594317) | mRNA |
| 5 | 102605914 | 2.032 | 3.362 | 79.243 | 2.21E-07 | putative_UPF0481_protein_At3g02645(LOC102605914) | mRNA |
| 6 | 107057674 | 2.758 | 0.733 | 74.43 | 3.13E-07 | glyceraldehyde-3-phosphate_dehydrogenase_A_chloroplastic-like(LOC107057674) | mRNA |
| 7 | 102605953 | 2.263 | 2.526 | 73.004 | 3.69E-07 | GDSL_esterase/lipase_At5g55050-like(LOC102605953) | mRNA |
| 8 | 102592929 | 3.032 | 0.999 | 72.256 | 3.78E-07 | uncharacterized(LOC102592929) | mRNA |
| 9 | 102577728 | 3.645 | 8.26 | 71.176 | 4.38E-07 | NADH_nitrate_reductase(NR3) | mRNA |
| 10 | 102598420 | 4.323 | 1.533 | 71.13 | 4.40E-07 | probable_xyloglucan_endotransglucosylase/hydrolase_protein_23(LOC102598420) | mRNA |
| 11 | 102605361 | 2.137 | 3.648 | 70.426 | 4.68E-07 | secoisolariciresinol_dehydrogenase-like(LOC102605361) | mRNA |
| 12 | 107060721 | 2.136 | 1.257 | 67.838 | 5.62E-07 | uncharacterized_N-acetyltransferase_p20-like(LOC107060721) | mRNA |
| 13 | 102594297 | 2.951 | 5.597 | 65.484 | 7.37E-07 | S-norcoclaurine_synthase_1-like(LOC102594297) | mRNA |
| 14 | 102578349 | 2.401 | 0.935 | 64.02 | 8.07E-07 | uncharacterized(LOC102578349) | ncRNA |
| 15 | 107062285 | 2.738 | 2.376 | 63.587 | 8.84E-07 | uncharacterized(LOC107062285) | mRNA |
| 16 | 102592061 | 2.833 | 0.349 | 60.91 | 1.10E-06 | thaumatin-like_protein_1(LOC102592061) | mRNA |
| 17 | 102587688 | 3.066 | 1.178 | 59.12 | 1.35E-06 | transcription_factor_RADIALIS(LOC102587688) | mRNA |
| 18 | 102583661 | 2.783 | 3.498 | 58.655 | 1.45E-06 | auxin-responsive_protein_IAA20-like(LOC102583661) | mRNA |
| 19 | 102586638 | 2.042 | 3.349 | 57.947 | 1.56E-06 | protein_ENHANCED_DISEASE_RESISTANCE_2(LOC102586638) | mRNA |
| 20 | 102600705 | 2.743 | 1.214 | 55.696 | 1.92E-06 | E3_ubiquitin-protein_ligase_ATL42-like(LOC102600705) | mRNA |
| 21 | 102581856 | 2.391 | 4.791 | 51.095 | 3.31E-06 | protein_NRT1/_PTR_FAMILY_4.6-like(LOC102581856) | mRNA |
| 22 | 102598017 | 2.012 | 1.759 | 50.615 | 3.40E-06 | cytochrome_P450_78A6-like(LOC102598017) | mRNA |
| 23 | 102590478 | 2.996 | 0.452 | 49.315 | 3.92E-06 | uncharacterized(LOC102590478) | mRNA |
| 24 | 102591566 | 2.011 | 2.447 | 46.989 | 5.37E-06 | (-)-camphene/tricyclene_synthase_chloroplastic-like(LOC102591566) | mRNA |
| 25 | 102598758 | 4.633 | 2.116 | 45.671 | 6.37E-06 | probable_xyloglucan_endotransglucosylase/hydrolase_protein_23(LOC102598758) | mRNA |
| 26 | 102592249 | 2.799 | 1.271 | 45.168 | 6.73E-06 | uncharacterized(LOC102592249) | ncRNA |
| 27 | 107059000 | 2.068 | 0.911 | 44.65 | 6.99E-06 | transcription_factor_MYB90-like(LOC107059000) | mRNA |
| 28 | 102598791 | 2.747 | 1.607 | 44.776 | 7.13E-06 | NAC_domain-containing_protein_90-like(LOC102598791) | mRNA |
| 29 | 102593554 | 2.274 | 2.504 | 44.551 | 7.34E-06 | probable_esterase_D14L(LOC102593554) | mRNA |
| 30 | 102598548 | 2.8 | 1.216 | 44.15 | 7.66E-06 | AAA-ATPase_At3g28600-like(LOC102598548) | mRNA |
| 31 | 102584686 | 3.24 | 4.395 | 41.629 | 1.08E-05 | probable_pectinesterase/pectinesterase_inhibitor_12(LOC102584686) | mRNA |
| 32 | 102597674 | 2.493 | 5.005 | 40.482 | 1.26E-05 | pyruvate_kinase_isozyme_G_chloroplastic(LOC102597674) | mRNA |
| 33 | 102599527 | 4.163 | 1.328 | 40.299 | 1.30E-05 | probable_xyloglucan_endotransglucosylase/hydrolase_protein_23(LOC102599527) | mRNA |
| 34 | 102586241 | 2.087 | 8.904 | 38.171 | 1.75E-05 | chlorophyll_a-b_binding_protein_40_chloroplastic(LOC102586241) | mRNA |
| 35 | 102580263 | 2.028 | 4.515 | 36.044 | 2.40E-05 | uncharacterized(LOC102580263) | mRNA |
| 36 | 102593879 | 2.345 | 0.794 | 35.212 | 2.64E-05 | phosphatidylinositol/phosphatidylcholine_transfer_protein_SFH12-like(LOC102593879) | mRNA |
| 37 | 102590956 | 2.848 | 3.474 | 35.316 | 2.68E-05 | uncharacterized(LOC102590956) | mRNA |
| 38 | 102578498 | 3.397 | 1.607 | 34.798 | 2.90E-05 | flowering-promoting_factor_1-like_protein_3(LOC102578498) | mRNA |
| 39 | 102597519 | 3.755 | 3.912 | 34.736 | 2.92E-05 | (-)-germacrene_D_synthase-like(LOC102597519) | mRNA |
| 40 | 102599254 | 3.552 | 3.881 | 34.359 | 3.10E-05 | valencene_synthase-like(LOC102599254) | mRNA |
| 41 | 102580361 | 2.221 | 2.119 | 32.421 | 4.22E-05 | protein_EXORDIUM-like(LOC102580361) | mRNA |
| 42 | 102603101 | 2.01 | 1.592 | 31.709 | 4.72E-05 | probable_galacturonosyltransferase-like_10(LOC102603101) | mRNA |
| 43 | 102602408 | 2.877 | 1.526 | 29.532 | 6.86E-05 | probable_calcium-binding_protein_CML30(LOC102602408) | mRNA |
| 44 | 102585742 | 2.767 | 0.88 | 29.076 | 7.39E-05 | uncharacterized_acetyltransferase_At3g50280-like(LOC102585742) | mRNA |
| 45 | 102589439 | 2.345 | 5.485 | 28.696 | 7.94E-05 | urea-proton_symporter_DUR3-like(LOC102589439) | mRNA |
| 46 | 102581792 | 2.423 | 1.245 | 28.161 | 8.72E-05 | gibberellin_20_oxidase_1(LOC102581792) | mRNA |
| 47 | 102585893 | 2.18 | 5.346 | 27.754 | 9.40E-05 | phylloplanin-like(LOC102585893) | mRNA |
| 48 | 107061554 | 2.724 | 2.047 | 26.512 | 1.18E-04 | uncharacterized(LOC107061554) | mRNA |
| 49 | 102599063 | 4.301 | 0.508 | 26.104 | 1.27E-04 | uncharacterized(LOC102599063) | mRNA |
| 50 | 102601336 | 2.705 | 0.944 | 25.978 | 1.30E-04 | probable_LRR_receptor-like_serine/threonine-protein_kinase_At3g47570(LOC102601336) | mRNA |
| 51 | 102580715 | 2.166 | 4.303 | 24.45 | 1.75E-04 | protein_ASPARTIC_PROTEASE_IN_GUARD_CELL_1-like(LOC102580715) | mRNA |
| 52 | 102592722 | 2.269 | 4.029 | 24.136 | 1.87E-04 | cytochrome_b561_and_DOMON_domain-containing_protein_At3g25290-like(LOC102592722) | mRNA |
| 53 | 102581872 | 4.946 | 0.048 | 23.696 | 2.04E-04 | protein_ECERIFERUM_3-like(LOC102581872) | mRNA |
| 54 | 107058426 | 3.051 | 0.027 | 23.194 | 2.23E-04 | acetyl-CoA-benzylalcohol_acetyltransferase-like(LOC107058426) | mRNA |
| 55 | 102604988 | 2.795 | 1.59 | 22.91 | 2.39E-04 | 14_kDa_proline-rich_protein_DC2.15-like(LOC102604988) | mRNA |
| 56 | 102579194 | 3.826 | 2.478 | 22.751 | 2.47E-04 | protein_FLOWERINGT-like(LOC102579194) | mRNA |
| 57 | 102587711 | 4.386 | 0.234 | 22.572 | 2.56E-04 | MADS-box_protein_CMB1(AP1) | mRNA |
| 58 | 102598327 | 2.527 | 2.316 | 22.171 | 2.78E-04 | pectinesterase_3(LOC102598327) | mRNA |
| 59 | 102584653 | 2.097 | 5.722 | 21.872 | 2.97E-04 | protein_CDI-like(LOC102584653) | mRNA |
| 60 | 102582189 | 2.414 | 5.014 | 20.584 | 3.91E-04 | NAD(P)H:quinone_oxidoreductase-like(LOC102582189) | mRNA |
| 61 | 102580526 | 2.367 | 4.213 | 20.517 | 3.97E-04 | abscisic_acid_receptor_PYL4-like(LOC102580526) | mRNA |
| 62 | 102577568 | 3.841 | 2.045 | 20.242 | 4.22E-04 | CYP86A33_fatty_acid_omega-hydroxylase(LOC102577568) | mRNA |
| 63 | 102585105 | 2.011 | 4.364 | 18.91 | 5.70E-04 | NAC_transcription_factor_29(LOC102585105) | mRNA |
| 64 | 102605396 | 3.748 | 1.131 | 18.844 | 5.79E-04 | F-box_protein_CPR30-like(LOC102605396) | mRNA |
| 65 | 102584425 | 2.1 | 5.782 | 18.249 | 6.66E-04 | probable_isoaspartyl_peptidase/L-asparaginase_2(LOC102584425) | mRNA |
| 66 | 102585621 | 4.94 | 0.16 | 18.181 | 6.76E-04 | ABC_transporter_G_family_member_11-like(LOC102585621) | mRNA |
| 67 | 102579394 | 3.589 | -0.249 | 18.06 | 6.94E-04 | 3-ketoacyl-CoA_synthase_6-like(LOC102579394) | mRNA |
| 68 | 102597748 | 3.212 | 1.851 | 17.415 | 8.13E-04 | laccase-3-like(LOC102597748) | mRNA |
| 69 | 107059919 | 2.162 | 2.287 | 17.174 | 8.62E-04 | probably_inactive_leucine-rich_repeat_receptor-like_protein_kinase_At5g48380(LOC107059919) | mRNA |
| 70 | 102593925 | 2.17 | 3.659 | 17.008 | 8.98E-04 | thioredoxin_H2-like(LOC102593925) | mRNA |
| 71 | 102580035 | 2.219 | 1.036 | 16.964 | 9.07E-04 | protein_EXORDIUM-like(LOC102580035) | mRNA |
| 72 | 102578612 | 2.166 | 2.408 | 16.897 | 9.22E-04 | probably_inactive_leucine-rich_repeat_receptor-like_protein_kinase_At5g48380(LOC102578612) | mRNA |
| 73 | 102582497 | 4.044 | 0.875 | 16.605 | 9.92E-04 | GDSL_esterase/lipase_At5g37690(LOC102582497) | mRNA |

**B. Downregulated genes between ATLWT (Mock) and ATL07(Mock)**

| Rank | GeneID | LogFC | LogCPM | F | P_value | Annotation | RNAtype |
| --- | --- | --- | --- | --- | --- | --- | --- |
|  |  |  |  |  |  |  |  |
| 1 | 102601020 | -2.034 | 5.109 | 528.474 | 3.45E-13 | BURP_domain-containing_protein_5-like(LOC102601020) | mRNA |
| 2 | 102577492 | -2.099 | 3.491 | 308.89 | 1.75E-11 | ferritin(LOC102577492) | mRNA |
| 3 | 102586322 | -6.272 | 2.017 | 296.535 | 2.35E-11 | phosphatidylinositol:ceramide_inositolphosphotransferase_1-like(LOC102586322) | mRNA |
| 4 | 102606176 | -6.118 | 1.869 | 236.24 | 1.21E-10 | uncharacterized(LOC102606176) | ncRNA |
| 5 | 102596276 | -3.619 | 4.061 | 212.385 | 2.83E-10 | peroxiredoxin-2E-2_chloroplastic-like(LOC102596276) | mRNA |
| 6 | 102579260 | -2.777 | 1.056 | 108.911 | 2.60E-08 | uncharacterized(LOC102579260) | mRNA |
| 7 | 102593471 | -3.631 | 2.127 | 109.15 | 2.69E-08 | neurofilament_medium_polypeptide-like(LOC102593471) | mRNA |
| 8 | 102602513 | -3.189 | 2.533 | 107.744 | 2.94E-08 | uncharacterized(LOC102602513) | mRNA |
| 9 | 102582221 | -2.38 | 5.003 | 93.036 | 7.87E-08 | chaperone_protein_dnaJ_8_chloroplastic-like(LOC102582221) | mRNA |
| 10 | 102587155 | -2.424 | 4.045 | 88.647 | 1.08E-07 | non-classical_arabinogalactan_protein_30(LOC102587155) | mRNA |
| 11 | 102596698 | -2.782 | 1.445 | 79.668 | 2.03E-07 | uncharacterized(LOC102596698) | mRNA |
| 12 | 102599879 | -2.4 | 0.731 | 68.267 | 5.41E-07 | uncharacterized(LOC102599879) | mRNA |
| 13 | 102605695 | -3.159 | 5.41 | 67.788 | 5.94E-07 | chaperone_protein_dnaJ_20_chloroplastic-like(LOC102605695) | mRNA |
| 14 | 102592011 | -2.326 | 3.051 | 63.787 | 8.67E-07 | vetispiradiene_synthase_3-like(LOC102592011) | mRNA |
| 15 | 102582185 | -2.698 | 1.293 | 58.186 | 1.45E-06 | uncharacterized(LOC102582185) | ncRNA |
| 16 | 102604772 | -2.397 | 1.446 | 56.306 | 1.77E-06 | pentatricopeptide_repeat-containing_protein_At1g56690_mitochondrial-like(LOC102604772) | mRNA |
| 17 | 107059112 | -3.197 | 0.954 | 54.788 | 2.10E-06 | uncharacterized(LOC107059112) | mRNA |
| 18 | 102593025 | -2.195 | 3.937 | 54.297 | 2.31E-06 | vacuolar_amino_acid_transporter_1-like(LOC102593025) | mRNA |
| 19 | 107063266 | -3.118 | 1.131 | 47.954 | 4.68E-06 | uncharacterized(LOC107063266) | mRNA |
| 20 | 102579229 | -3.25 | 1.951 | 47.874 | 4.85E-06 | CEN-like_protein_1(LOC102579229) | mRNA |
| 21 | 107062830 | -3.598 | 0.556 | 45.83 | 6.02E-06 | uncharacterized(LOC107062830) | mRNA |
| 22 | 102586998 | -2.07 | 0.331 | 43.449 | 8.17E-06 | pentatricopeptide_repeat-containing_protein_At4g37170-like(LOC102586998) | mRNA |
| 23 | 102603188 | -2.05 | 4.953 | 43.558 | 8.35E-06 | uncharacterized(LOC102603188) | mRNA |
| 24 | 102590399 | -2.448 | 1.519 | 42.395 | 9.59E-06 | uncharacterized(LOC102590399) | mRNA |
| 25 | 102591281 | -2.315 | 3.146 | 40.928 | 1.19E-05 | uncharacterized(LOC102591281) | mRNA |
| 26 | 102594426 | -3.185 | 3.042 | 40.188 | 1.32E-05 | ankyrin_repeat-containing_protein_At3g12360-like(LOC102594426) | mRNA |
| 27 | 107058888 | -2.435 | 0.855 | 39.931 | 1.32E-05 | uncharacterized(LOC107058888) | ncRNA |
| 28 | 102599939 | -2.767 | 1.151 | 39.489 | 1.42E-05 | classical_arabinogalactan_protein_10-like(LOC102599939) | mRNA |
| 29 | 102581405 | -2.17 | 8.763 | 38.539 | 1.66E-05 | auxin-repressed_12.5_kDa_protein-like(LOC102581405) | mRNA |
| 30 | 107059808 | -3.655 | 0.484 | 37.762 | 1.81E-05 | uncharacterized(LOC107059808) | mRNA |
| 31 | 102597820 | -2.162 | 1.594 | 35.616 | 2.53E-05 | cytochrome_P450_CYP72A219-like(LOC102597820) | mRNA |
| 32 | 102585851 | -2.224 | 1.561 | 35.55 | 2.56E-05 | BTB/POZ_domain-containing_protein_At5g48800(LOC102585851) | mRNA |
| 33 | 102593790 | -3.594 | 0.666 | 34.624 | 2.95E-05 | histone_H2B_7-like(LOC102593790) | mRNA |
| 34 | 102597960 | -2.066 | 3.588 | 33.782 | 3.40E-05 | chitinase-like_protein_1(LOC102597960) | mRNA |
| 35 | 102599410 | -2.737 | 4.815 | 33.705 | 3.44E-05 | uncharacterized(LOC102599410) | mRNA |
| 36 | 102603743 | -2.856 | 4.106 | 33.086 | 3.79E-05 | probable_E3_ubiquitin-protein_ligase_XERICO(LOC102603743) | mRNA |
| 37 | 102594043 | -6.315 | 4.547 | 31.968 | 4.55E-05 | peptidyl-prolyl_cis-trans_isomerase_FKBP62-like(LOC102594043) | mRNA |
| 38 | 107058305 | -3.409 | 0.444 | 31.757 | 4.62E-05 | uncharacterized(LOC107058305) | mRNA |
| 39 | 102585635 | -2.854 | 1.483 | 31.815 | 4.66E-05 | 21_kDa_protein-like(LOC102585635) | mRNA |
| 40 | 102603306 | -2.226 | 1.655 | 31.524 | 4.87E-05 | uncharacterized(LOC102603306) | mRNA |
| 41 | 102579962 | -2.82 | 4.358 | 31.528 | 4.89E-05 | homeobox-leucine_zipper_protein_ATHB-12-like(LOC102579962) | mRNA |
| 42 | 102585652 | -3.103 | 1.356 | 31.3 | 5.08E-05 | uncharacterized(LOC102585652) | mRNA |
| 43 | 102606436 | -6.529 | 6.795 | 31.242 | 5.13E-05 | 17.6_kDa_class_I_heat_shock_protein(LOC102606436) | mRNA |
| 44 | 102604442 | -2.421 | 2.478 | 30.656 | 5.66E-05 | DUF21_domain-containing_protein_At4g14240-like(LOC102604442) | mRNA |
| 45 | 102603133 | -2.552 | 0.6 | 30.319 | 5.84E-05 | putative_pentatricopeptide_repeat-containing_protein_At5g13230_mitochondrial(LOC102603133) | mRNA |
| 46 | 102579854 | -3.243 | 1.788 | 30.37 | 5.94E-05 | protein_NRT1/_PTR_FAMILY_2.8(LOC102579854) | mRNA |
| 47 | 102582408 | -5.178 | 6.148 | 29.974 | 6.35E-05 | probable_protein_phosphatase_2C_51(LOC102582408) | mRNA |
| 48 | 102587681 | -2.073 | 4.228 | 29.722 | 6.64E-05 | glycine-rich_domain-containing_protein_2(LOC102587681) | mRNA |
| 49 | 102597049 | -2.299 | 0.9 | 29.566 | 6.66E-05 | aluminum-activated_malate_transporter_9-like(LOC102597049) | mRNA |
| 50 | 107059214 | -5.185 | 0.598 | 29.203 | 7.26E-05 | uncharacterized(LOC107059214) | mRNA |
| 51 | 102591696 | -2.771 | 4.789 | 29.179 | 7.29E-05 | uncharacterized(LOC102591696) | mRNA |
| 52 | 102587091 | -6.242 | 4.135 | 28.583 | 8.10E-05 | uncharacterized(LOC102587091) | ncRNA |
| 53 | 102597226 | -2.814 | 4.159 | 28.5 | 8.22E-05 | uncharacterized(LOC102597226) | mRNA |
| 54 | 102593087 | -3.494 | 1.307 | 28.319 | 8.49E-05 | protein_terminal_ear1-like(LOC102593087) | mRNA |
| 55 | 102601716 | -2.049 | 1.694 | 28.205 | 8.62E-05 | secoisolariciresinol_dehydrogenase-like(LOC102601716) | mRNA |
| 56 | 102603734 | -2.036 | 0.365 | 28.041 | 8.71E-05 | pentatricopeptide_repeat-containing_protein_At3g63370_chloroplastic(LOC102603734) | mRNA |
| 57 | 102601943 | -2.968 | 2.346 | 28.117 | 8.80E-05 | E3_ubiquitin-protein_ligase_RNF170-like(LOC102601943) | mRNA |
| 58 | 102587642 | -3.709 | 3.214 | 28.067 | 8.88E-05 | nuclear_transcription_factor_Y_subunit_A-10-like(LOC102587642) | mRNA |
| 59 | 102606179 | -6.703 | 6.801 | 27.876 | 9.19E-05 | 18.2_kDa_class_I_heat_shock_protein-like(LOC102606179) | mRNA |
| 60 | 102596945 | -5.75 | 5.587 | 27.77 | 9.37E-05 | uncharacterized(LOC102596945) | mRNA |
| 61 | 102604427 | -2.757 | 5.794 | 26.66 | 1.15E-04 | zinc_finger_CCCH_domain-containing_protein_20(LOC102604427) | mRNA |
| 62 | 102591072 | -2.813 | 4.252 | 26.607 | 1.16E-04 | phosphoenolpyruvate_carboxylase_kinase_2-like(LOC102591072) | mRNA |
| 63 | 102589744 | -3.81 | 6.82 | 26.533 | 1.18E-04 | methionine_gamma-lyase-like(LOC102589744) | mRNA |
| 64 | 102600114 | -6.245 | 5.305 | 26.037 | 1.29E-04 | cell_division_cycle_protein_48_homolog(LOC102600114) | mRNA |
| 65 | 102594560 | -2.324 | 1.539 | 25.74 | 1.37E-04 | kirola-like(LOC102594560) | mRNA |
| 66 | 102606273 | -2.885 | 6.275 | 25.689 | 1.38E-04 | extensin-1-like(LOC102606273) | mRNA |
| 67 | 102589300 | -3.901 | 4.163 | 25.539 | 1.42E-04 | alanine--glyoxylate_aminotransferase_2_homolog_2_mitochondrial(LOC102589300) | mRNA |
| 68 | 102601966 | -2.288 | 4.963 | 25.335 | 1.48E-04 | sporulation-specific_protein_12-like(LOC102601966) | mRNA |
| 69 | 102597754 | -3.46 | 1.078 | 25.291 | 1.49E-04 | uncharacterized(LOC102597754) | ncRNA |
| 70 | 102578188 | -5.304 | 7.275 | 25.026 | 1.57E-04 | 18.2_kDa_class_I_heat_shock_protein-like(LOC102578188) | mRNA |
| 71 | 102585807 | -2.498 | 1.518 | 24.944 | 1.59E-04 | subtilisin-like_protease_SBT1.7(LOC102585807) | mRNA |
| 72 | 102594880 | -2.044 | 2.824 | 24.834 | 1.63E-04 | GATA_zinc_finger_domain-containing_protein_14-like(LOC102594880) | mRNA |
| 73 | 102579702 | -3.911 | 0.161 | 24.651 | 1.67E-04 | ferredoxin_root_R-B2-like(LOC102579702) | mRNA |
| 74 | 102581348 | -2.186 | 1.289 | 24.578 | 1.70E-04 | glutaredoxin-C11(LOC102581348) | mRNA |
| 75 | 102600872 | -2.308 | 2.432 | 24.327 | 1.80E-04 | WAT1-related_protein_At1g09380-like(LOC102600872) | mRNA |
| 76 | 102580883 | -3.529 | 3.118 | 24.007 | 1.91E-04 | EID1-like_F-box_protein_3(LOC102580883) | mRNA |
| 77 | 102605916 | -2.128 | 4.923 | 23.932 | 1.94E-04 | glycine-rich_cell_wall_structural_protein_2-like(LOC102605916) | mRNA |
| 78 | 102599705 | -3.752 | 5.12 | 23.89 | 1.96E-04 | non-specific_lipid-transfer_protein_4-like(LOC102599705) | mRNA |
| 79 | 102584601 | -3.292 | 3.594 | 23.812 | 1.99E-04 | heat_shock_70_kDa_protein-like(LOC102584601) | mRNA |
| 80 | 102590433 | -5.665 | 4.743 | 23.784 | 2.00E-04 | uncharacterized(LOC102590433) | mRNA |
| 81 | 102601835 | -2.65 | 4.713 | 23.679 | 2.04E-04 | probable_protein_phosphatase_2C_24(LOC102601835) | mRNA |
| 82 | 102601494 | -5.551 | 5.781 | 23.651 | 2.06E-04 | 22.7_kDa_class_IV_heat_shock_protein-like(LOC102601494) | mRNA |
| 83 | 102594541 | -3.512 | 8.305 | 23.621 | 2.07E-04 | delta-1-pyrroline-5-carboxylate_synthase-like(LOC102594541) | mRNA |
| 84 | 102588746 | -4.823 | 6.052 | 23.468 | 2.13E-04 | 18.2_kDa_class_I_heat_shock_protein-like(LOC102588746) | mRNA |
| 85 | 102606049 | -7.901 | 5.395 | 23.454 | 2.14E-04 | fidgetin-like_protein_1(LOC102606049) | mRNA |
| 86 | 102598218 | -6.4 | 5.269 | 23.365 | 2.18E-04 | translocator_protein_homolog(LOC102598218) | mRNA |
| 87 | 102595102 | -2.21 | 0.934 | 23.262 | 2.20E-04 | uncharacterized(LOC102595102) | mRNA |
| 88 | 102588418 | -7.039 | 7.076 | 23.303 | 2.20E-04 | heat_shock_70_kDa_protein_5(LOC102588418) | mRNA |
| 89 | 102597686 | -3.177 | 4.048 | 23.186 | 2.26E-04 | late_embryogenesis_abundant_protein-like(LOC102597686) | mRNA |
| 90 | 102591190 | -4.95 | 4.028 | 23.185 | 2.26E-04 | 17.4_kDa_class_III_heat_shock_protein(LOC102591190) | mRNA |
| 91 | 102595026 | -2.081 | 1.114 | 22.985 | 2.33E-04 | uncharacterized(LOC102595026) | mRNA |
| 92 | 102585726 | -3.401 | 3.506 | 22.988 | 2.35E-04 | homeobox-leucine_zipper_protein_ATHB-7-like(LOC102585726) | mRNA |
| 93 | 102601737 | -8.971 | 3.963 | 22.979 | 2.36E-04 | probable_sodium-coupled_neutral_amino_acid_transporter_6(LOC102601737) | mRNA |
| 94 | 102600485 | -2.426 | 1.315 | 22.788 | 2.45E-04 | protein_LIGHT-DEPENDENT_SHORT_HYPOCOTYLS_10-like(LOC102600485) | mRNA |
| 95 | 102605134 | -5.126 | 2.459 | 22.765 | 2.46E-04 | gibberellin_2-beta-dioxygenase_1-like(LOC102605134) | mRNA |
| 96 | 102600264 | -2.035 | 6.276 | 22.752 | 2.47E-04 | F-box/kelch-repeat_protein_At1g15670-like(LOC102600264) | mRNA |
| 97 | 102582606 | -2.456 | 6.332 | 22.714 | 2.49E-04 | transcription_factor_bHLH122-like(LOC102582606) | mRNA |
| 98 | 102579007 | -2.401 | 6.414 | 22.705 | 2.49E-04 | protein_ECERIFERUM_1-like(LOC102579007) | mRNA |
| 99 | 102583475 | -3.723 | 4.973 | 22.695 | 2.50E-04 | heat_shock_70_kDa_protein_8(LOC102583475) | mRNA |
| 100 | 102585136 | -6.09 | 3.439 | 22.68 | 2.50E-04 | non-specific_lipid-transfer_protein_2-like(LOC102585136) | mRNA |
| 101 | 102587639 | -6.539 | 0.221 | 22.672 | 2.51E-04 | 18.1_kDa_class_I_heat_shock_protein-like(LOC102587639) | mRNA |
| 102 | 102599929 | -3.169 | 0.951 | 22.654 | 2.52E-04 | RING-H2_finger_protein_ATL78-like(LOC102599929) | mRNA |
| 103 | 102577501 | -3.279 | 8.615 | 22.579 | 2.56E-04 | non-specific_lipid_transfer_protein_a7(LOC102577501) | mRNA |
| 104 | 102585909 | -3.253 | 1.519 | 22.525 | 2.59E-04 | basic_leucine_zipper_63(LOC102585909) | mRNA |
| 105 | 107059183 | -2.494 | 1.628 | 22.503 | 2.60E-04 | uncharacterized(LOC107059183) | ncRNA |
| 106 | 107057685 | -8.936 | 11.128 | 22.116 | 2.82E-04 | abscisic_acid_and_environmental_stress-inducible_protein_TAS14-like(LOC107057685) | mRNA |
| 107 | 102591828 | -4.91 | 4.125 | 22.113 | 2.82E-04 | heat_shock_factor_protein_HSF30-like(LOC102591828) | mRNA |
| 108 | 102578442 | -7.029 | 6.11 | 21.992 | 2.89E-04 | 17.7_kDa_class_I_heat_shock_protein-like(LOC102578442) | mRNA |
| 109 | 107062635 | -3.811 | 1.561 | 21.944 | 2.92E-04 | uncharacterized(LOC107062635) | ncRNA |
| 110 | 102593474 | -2.552 | 7.395 | 21.942 | 2.92E-04 | thioredoxin-like_1-1_chloroplastic(LOC102593474) | mRNA |
| 111 | 102597309 | -3.349 | 6.972 | 21.907 | 2.94E-04 | non-specific_lipid-transfer_protein_2-like(LOC102597309) | mRNA |
| 112 | 102578969 | -8.274 | 4.152 | 21.841 | 2.98E-04 | 26.5_kDa_heat_shock_protein_mitochondrial(LOC102578969) | mRNA |
| 113 | 102577575 | -4.282 | 6.223 | 21.822 | 3.00E-04 | sucrose_synthase(LOC102577575) | mRNA |
| 114 | 102596667 | -7.093 | 5.895 | 21.788 | 3.02E-04 | low-temperature-induced_65_kDa_protein-like(LOC102596667) | mRNA |
| 115 | 102577461 | -2.909 | 6.962 | 21.683 | 3.09E-04 | ABRE_binding_factor(LOC102577461) | mRNA |
| 116 | 102589092 | -6.156 | 5.086 | 21.654 | 3.11E-04 | homeobox-leucine_zipper_protein_ATHB-7-like(LOC102589092) | mRNA |
| 117 | 102599550 | -8.861 | 3.867 | 21.646 | 3.11E-04 | expansin-like_B1(LOC102599550) | mRNA |
| 118 | 107059622 | -4.063 | 0.149 | 21.608 | 3.13E-04 | uncharacterized(LOC107059622) | ncRNA |
| 119 | 102598989 | -2.605 | 1.513 | 21.495 | 3.21E-04 | cysteine_proteinase_inhibitor_B(LOC102598989) | mRNA |
| 120 | 102591596 | -7.314 | 0.923 | 21.386 | 3.29E-04 | uncharacterized(LOC102591596) | mRNA |
| 121 | 102591877 | -4.196 | 5.997 | 21.289 | 3.36E-04 | delta-amyrin_synthase(LOC102591877) | mRNA |
| 122 | 102599348 | -5.59 | 5.691 | 21.256 | 3.38E-04 | protein_phosphatase_2C_37-like(LOC102599348) | mRNA |
| 123 | 102597029 | -2.028 | 4.75 | 21.231 | 3.40E-04 | transcriptional_activator_TAF-1-like(LOC102597029) | mRNA |
| 124 | 102602655 | -4.651 | 2.599 | 21.186 | 3.43E-04 | uncharacterized(LOC102602655) | mRNA |
| 125 | 102588485 | -3.297 | 2.641 | 21.174 | 3.44E-04 | CASP-like_protein_2B1(LOC102588485) | mRNA |
| 126 | 102598982 | -2.029 | 0.397 | 20.813 | 3.66E-04 | dof_zinc_finger_protein_DOF3.4(LOC102598982) | mRNA |
| 127 | 102589078 | -4.865 | 5.606 | 20.771 | 3.76E-04 | 17.4_kDa_class_I_heat_shock_protein-like(LOC102589078) | mRNA |
| 128 | 102593309 | -7.985 | 2.667 | 20.735 | 3.79E-04 | protein_BIG_GRAIN_1-like_B(LOC102593309) | mRNA |
| 129 | 102598306 | -5.215 | 4.04 | 20.654 | 3.85E-04 | SNF1-related_protein_kinase_regulatory_subunit_gamma-like_PV42a(LOC102598306) | mRNA |
| 130 | 102597777 | -4.837 | 4.006 | 20.632 | 3.87E-04 | uncharacterized(LOC102597777) | mRNA |
| 131 | 102594557 | -2.958 | 6.181 | 20.617 | 3.89E-04 | ninja-family_protein_AFP3(LOC102594557) | mRNA |
| 132 | 102581483 | -5.106 | 2.229 | 20.585 | 3.91E-04 | heavy_metal-associated_isoprenylated_plant_protein_26-like(LOC102581483) | mRNA |
| 133 | 102598452 | -5.786 | 5.641 | 20.584 | 3.91E-04 | auxin-repressed_12.5_kDa_protein-like(LOC102598452) | mRNA |
| 134 | 102577597 | -3.616 | 8.498 | 20.492 | 3.99E-04 | chaperone_protein_ClpB1(LOC102577597) | mRNA |
| 135 | 102601370 | -3.862 | 5.289 | 20.481 | 4.00E-04 | uncharacterized(LOC102601370) | mRNA |
| 136 | 102593843 | -2.235 | 0.805 | 20.401 | 4.04E-04 | pentatricopeptide_repeat-containing_protein_At5g08305(LOC102593843) | mRNA |
| 137 | 102595370 | -2.136 | 4.599 | 20.36 | 4.11E-04 | basic_7S_globulin-like(LOC102595370) | mRNA |
| 138 | 107058400 | -4.414 | 0.934 | 20.294 | 4.17E-04 | calsequestrin-1-like(LOC107058400) | mRNA |
| 139 | 102591434 | -7.483 | 4.667 | 20.124 | 4.33E-04 | uncharacterized(LOC102591434) | mRNA |
| 140 | 102598583 | -3.706 | 5.078 | 19.989 | 4.46E-04 | non-specific_lipid-transfer_protein_2-like(LOC102598583) | mRNA |
| 141 | 107060345 | -3.002 | 0.428 | 19.911 | 4.52E-04 | pentatricopeptide_repeat-containing_protein_At5g66520-like(LOC107060345) | mRNA |
| 142 | 102603811 | -5.771 | 6.72 | 19.628 | 4.84E-04 | ATP-dependent_zinc_metalloprotease_FTSH_6_chloroplastic(LOC102603811) | mRNA |
| 143 | 107062454 | -2.303 | 0.72 | 19.454 | 5.00E-04 | uncharacterized(LOC107062454) | mRNA |
| 144 | 102584349 | -2.072 | 0.838 | 19.4 | 5.05E-04 | uncharacterized(LOC102584349) | ncRNA |
| 145 | 102589273 | -3.457 | 4.031 | 19.429 | 5.06E-04 | PI-PLC_X-box_domain-containing_protein_DDB_G0293730-like(LOC102589273) | mRNA |
| 146 | 102605148 | -6.45 | 2.889 | 19.348 | 5.16E-04 | protein_LE25-like(LOC102605148) | mRNA |
| 147 | 107059770 | -2.66 | 2.701 | 19.348 | 5.16E-04 | uncharacterized(LOC107059770) | ncRNA |
| 148 | 102584024 | -2.138 | 5.831 | 19.334 | 5.18E-04 | probable_non-specific_lipid-transfer_protein_AKCS9-like(LOC102584024) | mRNA |
| 149 | 102602727 | -2.371 | 1.259 | 19.146 | 5.40E-04 | pentatricopeptide_repeat-containing_protein_At2g27610(LOC102602727) | mRNA |
| 150 | 102581810 | -5.614 | 6.706 | 19.128 | 5.42E-04 | heat_shock_protein_83-like(LOC102581810) | misc_RNA |
| 151 | 102588742 | -2.157 | 3.788 | 19.056 | 5.52E-04 | uncharacterized(LOC102588742) | ncRNA |
| 152 | 102605762 | -6.556 | 5.944 | 19.039 | 5.54E-04 | 17.7_kDa_class_I_heat_shock_protein-like(LOC102605762) | mRNA |
| 153 | 102595164 | -2.426 | 5.418 | 18.652 | 6.06E-04 | uncharacterized(LOC102595164) | mRNA |
| 154 | 102588710 | -6.677 | 5.031 | 18.635 | 6.08E-04 | non-specific_lipid-transfer_protein_2-like(LOC102588710) | mRNA |
| 155 | 102601122 | -4.119 | 3.775 | 18.632 | 6.08E-04 | calcium_uniporter_protein_2_mitochondrial(LOC102601122) | mRNA |
| 156 | 102594103 | -4.75 | 5.288 | 18.632 | 6.09E-04 | 15.7_kDa_heat_shock_protein_peroxisomal(LOC102594103) | mRNA |
| 157 | 102592278 | -5.866 | 2.818 | 18.614 | 6.11E-04 | expansin-like_B1(LOC102592278) | mRNA |
| 158 | 102600070 | -2.461 | 2.272 | 18.594 | 6.14E-04 | uncharacterized(LOC102600070) | mRNA |
| 159 | 102593413 | -2.081 | 2.186 | 18.528 | 6.23E-04 | uncharacterized(LOC102593413) | ncRNA |
| 160 | 102584557 | -5.357 | 2.726 | 18.469 | 6.32E-04 | uncharacterized(LOC102584557) | mRNA |
| 161 | 102581388 | -4.361 | 4.295 | 18.464 | 6.33E-04 | glutamate_decarboxylase(LOC102581388) | mRNA |
| 162 | 102582967 | -5.427 | 2.603 | 18.448 | 6.35E-04 | class_I_heat_shock_protein-like(LOC102582967) | mRNA |
| 163 | 102588806 | -2.262 | 3.291 | 18.441 | 6.36E-04 | phosphoglucan_phosphatase_DSP4_amyloplastic-like(LOC102588806) | mRNA |
| 164 | 102584526 | -3.213 | 4.339 | 18.436 | 6.37E-04 | uncharacterized(LOC102584526) | ncRNA |
| 165 | 102588345 | -2.1 | 1.4 | 18.432 | 6.38E-04 | 11-beta-hydroxysteroid_dehydrogenase_1B-like(LOC102588345) | mRNA |
| 166 | 102599989 | -2.107 | 3.1 | 18.423 | 6.39E-04 | protein_NRT1/_PTR_FAMILY_1.2(LOC102599989) | mRNA |
| 167 | 102594742 | -2.343 | 4.427 | 18.358 | 6.49E-04 | uncharacterized(LOC102594742) | mRNA |
| 168 | 107060836 | -6.812 | 0.488 | 18.346 | 6.51E-04 | heavy_metal-associated_isoprenylated_plant_protein_26-like(LOC107060836) | mRNA |
| 169 | 102579798 | -2.14 | 4.321 | 18.293 | 6.59E-04 | homogentisate_12-dioxygenase(LOC102579798) | mRNA |
| 170 | 102578786 | -6.914 | 7.419 | 18.211 | 6.72E-04 | 17.6_kDa_class_I_heat_shock_protein-like(LOC102578786) | mRNA |
| 171 | 102603559 | -3.506 | 3.917 | 18.182 | 6.76E-04 | uncharacterized_protein_OsI_027940-like(LOC102603559) | mRNA |
| 172 | 102584616 | -5.887 | 2.919 | 18.178 | 6.77E-04 | uncharacterized(LOC102584616) | mRNA |
| 173 | 102597445 | -2.449 | 6.791 | 18.128 | 6.85E-04 | chaperone_protein_ClpB4_mitochondrial-like(LOC102597445) | mRNA |
| 174 | 102598867 | -4.292 | 6.914 | 18.066 | 6.95E-04 | small_heat_shock_protein_chloroplastic-like(LOC102598867) | mRNA |
| 175 | 102584371 | -7.21 | 4.703 | 18.027 | 7.02E-04 | 22.7_kDa_class_IV_heat_shock_protein-like(LOC102584371) | mRNA |
| 176 | 102585181 | -4.098 | 1.609 | 17.96 | 7.13E-04 | inorganic_pyrophosphatase_1-like(LOC102585181) | mRNA |
| 177 | 102604056 | -2.487 | 4.676 | 17.951 | 7.14E-04 | cytochrome_P450_83B1-like(LOC102604056) | mRNA |
| 178 | 102597176 | -2.329 | 1.115 | 17.919 | 7.20E-04 | ATP-dependent_helicase_rhp16-like(LOC102597176) | mRNA |
| 179 | 102584503 | -8.459 | 6.778 | 17.891 | 7.25E-04 | small_heat_shock_protein_chloroplastic(LOC102584503) | mRNA |
| 180 | 102591853 | -6.949 | 7.466 | 17.822 | 7.37E-04 | small_heat_shock_protein_chloroplastic-like(LOC102591853) | mRNA |
| 181 | 107060976 | -2.424 | 2.235 | 17.812 | 7.39E-04 | uncharacterized(LOC107060976) | ncRNA |
| 182 | 102589363 | -6.035 | 4.743 | 17.797 | 7.41E-04 | non-specific_lipid-transfer_protein_2-like(LOC102589363) | mRNA |
| 183 | 102595571 | -4.945 | 5.218 | 17.755 | 7.49E-04 | cysteine_protease_inhibitor_1(LOC102595571) | mRNA |
| 184 | 102586496 | -2.17 | 6.916 | 17.706 | 7.57E-04 | cell_wall_/_vacuolar_inhibitor_of_fructosidase_1-like(LOC102586496) | mRNA |
| 185 | 102581644 | -3.314 | 1.708 | 17.695 | 7.59E-04 | poly_[ADP-ribose]_polymerase_3(LOC102581644) | mRNA |
| 186 | 102577712 | -3.498 | 6.782 | 17.649 | 7.68E-04 | sucrose_synthase_2(LOC102577712) | mRNA |
| 187 | 102586146 | -2.121 | 2.109 | 17.548 | 7.87E-04 | glutamyl-tRNA(Gln)_amidotransferase_subunit_A-like(LOC102586146) | mRNA |
| 188 | 102600790 | -2.92 | 2.884 | 17.489 | 7.98E-04 | E3_ubiquitin-protein_ligase_RMA1H1-like(LOC102600790) | mRNA |
| 189 | 102596252 | -2.206 | 7.39 | 17.42 | 8.12E-04 | protein_phosphatase_2C_37-like(LOC102596252) | mRNA |
| 190 | 102580640 | -5.045 | 0.946 | 17.338 | 8.28E-04 | heavy_metal-associated_isoprenylated_plant_protein_26-like(LOC102580640) | mRNA |
| 191 | 102603244 | -3.164 | 6.508 | 17.312 | 8.33E-04 | heat_shock_protein_83(LOC102603244) | mRNA |
| 192 | 102602565 | -5.606 | 2.478 | 17.229 | 8.50E-04 | low-temperature-induced_78_kDa_protein-like(LOC102602565) | mRNA |
| 193 | 102584365 | -3.772 | 4.275 | 17.212 | 8.54E-04 | uncharacterized(LOC102584365) | mRNA |
| 194 | 107061200 | -2.325 | 3.103 | 17.205 | 8.55E-04 | uncharacterized(LOC107061200) | ncRNA |
| 195 | 102589396 | -3.865 | 5.437 | 17.102 | 8.77E-04 | 17.4_kDa_class_I_heat_shock_protein-like(LOC102589396) | mRNA |
| 196 | 107059433 | -4.564 | 0.228 | 17 | 8.99E-04 | uncharacterized(LOC107059433) | ncRNA |
| 197 | 102606249 | -3.078 | 5.824 | 16.97 | 9.06E-04 | dnaJ_protein_homolog(LOC102606249) | mRNA |
| 198 | 102578720 | -4.422 | 6.981 | 16.941 | 9.12E-04 | L-ascorbate_peroxidase_cytosolic(LOC102578720) | mRNA |
| 199 | 102598100 | -3.166 | 0.6 | 16.902 | 9.21E-04 | 17.6_kDa_class_I_heat_shock_protein-like(LOC102598100) | mRNA |
| 200 | 102600802 | -2.162 | 3.989 | 16.802 | 9.44E-04 | BTB/POZ_domain-containing_protein_At3g22104-like(LOC102600802) | mRNA |
| 201 | 102577576 | -4.998 | 7.459 | 16.799 | 9.45E-04 | asparagine_synthetase_[glutamine-hydrolyzing](LOC102577576) | mRNA |
| 202 | 102598584 | -2.314 | 0.959 | 16.781 | 9.48E-04 | ABC_transporter_G_family_member_4-like(LOC102598584) | mRNA |
| 203 | 102594513 | -4.902 | 1.677 | 16.749 | 9.57E-04 | uncharacterized(LOC102594513) | mRNA |
| 204 | 102599238 | -3.701 | 0.816 | 16.744 | 9.58E-04 | (EE)-geranyllinalool_synthase(LOC102599238) | mRNA |
| 205 | 102586866 | -5.773 | 0.855 | 16.705 | 9.67E-04 | uncharacterized(LOC102586866) | mRNA |
| 206 | 102580120 | -6.602 | 7.935 | 16.693 | 9.70E-04 | expansin-like_B1(LOC102580120) | mRNA |
| 207 | 102590098 | -2.325 | 4.922 | 16.654 | 9.80E-04 | tetraketide_alpha-pyrone_reductase_1-like(LOC102590098) | mRNA |
| 208 | 102605425 | -5.033 | 9.462 | 16.605 | 9.92E-04 | 17.8_kDa_class_I_heat_shock_protein(LOC102605425) | mRNA |

**C. Upregulated genes between ATLWT (Mock) and ATLWT(PVY-inoculated)**

| Rank | GeneID | LogFC | LogCPM | F | P_value | Annotation | RNAtype |
| --- | --- | --- | --- | --- | --- | --- | --- |
|  |  |  |  |  |  |  |  |
| **1** | **PVY000000** | **10.067** | **16.17** | **1469.306** | **1.74E-25** | **potato_virus_Y** | **virus** |
| 2 | 102592415 | 2.678 | 3.08 | 167.474 | 3.08E-13 | uncharacterized(LOC102592415) | mRNA |
| 3 | 102591245 | 2.605 | 7.389 | 160.247 | 5.32E-13 | protein_argonaute_2-like(LOC102591245) | mRNA |
| 4 | 102581338 | 3.405 | 2.344 | 140.03 | 2.56E-12 | probable_calcium-binding_protein_CML45(LOC102581338) | mRNA |
| 5 | 107060856 | 2.062 | 4.784 | 124.879 | 9.90E-12 | uncharacterized(LOC107060856) | mRNA |
| 6 | 102600347 | 2.113 | 4.26 | 115.971 | 2.29E-11 | F-box_protein_PP2-B10-like(LOC102600347) | mRNA |
| 7 | 102587895 | 2.047 | 2.95 | 114.972 | 2.38E-11 | receptor-like_protein_12(LOC102587895) | mRNA |
| 8 | 107063336 | 2.288 | 4.137 | 112.985 | 3.06E-11 | TMV_resistance_protein_N-like(LOC107063336) | mRNA |
| 9 | 102588802 | 4.307 | 1.751 | 110.779 | 3.76E-11 | zinc_finger_protein_ZAT11-like(LOC102588802) | mRNA |
| 10 | 102579771 | 2.582 | 6.342 | 103.738 | 7.87E-11 | mitochondrial_uncoupling_protein_5-like(LOC102579771) | mRNA |
| 11 | 102604255 | 4.353 | 4.596 | 97.558 | 1.54E-10 | coatomer_subunit_beta'-1-like(LOC102604255) | mRNA |
| 12 | 102589233 | 2.557 | 1.633 | 95.439 | 1.86E-10 | LRR_receptor-like_serine/threonine-protein_kinase_ERL1(LOC102589233) | mRNA |
| 13 | 102602021 | 2.51 | 4.663 | 95.732 | 1.88E-10 | zinc_finger_A20_and_AN1_domain-containing_stress-associated_protein_1(LOC102602021) | mRNA |
| 14 | 102589114 | 2.484 | 3.151 | 95.587 | 1.91E-10 | ubiquinol_oxidase_2_mitochondrial-like(LOC102589114) | mRNA |
| 15 | 102601376 | 2.454 | 3.781 | 94.083 | 2.27E-10 | outer_envelope_membrane_protein_7-like(LOC102601376) | mRNA |
| 16 | 102599106 | 2.039 | 6.407 | 91.982 | 2.89E-10 | thioredoxin_H-type_2-like(LOC102599106) | mRNA |
| 17 | 107063333 | 2.016 | 4.114 | 89.565 | 3.84E-10 | putative_disease_resistance_protein_At4g11170(LOC107063333) | mRNA |
| 18 | 102592778 | 3.452 | 1.884 | 88.202 | 4.47E-10 | probable_calcium-binding_protein_CML44(LOC102592778) | mRNA |
| 19 | 102594317 | 2.281 | 2.301 | 86.19 | 5.53E-10 | pirin-like_protein_At1g50590(LOC102594317) | mRNA |
| 20 | 102604739 | 2.588 | 3.865 | 85.042 | 6.64E-10 | uncharacterized(LOC102604739) | mRNA |
| 21 | 102592249 | 3.108 | 2.029 | 81.906 | 9.73E-10 | uncharacterized(LOC102592249) | ncRNA |
| 22 | 107060721 | 2.837 | 2.264 | 79.631 | 1.31E-09 | uncharacterized_N-acetyltransferase_p20-like(LOC107060721) | mRNA |
| 23 | 102605066 | 2.312 | 6.027 | 76.569 | 1.97E-09 | NAC_domain-containing_protein_91(LOC102605066) | mRNA |
| 24 | 102602279 | 2.567 | 3.737 | 72.433 | 3.47E-09 | probable_WRKY_transcription_factor_51(LOC102602279) | mRNA |
| 25 | 107061001 | 5.575 | 1.602 | 72.284 | 3.54E-09 | protein_RADIALIS-like_1(LOC107061001) | mRNA |
| 26 | 102577619 | 2.082 | 4.12 | 72.047 | 3.66E-09 | 1-aminocyclopropane-1-carboxylate_synthase(STACS5) | mRNA |
| 27 | 102591126 | 2.056 | 3.051 | 71.714 | 3.81E-09 | uncharacterized(LOC102591126) | mRNA |
| 28 | 107060694 | 3.223 | 3.697 | 70.145 | 4.78E-09 | sigma_factor_binding_protein_2_chloroplastic-like(LOC107060694) | mRNA |
| 29 | 107057797 | 3.145 | 2.368 | 69.024 | 5.62E-09 | uncharacterized(LOC107057797) | ncRNA |
| 30 | 102582861 | 2.259 | 2.628 | 68.895 | 5.69E-09 | receptor-like_protein_12(LOC102582861) | mRNA |
| 31 | 102598548 | 3.409 | 2.249 | 68.515 | 6.05E-09 | AAA-ATPase_At3g28600-like(LOC102598548) | mRNA |
| 32 | 102595394 | 2.955 | 0.955 | 67.312 | 6.95E-09 | crocetin_glucosyltransferase_chloroplastic-like(LOC102595394) | mRNA |
| 33 | 102580435 | 2.582 | 3.544 | 67.276 | 7.25E-09 | receptor-like_protein_12(LOC102580435) | misc_RNA |
| 34 | 102603333 | 2.651 | 3.879 | 64.652 | 1.07E-08 | cytochrome_P450_71D11-like(LOC102603333) | mRNA |
| 35 | 107058370 | 2.967 | 1.321 | 64.064 | 1.14E-08 | probable_calcium-binding_protein_CML30(LOC107058370) | mRNA |
| 36 | 102592079 | 2.148 | 4.019 | 63.807 | 1.22E-08 | probable_inactive_leucine-rich_repeat_receptor-like_protein_kinase_At1g66830(LOC102592079) | mRNA |
| 37 | 107058598 | 4.94 | 0.89 | 63.714 | 1.23E-08 | cinnamate_beta-D-glucosyltransferase-like(LOC107058598) | mRNA |
| 38 | 107059057 | 2.461 | 2.36 | 63.506 | 1.27E-08 | receptor-like_protein_12(LOC107059057) | mRNA |
| 39 | 102586681 | 3.673 | 4.902 | 62.377 | 1.52E-08 | calcium-binding_protein_PBP1-like(LOC102586681) | mRNA |
| 40 | 102577728 | 2.968 | 8.183 | 61.914 | 1.63E-08 | NADH_nitrate_reductase(NR3) | mRNA |
| 41 | 102589598 | 3.381 | 0.944 | 61.481 | 1.69E-08 | mitogen-activated_protein_kinase_kinase_kinase_NPK1-like(LOC102589598) | mRNA |
| 42 | 102581437 | 2.173 | 2.646 | 59.824 | 2.27E-08 | probable_leucine-rich_repeat_receptor-like_protein_kinase_At5g63930(LOC102581437) | mRNA |
| 43 | 102597396 | 2.348 | 0.994 | 59.511 | 2.31E-08 | ankyrin_repeat-containing_protein_At3g12360-like(LOC102597396) | mRNA |
| 44 | 107059312 | 2.699 | 3.992 | 58.27 | 2.92E-08 | uncharacterized(LOC107059312) | ncRNA |
| 45 | 102581157 | 2.036 | 2.055 | 57.698 | 3.12E-08 | probable_inorganic_phosphate_transporter_1-9(LOC102581157) | mRNA |
| 46 | 102589935 | 2.333 | 1.158 | 57.469 | 3.22E-08 | mitogen-activated_protein_kinase_kinase_kinase_NPK1-like(LOC102589935) | mRNA |
| 47 | 102592495 | 3.004 | 0.905 | 57.375 | 3.27E-08 | IQ_domain-containing_protein_IQM4-like(LOC102592495) | mRNA |
| 48 | 102589109 | 2.235 | 5.085 | 57.279 | 3.43E-08 | programmed_cell_death_6-interacting_protein(LOC102589109) | mRNA |
| 49 | 107062792 | 2.949 | 5.05 | 56.321 | 4.02E-08 | ethylene-responsive_transcription_factor_5-like(LOC107062792) | mRNA |
| 50 | 102595716 | 2.829 | 4.953 | 55.605 | 4.53E-08 | probable_E3_ubiquitin-protein_ligase_RNF217(LOC102595716) | mRNA |
| 51 | 107061032 | 2.422 | 1.722 | 55.231 | 4.74E-08 | uncharacterized(LOC107061032) | mRNA |
| 52 | 102586119 | 3.524 | 3.081 | 55.243 | 4.82E-08 | calcium-binding_protein_PBP1-like(LOC102586119) | mRNA |
| 53 | 107063164 | 4.487 | 1.066 | 54.259 | 5.69E-08 | zinc_finger_protein_ZAT10-like(LOC107063164) | mRNA |
| 54 | 102605080 | 2.144 | 3.306 | 54.185 | 5.76E-08 | uncharacterized(LOC102605080) | mRNA |
| 55 | 102605179 | 2.155 | 4.368 | 53.631 | 6.34E-08 | probable_WRKY_transcription_factor_30(LOC102605179) | mRNA |
| 56 | 102582261 | 2.306 | 3.011 | 53.285 | 6.73E-08 | probable_LRR_receptor-like_serine/threonine-protein_kinase_At3g47570(LOC102582261) | mRNA |
| 57 | 107058946 | 2.091 | 2.113 | 52.223 | 7.98E-08 | probable_LRR_receptor-like_serine/threonine-protein_kinase_At3g47570(LOC107058946) | mRNA |
| 58 | 102598791 | 2.186 | 1.581 | 52.129 | 8.02E-08 | NAC_domain-containing_protein_90-like(LOC102598791) | mRNA |
| 59 | 102604313 | 3.156 | 1.639 | 51.875 | 8.61E-08 | protein_SAR_DEFICIENT_1-like(LOC102604313) | mRNA |
| 60 | 102596739 | 2.187 | 4.483 | 51.688 | 8.90E-08 | protein_SRC2_homolog(LOC102596739) | mRNA |
| 61 | 102596181 | 2.587 | 0.906 | 51.303 | 9.27E-08 | uncharacterized(LOC102596181) | mRNA |
| 62 | 102587593 | 2.278 | 1.973 | 50.957 | 1.00E-07 | probable_LRR_receptor-like_serine/threonine-protein_kinase_At4g08850(LOC102587593) | mRNA |
| 63 | 102599976 | 3.391 | 4.403 | 49.9 | 1.22E-07 | ethylene-responsive_transcription_factor_5-like(LOC102599976) | mRNA |
| 64 | 102598154 | 2.364 | 2.452 | 49.476 | 1.32E-07 | non-functional_pseudokinase_ZED1-like(LOC102598154) | mRNA |
| 65 | 102587887 | 2.701 | 4.49 | 49.158 | 1.40E-07 | uncharacterized(LOC102587887) | mRNA |
| 66 | 102582648 | 2.148 | 5.286 | 48.762 | 1.51E-07 | B2_protein-like(LOC102582648) | mRNA |
| 67 | 102585187 | 2.94 | 5.46 | 48.029 | 1.73E-07 | ethylene-responsive_transcription_factor_5-like(LOC102585187) | mRNA |
| 68 | 107057655 | 3.923 | 3.083 | 47.054 | 2.07E-07 | probable_xyloglucan_endotransglucosylase/hydrolase_protein_23(LOC107057655) | mRNA |
| 69 | 102601867 | 4.839 | 0.297 | 46.972 | 2.07E-07 | putative_calcium-binding_protein_CML19(LOC102601867) | mRNA |
| 70 | 102593110 | 2.666 | 2.421 | 46.934 | 2.12E-07 | uncharacterized(LOC102593110) | mRNA |
| 71 | 102588726 | 2.087 | 0.903 | 46.672 | 2.17E-07 | proline_dehydrogenase_2_mitochondrial-like(LOC102588726) | mRNA |
| 72 | 102585109 | 2.41 | 5.012 | 46.662 | 2.23E-07 | protein_SAR_DEFICIENT_1-like(LOC102585109) | mRNA |
| 73 | 102589620 | 4.043 | 3.79 | 46.379 | 2.35E-07 | zinc_finger_protein_ZAT10-like(LOC102589620) | mRNA |
| 74 | 102601098 | 2.557 | 4.372 | 46.324 | 2.37E-07 | uncharacterized_N-acetyltransferase_p20-like(LOC102601098) | mRNA |
| 75 | 102586402 | 2.757 | 6.207 | 46.06 | 2.50E-07 | uncharacterized(LOC102586402) | mRNA |
| 76 | 102587899 | 3.485 | 5.404 | 45.818 | 2.62E-07 | ethylene-responsive_transcription_factor_5-like(LOC102587899) | mRNA |
| 77 | 102597894 | 2.044 | 2.76 | 45.498 | 2.78E-07 | VQ_motif-containing_protein_22-like(LOC102597894) | mRNA |
| 78 | 102599328 | 2.486 | 4.998 | 45.478 | 2.79E-07 | calcium-binding_allergen_Ole_e_8(LOC102599328) | mRNA |
| 79 | 102585525 | 3.573 | 2.362 | 45.255 | 2.91E-07 | ethylene-responsive_transcription_factor_5-like(LOC102585525) | mRNA |
| 80 | 102604232 | 2.71 | 3.944 | 45.191 | 2.95E-07 | probable_WRKY_transcription_factor_51(LOC102604232) | mRNA |
| 81 | 102600579 | 2.476 | 2.394 | 45.158 | 2.97E-07 | syntaxin-121-like(LOC102600579) | mRNA |
| 82 | 102591700 | 2.315 | 1.148 | 44.918 | 3.04E-07 | nuclear_transcription_factor_Y_subunit_B-4-like(LOC102591700) | mRNA |
| 83 | 102583367 | 5.109 | -0.142 | 44.883 | 3.06E-07 | zinc_finger_protein_ZAT11-like(LOC102583367) | mRNA |
| 84 | 102604988 | 3.05 | 2.311 | 45.002 | 3.06E-07 | 14_kDa_proline-rich_protein_DC2.15-like(LOC102604988) | mRNA |
| 85 | 102593417 | 2.097 | 3.638 | 44.415 | 3.43E-07 | acyl-protein_thioesterase_1_homolog_1-like(LOC102593417) | mRNA |
| 86 | 102587688 | 2.538 | 1.202 | 43.634 | 3.93E-07 | transcription_factor_RADIALIS(LOC102587688) | mRNA |
| 87 | 107062687 | 4.351 | 1.429 | 42.335 | 5.18E-07 | uncharacterized(LOC107062687) | mRNA |
| 88 | 102591257 | 2.142 | 3.878 | 42.331 | 5.18E-07 | uncharacterized_protein_At4g15970-like(LOC102591257) | mRNA |
| 89 | 102586342 | 2.436 | 3.126 | 42.044 | 5.49E-07 | E3_ubiquitin-protein_ligase_RHA2A-like(LOC102586342) | mRNA |
| 90 | 102586408 | 2.271 | 4.8 | 41.971 | 5.57E-07 | late_embryogenesis_abundant_protein_Lea5-like(LOC102586408) | mRNA |
| 91 | 102601336 | 2.66 | 1.37 | 41.714 | 5.84E-07 | probable_LRR_receptor-like_serine/threonine-protein_kinase_At3g47570(LOC102601336) | mRNA |
| 92 | 102590972 | 2.28 | 0.931 | 41.499 | 6.00E-07 | probable_linoleate_9S-lipoxygenase_5(LOC102590972) | mRNA |
| 93 | 107061092 | 2.039 | 3.588 | 41.552 | 6.06E-07 | uncharacterized(LOC107061092) | mRNA |
| 94 | 102601440 | 2.68 | 3.092 | 41.25 | 6.45E-07 | uncharacterized_N-acetyltransferase_p20-like(LOC102601440) | mRNA |
| 95 | 102595418 | 2.191 | 5.843 | 41.074 | 6.68E-07 | uncharacterized(LOC102595418) | ncRNA |
| 96 | 102584043 | 5.517 | 0.059 | 40.492 | 7.47E-07 | zinc_finger_protein_ZAT11-like(LOC102584043) | mRNA |
| 97 | 102579454 | 2.129 | 6.241 | 40.068 | 8.23E-07 | subtilisin-like_protease_SBT1.7(LOC102579454) | mRNA |
| 98 | 102584686 | 2.428 | 4.176 | 39.824 | 8.66E-07 | probable_pectinesterase/pectinesterase_inhibitor_12(LOC102584686) | mRNA |
| 99 | 102600044 | 2.031 | 2.058 | 39.03 | 1.02E-06 | protein_YLS9-like(LOC102600044) | mRNA |
| 100 | 102591415 | 3.258 | 4.97 | 39.034 | 1.02E-06 | RING-H2_finger_protein_ATL2-like(LOC102591415) | mRNA |
| 101 | 102585630 | 3.598 | 2.912 | 38.824 | 1.07E-06 | serpin-ZX-like(LOC102585630) | mRNA |
| 102 | 102585236 | 2.152 | 1.005 | 37.933 | 1.27E-06 | plant_intracellular_Ras-group-related_LRR_protein_6-like(LOC102585236) | mRNA |
| 103 | 102587227 | 3.041 | 5.287 | 37.976 | 1.28E-06 | prolyl_endopeptidase(LOC102587227) | mRNA |
| 104 | 102598120 | 2.268 | 3.496 | 37.215 | 1.51E-06 | type_I_inositol_polyphosphate_5-phosphatase_2-like(LOC102598120) | mRNA |
| 105 | 102580067 | 2.368 | 2.095 | 37.025 | 1.57E-06 | pathogenesis-related_genes_transcriptional_activator_PTI5(LOC102580067) | mRNA |
| 106 | 102601103 | 3.477 | 5.174 | 36.559 | 1.74E-06 | zinc_finger_protein_ZAT10(LOC102601103) | mRNA |
| 107 | 107059933 | 2.429 | 3.248 | 36.245 | 1.87E-06 | probably_inactive_leucine-rich_repeat_receptor-like_protein_kinase_At5g48380(LOC107059933) | mRNA |
| 108 | 102590788 | 2.333 | 2.256 | 35.913 | 2.01E-06 | F-box_protein_SKIP27-like(LOC102590788) | mRNA |
| 109 | 102603101 | 3.374 | 3.211 | 35.333 | 2.29E-06 | probable_galacturonosyltransferase-like_10(LOC102603101) | mRNA |
| 110 | 102594490 | 2.667 | 4.742 | 35.121 | 2.40E-06 | uncharacterized(LOC102594490) | mRNA |
| 111 | 102589957 | 4.07 | 0.543 | 34.781 | 2.59E-06 | zinc_finger_protein_ZAT10-like(LOC102589957) | mRNA |
| 112 | 102582772 | 2.049 | 8.056 | 34.375 | 2.84E-06 | zinc_finger_CCCH_domain-containing_protein_29-like(LOC102582772) | mRNA |
| 113 | 102585539 | 2.08 | 3.975 | 34.336 | 2.87E-06 | AAA-ATPase_At3g50940-like(LOC102585539) | mRNA |
| 114 | 102601927 | 3.524 | 2.01 | 34.119 | 3.01E-06 | L-ascorbate_oxidase-like(LOC102601927) | misc_RNA |
| 115 | 102597408 | 2.82 | 1.669 | 34.068 | 3.05E-06 | probable_inactive_receptor_kinase_RLK902(LOC102597408) | mRNA |
| 116 | 102592929 | 4.826 | 3.189 | 34.024 | 3.08E-06 | uncharacterized(LOC102592929) | mRNA |
| 117 | 102578270 | 3.517 | 5.102 | 34.011 | 3.09E-06 | probable_CCR4-associated_factor_1_homolog_9(LOC102578270) | mRNA |
| 118 | 107060673 | 2.672 | 0.733 | 33.863 | 3.15E-06 | auxin-responsive_protein_SAUR32-like(LOC107060673) | mRNA |
| 119 | 107059919 | 2.186 | 2.712 | 33.577 | 3.41E-06 | probably_inactive_leucine-rich_repeat_receptor-like_protein_kinase_At5g48380(LOC107059919) | mRNA |
| 120 | 102585479 | 2.576 | 3.088 | 33.413 | 3.54E-06 | uncharacterized(LOC102585479) | mRNA |
| 121 | 102584363 | 5.973 | -0.259 | 33.237 | 3.67E-06 | zinc_finger_protein_ZAT11-like(LOC102584363) | mRNA |
| 122 | 102578478 | 2.428 | 3.162 | 33.141 | 3.77E-06 | uncharacterized(LOC102578478) | mRNA |
| 123 | 102589401 | 3.087 | 0.209 | 33.041 | 3.80E-06 | putative_F-box_protein_PP2-B12(LOC102589401) | mRNA |
| 124 | 102598420 | 3.856 | 1.682 | 33.017 | 3.88E-06 | probable_xyloglucan_endotransglucosylase/hydrolase_protein_23(LOC102598420) | mRNA |
| 125 | 102583797 | 3.121 | 3.194 | 32.953 | 3.94E-06 | uncharacterized(LOC102583797) | mRNA |
| 126 | 102589987 | 2.589 | 2.053 | 32.645 | 4.24E-06 | uncharacterized(LOC102589987) | mRNA |
| 127 | 102605859 | 4.489 | 0.731 | 31.877 | 5.08E-06 | xyloglucan_endotransglucosylase/hydrolase_protein_15-like(LOC102605859) | mRNA |
| 128 | 102606196 | 4.489 | 0.731 | 31.877 | 5.08E-06 | xyloglucan_endotransglucosylase/hydrolase_protein_15-like(LOC102606196) | mRNA |
| 129 | 102600818 | 2.101 | 4.239 | 31.597 | 5.43E-06 | uncharacterized(LOC102600818) | mRNA |
| 130 | 102584914 | 3.352 | 1.707 | 31.575 | 5.46E-06 | calmodulin-binding_protein_60_A-like(LOC102584914) | mRNA |
| 131 | 102602350 | 3.802 | 0.112 | 31.431 | 5.59E-06 | zinc_finger_protein_ZAT10-like(LOC102602350) | mRNA |
| 132 | 102589997 | 3.219 | 0.687 | 30.829 | 6.52E-06 | L-ascorbate_oxidase-like(LOC102589997) | mRNA |
| 133 | 102598951 | 4.507 | 1.858 | 30.401 | 7.25E-06 | probable_xyloglucan_endotransglucosylase/hydrolase_protein_15-like(LOC102598951) | mRNA |
| 134 | 102598758 | 3.922 | 2.044 | 29.766 | 8.48E-06 | probable_xyloglucan_endotransglucosylase/hydrolase_protein_23(LOC102598758) | mRNA |
| 135 | 102582616 | 2.61 | 0.583 | 29.03 | 1.01E-05 | histidine_decarboxylase-like(LOC102582616) | mRNA |
| 136 | 102590956 | 4.692 | 5.723 | 28.914 | 1.05E-05 | uncharacterized(LOC102590956) | mRNA |
| 137 | 102578612 | 2.109 | 2.764 | 28.786 | 1.08E-05 | probably_inactive_leucine-rich_repeat_receptor-like_protein_kinase_At5g48380(LOC102578612) | mRNA |
| 138 | 102580376 | 2.187 | 1.994 | 28.768 | 1.09E-05 | histidine_decarboxylase-like(LOC102580376) | mRNA |
| 139 | 102598017 | 2.065 | 2.18 | 28.351 | 1.21E-05 | cytochrome_P450_78A6-like(LOC102598017) | mRNA |
| 140 | 107062573 | 2.192 | 3.391 | 28.275 | 1.23E-05 | uncharacterized(LOC107062573) | ncRNA |
| 141 | 102599527 | 3.407 | 1.209 | 28.208 | 1.25E-05 | probable_xyloglucan_endotransglucosylase/hydrolase_protein_23(LOC102599527) | mRNA |
| 142 | 102603764 | 2.282 | 3.433 | 27.577 | 1.47E-05 | G-type_lectin_S-receptor-like_serine/threonine-protein_kinase_At4g27290(LOC102603764) | mRNA |
| 143 | 102604704 | 2.633 | 1.116 | 27.478 | 1.51E-05 | protein_ASC1(LOC102604704) | mRNA |
| 144 | 102579389 | 3.612 | 3.85 | 26.918 | 1.75E-05 | protein_EXORDIUM-like(LOC102579389) | mRNA |
| 145 | 102580035 | 3.727 | 2.829 | 26.561 | 1.92E-05 | protein_EXORDIUM-like(LOC102580035) | mRNA |
| 146 | 107059441 | 2.826 | 1.259 | 26.247 | 2.08E-05 | UDP-glycosyltransferase_86A1-like(LOC107059441) | mRNA |
| 147 | 107062289 | 2.014 | 1.256 | 26.183 | 2.11E-05 | mitogen-activated_protein_kinase_kinase_kinase_YODA-like(LOC107062289) | mRNA |
| 148 | 102581197 | 2.103 | 1.241 | 25.825 | 2.32E-05 | wall-associated_receptor_kinase_2-like(LOC102581197) | mRNA |
| 149 | 102579218 | 5.113 | 0.716 | 25.646 | 2.44E-05 | uncharacterized(LOC102579218) | mRNA |
| 150 | 102598411 | 2.898 | 1.551 | 25.251 | 2.72E-05 | uncharacterized(LOC102598411) | ncRNA |
| 151 | 102581954 | 2.193 | 1.578 | 25.107 | 2.83E-05 | histidine_decarboxylase-like(LOC102581954) | mRNA |
| 152 | 102593879 | 2.086 | 0.979 | 24.896 | 2.97E-05 | phosphatidylinositol/phosphatidylcholine_transfer_protein_SFH12-like(LOC102593879) | mRNA |
| 153 | 107062124 | 3.269 | 1.443 | 24.484 | 3.35E-05 | uncharacterized(LOC107062124) | ncRNA |
| 154 | 102580361 | 3.63 | 3.837 | 24.435 | 3.39E-05 | protein_EXORDIUM-like(LOC102580361) | mRNA |
| 155 | 102604187 | 2.341 | 3.861 | 24.074 | 3.75E-05 | ethylene-responsive_transcription_factor_5-like(LOC102604187) | mRNA |
| 156 | 102590669 | 2.203 | 0.831 | 23.883 | 3.92E-05 | (-)-germacrene_D_synthase-like(LOC102590669) | mRNA |
| 157 | 102602533 | 5.327 | 0.792 | 23.636 | 4.23E-05 | uncharacterized(LOC102602533) | mRNA |
| 158 | 102599567 | 2.823 | 2.99 | 23.381 | 4.55E-05 | putative_protein_phosphatase_2C_53(LOC102599567) | mRNA |
| 159 | 102603669 | 4.464 | 1.328 | 23.041 | 5.00E-05 | putative_calcium-binding_protein_CML19(LOC102603669) | mRNA |
| 160 | 102586963 | 2.582 | 3.618 | 22.851 | 5.28E-05 | uncharacterized(LOC102586963) | mRNA |
| 161 | 102582189 | 2.083 | 5.162 | 22.62 | 5.64E-05 | NAD(P)H:quinone_oxidoreductase-like(LOC102582189) | mRNA |
| 162 | 102591555 | 2.584 | 0.895 | 22.575 | 5.71E-05 | uncharacterized(LOC102591555) | ncRNA |
| 163 | 102598046 | 5.47 | 0.163 | 22.309 | 6.16E-05 | ethylene-responsive_transcription_factor_ERF109-like(LOC102598046) | mRNA |
| 164 | 107057930 | 2.035 | 2.106 | 22.226 | 6.31E-05 | probable_leucine-rich_repeat_receptor-like_protein_kinase_At1g35710(LOC107057930) | mRNA |
| 165 | 102583697 | 5.089 | 0.079 | 22.147 | 6.45E-05 | zinc_finger_protein_ZAT11-like(LOC102583697) | mRNA |
| 166 | 102603332 | 3.561 | 1.352 | 22.109 | 6.52E-05 | putative_calcium-binding_protein_CML19(LOC102603332) | mRNA |
| 167 | 107060891 | 2.939 | -0.071 | 22.075 | 6.53E-05 | uncharacterized(LOC107060891) | mRNA |
| 168 | 102577439 | 4.532 | 2.453 | 22.022 | 6.69E-05 | CBF2(LOC102577439) | mRNA |
| 169 | 107058119 | 2.035 | 2.042 | 20.919 | 9.23E-05 | calcium-transporting_ATPase_12_plasma_membrane-type-like(LOC107058119) | mRNA |
| 170 | 102577707 | 5.907 | -0.063 | 20.918 | 9.23E-05 | AP2_domain_CBF_protein(CBF3) | mRNA |
| 171 | 102585371 | 2.673 | 0.945 | 20.893 | 9.30E-05 | probable_glycosyltransferase_At5g03795(LOC102585371) | mRNA |
| 172 | 102599011 | 2.219 | 5.936 | 20.643 | 1.00E-04 | zingipain-2-like(LOC102599011) | mRNA |
| 173 | 102605226 | 3.361 | 1.915 | 20.608 | 1.01E-04 | glucan_endo-13-beta-glucosidase_basic_isoform_1-like(LOC102605226) | misc_RNA |
| 174 | 102578513 | 2.447 | 0.777 | 20.236 | 1.13E-04 | nicotianamine_synthase(LOC102578513) | mRNA |
| 175 | 102579405 | 5.3 | 1.158 | 20.089 | 1.18E-04 | ethylene-responsive_transcription_factor_ERF017(LOC102579405) | mRNA |
| 176 | 102586641 | 3.002 | 0.416 | 20.081 | 1.19E-04 | protein_EARLY_FLOWERING_3-like(LOC102586641) | mRNA |
| 177 | 102604005 | 3.669 | 2.78 | 19.872 | 1.26E-04 | putative_calcium-binding_protein_CML19(LOC102604005) | mRNA |
| 178 | 102591685 | 2.074 | 0.511 | 19.679 | 1.33E-04 | NAC_domain-containing_protein_45-like(LOC102591685) | mRNA |
| 179 | 102597701 | 2.147 | 2.309 | 19.11 | 1.59E-04 | probable_leucine-rich_repeat_receptor-like_protein_kinase_At2g33170(LOC102597701) | mRNA |
| 180 | 102588150 | 3.282 | 5.129 | 19.062 | 1.62E-04 | glycine-rich_RNA-binding_protein-like(LOC102588150) | mRNA |
| 181 | 102589795 | 5.493 | -0.445 | 18.509 | 1.92E-04 | dehydration-responsive_element-binding_protein_1A-like(LOC102589795) | mRNA |
| 182 | 107059978 | 2.357 | 0.753 | 18.369 | 2.01E-04 | uncharacterized(LOC107059978) | ncRNA |
| 183 | 107059435 | 2.556 | 2.264 | 17.856 | 2.36E-04 | U-box_domain-containing_protein_18-like(LOC107059435) | mRNA |
| 184 | 102579714 | 2.605 | 3.079 | 17.827 | 2.38E-04 | protein_EXORDIUM-like(LOC102579714) | mRNA |
| 185 | 102589929 | 2.498 | 1.913 | 17.711 | 2.47E-04 | uncharacterized(LOC102589929) | mRNA |
| 186 | 102580448 | 3.578 | 0.275 | 17.509 | 2.64E-04 | apoptosis-enhancing_nuclease(LOC102580448) | mRNA |
| 187 | 102589387 | 2.016 | 4.133 | 17.034 | 3.07E-04 | uncharacterized(LOC102589387) | mRNA |
| 188 | 102580699 | 3.312 | 3.923 | 16.881 | 3.23E-04 | protein_EXORDIUM-like(LOC102580699) | mRNA |
| 189 | 102578498 | 2.885 | 1.66 | 16.723 | 3.40E-04 | flowering-promoting_factor_1-like_protein_3(LOC102578498) | mRNA |
| 190 | 102579840 | 2.084 | 2.173 | 16.694 | 3.43E-04 | histidine_decarboxylase-like(LOC102579840) | mRNA |
| 191 | 102593849 | 4.988 | 0.32 | 16.134 | 4.13E-04 | putative_calcium-binding_protein_CML19(LOC102593849) | mRNA |
| 192 | 102600960 | 2.353 | 1.533 | 15.647 | 4.85E-04 | myb-related_protein_308-like(LOC102600960) | mRNA |
| 193 | 102597048 | 2.831 | -0.053 | 15.599 | 4.93E-04 | zingipain-2-like(LOC102597048) | mRNA |
| 194 | 102602896 | 12.217 | 4.746 | 17.401 | 4.94E-04 | cytochrome_P450_72A15-like(LOC102602896) | mRNA |
| 195 | 102591626 | 2.066 | 4.403 | 15.274 | 5.51E-04 | uncharacterized(LOC102591626) | mRNA |
| 196 | 102578332 | 2.883 | 0.984 | 15.253 | 5.55E-04 | zinc_finger_protein_ZAT10-like(LOC102578332) | mRNA |
| 197 | 107061036 | 4.713 | -0.138 | 14.914 | 6.23E-04 | uncharacterized(LOC107061036) | mRNA |
| 198 | 102582859 | 2.969 | 0.088 | 14.528 | 7.11E-04 | glucose-6-phosphate/phosphate_translocator_2_chloroplastic-like(LOC102582859) | mRNA |
| 199 | 102604406 | 2.286 | -0.273 | 14.445 | 7.28E-04 | uncharacterized(LOC102604406) | mRNA |
| 200 | 102596073 | 2.204 | 3.301 | 14.274 | 7.77E-04 | protein_HYPER-SENSITIVITY-RELATED_4-like(LOC102596073) | mRNA |
| 201 | 102590997 | 2.529 | 0.316 | 14.188 | 8.00E-04 | basic_form_of_pathogenesis-related_protein_1-like(LOC102590997) | mRNA |
| 202 | 102605139 | 2.006 | 1.798 | 14.172 | 8.05E-04 | uncharacterized(LOC102605139) | mRNA |
| 203 | 102605651 | 2.313 | -0.085 | 14.005 | 8.50E-04 | uncharacterized(LOC102605651) | mRNA |
| 204 | 102589441 | 2.582 | 0.026 | 13.906 | 8.83E-04 | basic_form_of_pathogenesis-related_protein_1-like(LOC102589441) | mRNA |
| 205 | 102602250 | 2.023 | 0.646 | 13.841 | 9.04E-04 | protein_NDR1-like(LOC102602250) | mRNA |
| 206 | 102583793 | 2.055 | 0.836 | 13.834 | 9.06E-04 | copal-8-ol_diphosphate_hydratase_chloroplastic-like(LOC102583793) | mRNA |

**D. Downregulated genes between ATLWT (Mock) and ATLWT(PVY-inoculated)**

| Rank | GeneID | LogFC | LogCPM | F | P_value | Annotation | RNAtype |
| --- | --- | --- | --- | --- | --- | --- | --- |
|  |  |  |  |  |  |  |  |
| 1 | 102600070 | -3.124 | 1.523 | 86.202 | 5.73E-10 | uncharacterized(LOC102600070) | mRNA |
| 2 | 102606049 | -6.483 | 4.494 | 85.253 | 6.47E-10 | fidgetin-like_protein_1(LOC102606049) | mRNA |
| 3 | 102587091 | -5.147 | 3.288 | 79.813 | 1.29E-09 | uncharacterized(LOC102587091) | ncRNA |
| 4 | 102595671 | -2.095 | 4.769 | 76.382 | 2.02E-09 | early_endosome_antigen_1(LOC102595671) | mRNA |
| 5 | 102592828 | -2.723 | 2.206 | 75.695 | 2.22E-09 | stearoyl-[acyl-carrier-protein]_9-desaturase_1_chloroplastic-like(LOC102592828) | mRNA |
| 6 | 102589300 | -3.478 | 3.417 | 74.348 | 2.66E-09 | alanine--glyoxylate_aminotransferase_2_homolog_2_mitochondrial(LOC102589300) | mRNA |
| 7 | 102591434 | -6.667 | 3.771 | 74.14 | 2.74E-09 | uncharacterized(LOC102591434) | mRNA |
| 8 | 102593309 | -6.96 | 1.799 | 73.488 | 2.99E-09 | protein_BIG_GRAIN_1-like_B(LOC102593309) | mRNA |
| 9 | 102599238 | -4.591 | 0.088 | 71.536 | 3.81E-09 | (EE)-geranyllinalool_synthase(LOC102599238) | mRNA |
| 10 | 102600114 | -5.016 | 4.456 | 70.852 | 4.33E-09 | cell_division_cycle_protein_48_homolog(LOC102600114) | mRNA |
| 11 | 102598584 | -2.61 | 0.365 | 70.475 | 4.40E-09 | ABC_transporter_G_family_member_4-like(LOC102598584) | mRNA |
| 12 | 102592897 | -5.49 | 0.404 | 69.143 | 5.52E-09 | galactinol_synthase_2-like(LOC102592897) | mRNA |
| 13 | 102603811 | -5.096 | 5.853 | 68.959 | 5.67E-09 | ATP-dependent_zinc_metalloprotease_FTSH_6_chloroplastic(LOC102603811) | mRNA |
| 14 | 102590098 | -2.755 | 4.194 | 67.177 | 7.35E-09 | tetraketide_alpha-pyrone_reductase_1-like(LOC102590098) | mRNA |
| 15 | 102589092 | -4.688 | 4.259 | 65.617 | 9.27E-09 | homeobox-leucine_zipper_protein_ATHB-7-like(LOC102589092) | mRNA |
| 16 | 102599550 | -6.698 | 2.977 | 64.783 | 1.05E-08 | expansin-like_B1(LOC102599550) | mRNA |
| 17 | 107057685 | -7.476 | 10.205 | 62.97 | 1.39E-08 | abscisic_acid_and_environmental_stress-inducible_protein_TAS14-like(LOC107057685) | mRNA |
| 18 | 102596667 | -5.228 | 5.033 | 61.466 | 1.75E-08 | low-temperature-induced_65_kDa_protein-like(LOC102596667) | mRNA |
| 19 | 102591072 | -2.229 | 3.717 | 59.536 | 2.38E-08 | phosphoenolpyruvate_carboxylase_kinase_2-like(LOC102591072) | mRNA |
| 20 | 102579007 | -3.026 | 5.64 | 58.616 | 2.76E-08 | protein_ECERIFERUM_1-like(LOC102579007) | mRNA |
| 21 | 102591763 | -3.766 | 2.011 | 58.359 | 2.87E-08 | branched-chain-amino-acid_aminotransferase_2_chloroplastic-like(LOC102591763) | mRNA |
| 22 | 102577575 | -3.363 | 5.505 | 57.366 | 3.38E-08 | sucrose_synthase(LOC102577575) | mRNA |
| 23 | 102584503 | -7.636 | 5.852 | 57.26 | 3.44E-08 | small_heat_shock_protein_chloroplastic(LOC102584503) | mRNA |
| 24 | 102591696 | -2.185 | 4.261 | 56.809 | 3.71E-08 | uncharacterized(LOC102591696) | mRNA |
| 25 | 102603300 | -5.397 | 2.025 | 56.537 | 3.88E-08 | NADP-dependent_malic_enzyme(LOC102603300) | mRNA |
| 26 | 102600343 | -3.837 | -0.73 | 56.112 | 4.04E-08 | alpha-farnesene_synthase-like(LOC102600343) | misc_RNA |
| 27 | 102581644 | -3.114 | 1.045 | 55.356 | 4.72E-08 | poly_[ADP-ribose]_polymerase_3(LOC102581644) | mRNA |
| 28 | 102591596 | -5.724 | 0.167 | 54.912 | 5.09E-08 | uncharacterized(LOC102591596) | mRNA |
| 29 | 102585726 | -2.952 | 2.842 | 52.232 | 8.08E-08 | homeobox-leucine_zipper_protein_ATHB-7-like(LOC102585726) | mRNA |
| 30 | 102595227 | -5.295 | 0.663 | 51.69 | 8.89E-08 | late_embryogenesis_abundant_protein_D-29(LOC102595227) | mRNA |
| 31 | 102598306 | -4.696 | 3.209 | 51.688 | 8.90E-08 | SNF1-related_protein_kinase_regulatory_subunit_gamma-like_PV42a(LOC102598306) | mRNA |
| 32 | 102601737 | -5.387 | 3.117 | 51.413 | 9.34E-08 | probable_sodium-coupled_neutral_amino_acid_transporter_6(LOC102601737) | mRNA |
| 33 | 102588159 | -2.381 | 5.092 | 50.718 | 1.06E-07 | beta-galactosidase-like(LOC102588159) | mRNA |
| 34 | 102594742 | -2.488 | 3.768 | 48.869 | 1.48E-07 | uncharacterized(LOC102594742) | mRNA |
| 35 | 102589744 | -2.96 | 6.158 | 48.753 | 1.51E-07 | methionine_gamma-lyase-like(LOC102589744) | mRNA |
| 36 | 102585136 | -4.952 | 2.61 | 48.515 | 1.58E-07 | non-specific_lipid-transfer_protein_2-like(LOC102585136) | mRNA |
| 37 | 102602565 | -5.309 | 1.65 | 47.327 | 1.97E-07 | low-temperature-induced_78_kDa_protein-like(LOC102602565) | mRNA |
| 38 | 102593355 | -5.109 | -0.256 | 47.261 | 1.98E-07 | seed_biotin-containing_protein_SBP65-like(LOC102593355) | mRNA |
| 39 | 102605148 | -4.74 | 2.084 | 47.186 | 2.02E-07 | protein_LE25-like(LOC102605148) | mRNA |
| 40 | 107061200 | -2.446 | 2.467 | 47.04 | 2.07E-07 | uncharacterized(LOC107061200) | ncRNA |
| 41 | 107059770 | -2.626 | 2.062 | 46.894 | 2.13E-07 | uncharacterized(LOC107059770) | ncRNA |
| 42 | 102584798 | -3.023 | -0.355 | 46.488 | 2.25E-07 | protein_TIFY_10A-like(LOC102584798) | mRNA |
| 43 | 102588806 | -2.17 | 2.72 | 46.188 | 2.44E-07 | phosphoglucan_phosphatase_DSP4_amyloplastic-like(LOC102588806) | mRNA |
| 44 | 102592278 | -4.95 | 2.001 | 46.072 | 2.49E-07 | expansin-like_B1(LOC102592278) | mRNA |
| 45 | 102595755 | -4.185 | 0.115 | 46.034 | 2.50E-07 | agmatine_coumaroyltransferase-2-like(LOC102595755) | mRNA |
| 46 | 102598452 | -4.289 | 4.838 | 45.977 | 2.54E-07 | auxin-repressed_12.5_kDa_protein-like(LOC102598452) | mRNA |
| 47 | 102582408 | -3.643 | 5.408 | 45.677 | 2.69E-07 | probable_protein_phosphatase_2C_51(LOC102582408) | mRNA |
| 48 | 102598218 | -4.047 | 4.497 | 45.252 | 2.92E-07 | translocator_protein_homolog(LOC102598218) | mRNA |
| 49 | 102581791 | -2.197 | 0.957 | 44.808 | 3.10E-07 | EPIDERMAL_PATTERNING_FACTOR-like_protein_2(LOC102581791) | mRNA |
| 50 | 102603718 | -2.14 | 0.875 | 44.778 | 3.12E-07 | uclacyanin-3-like(LOC102603718) | mRNA |
| 51 | 102581483 | -3.49 | 1.552 | 44.706 | 3.24E-07 | heavy_metal-associated_isoprenylated_plant_protein_26-like(LOC102581483) | mRNA |
| 52 | 102591579 | -3.128 | 2.225 | 44.507 | 3.37E-07 | uncharacterized(LOC102591579) | mRNA |
| 53 | 102600309 | -4.678 | 6.86 | 44.169 | 3.60E-07 | stromal_70_kDa_heat_shock-related_protein_chloroplastic-like(LOC102600309) | mRNA |
| 54 | 102591828 | -3.77 | 3.38 | 44.111 | 3.64E-07 | heat_shock_factor_protein_HSF30-like(LOC102591828) | mRNA |
| 55 | 102586060 | -4.292 | 4.142 | 43.7 | 3.94E-07 | nuclear_transcription_factor_Y_subunit_A-7-like(LOC102586060) | mRNA |
| 56 | 102584616 | -4.627 | 2.118 | 43.409 | 4.18E-07 | uncharacterized(LOC102584616) | mRNA |
| 57 | 102591902 | -3.857 | 4.741 | 43.1 | 4.44E-07 | bidirectional_sugar_transporter_SWEET12-like(LOC102591902) | mRNA |
| 58 | 102580981 | -4.869 | 3.538 | 42.251 | 5.26E-07 | heavy_metal-associated_isoprenylated_plant_protein_26-like(LOC102580981) | mRNA |
| 59 | 102585181 | -2.956 | 1.012 | 42.211 | 5.31E-07 | inorganic_pyrophosphatase_1-like(LOC102585181) | mRNA |
| 60 | 102584601 | -2.419 | 3.045 | 42.089 | 5.44E-07 | heat_shock_70_kDa_protein-like(LOC102584601) | mRNA |
| 61 | 102577501 | -2.999 | 7.911 | 41.49 | 6.14E-07 | non-specific_lipid_transfer_protein_a7(LOC102577501) | mRNA |
| 62 | 102587642 | -2.577 | 2.652 | 41.456 | 6.18E-07 | nuclear_transcription_factor_Y_subunit_A-10-like(LOC102587642) | mRNA |
| 63 | 102594072 | -5.848 | -0.788 | 40.931 | 6.78E-07 | butyrate--CoA_ligase_AAE11_peroxisomal-like(LOC102594072) | mRNA |
| 64 | 102589273 | -2.754 | 3.405 | 40.898 | 6.93E-07 | PI-PLC_X-box_domain-containing_protein_DDB_G0293730-like(LOC102589273) | mRNA |
| 65 | 102589363 | -4.736 | 3.91 | 40.599 | 7.37E-07 | non-specific_lipid-transfer_protein_2-like(LOC102589363) | mRNA |
| 66 | 102599348 | -3.933 | 4.925 | 40.381 | 7.71E-07 | protein_phosphatase_2C_37-like(LOC102599348) | mRNA |
| 67 | 102601468 | -2.446 | 3.183 | 40.273 | 7.88E-07 | myb-related_protein_306(LOC102601468) | mRNA |
| 68 | 102601897 | -3.562 | -0.609 | 39.433 | 9.20E-07 | growth-regulating_factor_1(LOC102601897) | mRNA |
| 69 | 102594043 | -4.759 | 3.717 | 39.509 | 9.25E-07 | peptidyl-prolyl_cis-trans_isomerase_FKBP62-like(LOC102594043) | mRNA |
| 70 | 102587639 | -4.194 | -0.405 | 39.075 | 9.98E-07 | 18.1_kDa_class_I_heat_shock_protein-like(LOC102587639) | mRNA |
| 71 | 102591853 | -5.537 | 6.583 | 38.847 | 1.06E-06 | small_heat_shock_protein_chloroplastic-like(LOC102591853) | mRNA |
| 72 | 102585909 | -2.253 | 1.051 | 38.7 | 1.09E-06 | basic_leucine_zipper_63(LOC102585909) | mRNA |
| 73 | 102581388 | -3.232 | 3.609 | 38.742 | 1.09E-06 | glutamate_decarboxylase(LOC102581388) | mRNA |
| 74 | 102595164 | -2.03 | 4.902 | 37.924 | 1.30E-06 | uncharacterized(LOC102595164) | mRNA |
| 75 | 102603215 | -3.231 | 3.584 | 37.681 | 1.36E-06 | protein_NRT1/_PTR_FAMILY_6.3-like(LOC102603215) | mRNA |
| 76 | 102603803 | -2.82 | 3.974 | 37.102 | 1.55E-06 | GDSL_esterase/lipase_EXL3-like(LOC102603803) | mRNA |
| 77 | 102579555 | -2.213 | 1.941 | 37.057 | 1.56E-06 | oxygen-evolving_enhancer_protein_2_chloroplastic-like(LOC102579555) | mRNA |
| 78 | 102589326 | -4.266 | 0.866 | 36.857 | 1.63E-06 | uncharacterized(LOC102589326) | mRNA |
| 79 | 102606179 | -4.388 | 5.988 | 36.326 | 1.83E-06 | 18.2_kDa_class_I_heat_shock_protein-like(LOC102606179) | mRNA |
| 80 | 102603559 | -3.029 | 3.235 | 36.142 | 1.91E-06 | uncharacterized_protein_OsI_027940-like(LOC102603559) | mRNA |
| 81 | 102591190 | -3.493 | 3.32 | 35.829 | 2.05E-06 | 17.4_kDa_class_III_heat_shock_protein(LOC102591190) | mRNA |
| 82 | 102579854 | -2.488 | 1.254 | 35.797 | 2.06E-06 | protein_NRT1/_PTR_FAMILY_2.8(LOC102579854) | mRNA |
| 83 | 102598583 | -3.126 | 4.379 | 35.202 | 2.36E-06 | non-specific_lipid-transfer_protein_2-like(LOC102598583) | mRNA |
| 84 | 102589775 | -2.602 | 0.085 | 34.974 | 2.43E-06 | extensin-3-like(LOC102589775) | mRNA |
| 85 | 102596945 | -2.928 | 4.987 | 35.023 | 2.45E-06 | uncharacterized(LOC102596945) | mRNA |
| 86 | 102597495 | -2.425 | 2.136 | 34.771 | 2.60E-06 | peroxygenase-like(LOC102597495) | mRNA |
| 87 | 102588710 | -4.689 | 4.206 | 34.624 | 2.68E-06 | non-specific_lipid-transfer_protein_2-like(LOC102588710) | mRNA |
| 88 | 102579662 | -2.429 | 2.714 | 34.399 | 2.82E-06 | mitochondrial_arginine_transporter_BAC2(LOC102579662) | mRNA |
| 89 | 102580603 | -2.571 | 0.382 | 34.16 | 2.94E-06 | uncharacterized(LOC102580603) | mRNA |
| 90 | 102606436 | -3.854 | 6.037 | 34.157 | 2.99E-06 | 17.6_kDa_class_I_heat_shock_protein(LOC102606436) | mRNA |
| 91 | 107058400 | -3.18 | 0.342 | 34.136 | 3.00E-06 | calsequestrin-1-like(LOC107058400) | mRNA |
| 92 | 102580977 | -4.034 | 0.137 | 34.001 | 3.09E-06 | beta-amyrin_28-oxidase-like(LOC102580977) | mRNA |
| 93 | 102587898 | -3.031 | 0.007 | 33.923 | 3.11E-06 | type_I_inositol_polyphosphate_5-phosphatase_8-like(LOC102587898) | mRNA |
| 94 | 102577576 | -3.807 | 6.684 | 33.848 | 3.20E-06 | asparagine_synthetase_[glutamine-hydrolyzing](LOC102577576) | mRNA |
| 95 | 102584371 | -5.537 | 3.831 | 33.844 | 3.21E-06 | 22.7_kDa_class_IV_heat_shock_protein-like(LOC102584371) | mRNA |
| 96 | 102597309 | -2.885 | 6.296 | 33.729 | 3.29E-06 | non-specific_lipid-transfer_protein_2-like(LOC102597309) | mRNA |
| 97 | 102583716 | -3.774 | 0.067 | 33.5 | 3.47E-06 | polyneuridine-aldehyde_esterase-like(LOC102583716) | mRNA |
| 98 | 107059349 | -2.819 | 1.358 | 33.454 | 3.51E-06 | uncharacterized(LOC107059349) | ncRNA |
| 99 | 102594103 | -3.204 | 4.606 | 32.82 | 4.07E-06 | 15.7_kDa_heat_shock_protein_peroxisomal(LOC102594103) | mRNA |
| 100 | 102593790 | -2.818 | 0.122 | 32.637 | 4.19E-06 | histone_H2B_7-like(LOC102593790) | mRNA |
| 101 | 102597686 | -2.05 | 3.594 | 32.683 | 4.20E-06 | late_embryogenesis_abundant_protein-like(LOC102597686) | mRNA |
| 102 | 102600701 | -2.06 | 6.067 | 32.655 | 4.23E-06 | basic_blue_protein-like(LOC102600701) | mRNA |
| 103 | 102601122 | -2.853 | 3.159 | 32.525 | 4.36E-06 | calcium_uniporter_protein_2_mitochondrial(LOC102601122) | mRNA |
| 104 | 102601369 | -4.051 | 2.032 | 32.313 | 4.58E-06 | uncharacterized(LOC102601369) | mRNA |
| 105 | 102594426 | -2.355 | 2.511 | 32.254 | 4.65E-06 | ankyrin_repeat-containing_protein_At3g12360-like(LOC102594426) | mRNA |
| 106 | 102597083 | -2.34 | 4.462 | 32.25 | 4.65E-06 | ethylene-responsive_transcription_factor_1-like(LOC102597083) | mRNA |
| 107 | 102583805 | -2.341 | 3.034 | 32.241 | 4.66E-06 | putative_uncharacterized_transmembrane_protein_DDB_G0293028(LOC102583805) | mRNA |
| 108 | 102604599 | -3.995 | 2.159 | 32.05 | 4.88E-06 | luminal-binding_protein_5(LOC102604599) | mRNA |
| 109 | 102592090 | -4.736 | 0.425 | 31.674 | 5.33E-06 | late_embryogenesis_abundant_protein_2-like(LOC102592090) | mRNA |
| 110 | 107057990 | -2.459 | 0.493 | 31.585 | 5.40E-06 | uncharacterized(LOC107057990) | ncRNA |
| 111 | 102601889 | -5.045 | 2.473 | 31.472 | 5.60E-06 | uncharacterized(LOC102601889) | mRNA |
| 112 | 107059500 | -2.37 | 0.763 | 31.447 | 5.60E-06 | uncharacterized(LOC107059500) | ncRNA |
| 113 | 107059214 | -2.558 | 0.155 | 31.406 | 5.61E-06 | uncharacterized(LOC107059214) | mRNA |
| 114 | 102584858 | -2.195 | 4.287 | 31.343 | 5.77E-06 | uncharacterized(LOC102584858) | ncRNA |
| 115 | 102591877 | -3.203 | 5.301 | 31.25 | 5.90E-06 | delta-amyrin_synthase(LOC102591877) | mRNA |
| 116 | 102593334 | -2.559 | 3.361 | 31.227 | 5.94E-06 | telomerase_reverse_transcriptase(LOC102593334) | mRNA |
| 117 | 102605643 | -2.363 | 3.392 | 30.897 | 6.43E-06 | uncharacterized(LOC102605643) | ncRNA |
| 118 | 102606295 | -3.493 | 8.12 | 30.878 | 6.46E-06 | probable_zinc_metallopeptidase_EGY3_chloroplastic(LOC102606295) | mRNA |
| 119 | 102580640 | -4.117 | 0.257 | 30.786 | 6.60E-06 | heavy_metal-associated_isoprenylated_plant_protein_26-like(LOC102580640) | mRNA |
| 120 | 102597343 | -2.336 | 0.272 | 30.668 | 6.69E-06 | uncharacterized(LOC102597343) | mRNA |
| 121 | 102599419 | -3.384 | 5.383 | 30.673 | 6.79E-06 | bidirectional_sugar_transporter_SWEET15-like(LOC102599419) | mRNA |
| 122 | 102586370 | -3.987 | 1.3 | 30.562 | 6.97E-06 | BURP_domain-containing_protein_17-like(LOC102586370) | mRNA |
| 123 | 102583475 | -2.588 | 4.393 | 30.261 | 7.51E-06 | heat_shock_70_kDa_protein_8(LOC102583475) | mRNA |
| 124 | 102606159 | -2.688 | 0.901 | 30.244 | 7.54E-06 | peroxidase_46-like(LOC102606159) | mRNA |
| 125 | 102580120 | -5.207 | 7.07 | 30.224 | 7.57E-06 | expansin-like_B1(LOC102580120) | mRNA |
| 126 | 102605260 | -3.611 | 1.347 | 29.936 | 8.13E-06 | patatin-05-like(LOC102605260) | mRNA |
| 127 | 102606273 | -2.689 | 5.614 | 29.645 | 8.74E-06 | extensin-1-like(LOC102606273) | mRNA |
| 128 | 102590242 | -3.886 | 8.147 | 29.643 | 8.74E-06 | ribulose_bisphosphate_carboxylase/oxygenase_activase_1_chloroplastic-like(LOC102590242) | mRNA |
| 129 | 102591975 | -2.075 | 0.658 | 29.334 | 9.32E-06 | protein_YLS9-like(LOC102591975) | mRNA |
| 130 | 102577712 | -2.581 | 6.193 | 29.107 | 9.99E-06 | sucrose_synthase_2(LOC102577712) | mRNA |
| 131 | 102602655 | -2.894 | 2.006 | 29.107 | 9.99E-06 | uncharacterized(LOC102602655) | mRNA |
| 132 | 102578442 | -4.84 | 5.268 | 28.827 | 1.07E-05 | 17.7_kDa_class_I_heat_shock_protein-like(LOC102578442) | mRNA |
| 133 | 102578969 | -4.754 | 3.329 | 28.828 | 1.07E-05 | 26.5_kDa_heat_shock_protein_mitochondrial(LOC102578969) | mRNA |
| 134 | 102577794 | -4.57 | 4.313 | 28.406 | 1.19E-05 | uncharacterized(LOC102577794) | mRNA |
| 135 | 102580548 | -2.926 | -0.419 | 28.307 | 1.21E-05 | putative_cytochrome_c_oxidase_subunit_6b-like(LOC102580548) | mRNA |
| 136 | 102584557 | -4.114 | 1.966 | 28.196 | 1.26E-05 | uncharacterized(LOC102584557) | mRNA |
| 137 | 102600881 | -2.544 | 4.43 | 28.184 | 1.26E-05 | chaperone_protein_dnaJ_11_chloroplastic-like(LOC102600881) | mRNA |
| 138 | 102598254 | -2.431 | 5.884 | 28.046 | 1.31E-05 | DNA(cytosine-5)-methyltransferase_DRM2-like(LOC102598254) | mRNA |
| 139 | 107059789 | -2.933 | 0.831 | 28.034 | 1.31E-05 | uncharacterized(LOC107059789) | ncRNA |
| 140 | 102592302 | -3.671 | 3.048 | 27.998 | 1.32E-05 | tyrosine_decarboxylase_1-like(LOC102592302) | mRNA |
| 141 | 102599466 | -2.388 | 2.237 | 27.953 | 1.34E-05 | serine/threonine-protein_kinase_SAPK7-like(LOC102599466) | mRNA |
| 142 | 102584607 | -4.276 | -0.865 | 27.818 | 1.37E-05 | ABA-inducible_protein_PHV_A1-like(LOC102584607) | mRNA |
| 143 | 102601494 | -3.713 | 5.042 | 27.59 | 1.47E-05 | 22.7_kDa_class_IV_heat_shock_protein-like(LOC102601494) | mRNA |
| 144 | 102598275 | -2.118 | 4.636 | 27.373 | 1.55E-05 | uncharacterized(LOC102598275) | mRNA |
| 145 | 102587505 | -3.019 | 1.538 | 27.275 | 1.59E-05 | zeatin_O-glucosyltransferase-like(LOC102587505) | mRNA |
| 146 | 102584365 | -2.325 | 3.77 | 26.876 | 1.77E-05 | uncharacterized(LOC102584365) | mRNA |
| 147 | 102593672 | -2.154 | 4.245 | 26.795 | 1.80E-05 | tetraketide_alpha-pyrone_reductase_1-like(LOC102593672) | mRNA |
| 148 | 102597777 | -3.088 | 3.364 | 26.632 | 1.88E-05 | uncharacterized(LOC102597777) | mRNA |
| 149 | 102604849 | -4.198 | 0.031 | 26.35 | 2.03E-05 | axial_regulator_YABBY_1(LOC102604849) | mRNA |
| 150 | 102590407 | -2.271 | 0.208 | 26.258 | 2.05E-05 | zinc-finger_homeodomain_protein_2-like(LOC102590407) | mRNA |
| 151 | 102598463 | -2.767 | -0.23 | 26.062 | 2.17E-05 | probable_ascorbate-specific_transmembrane_electron_transporter_1(LOC102598463) | mRNA |
| 152 | 102599311 | -2.095 | 1.238 | 25.996 | 2.23E-05 | cytochrome_c_oxidase_subunit_5C-like(LOC102599311) | mRNA |
| 153 | 102601370 | -2.546 | 4.724 | 25.87 | 2.30E-05 | uncharacterized(LOC102601370) | mRNA |
| 154 | 102599001 | -2.433 | 0.152 | 25.682 | 2.40E-05 | uncharacterized(LOC102599001) | ncRNA |
| 155 | 102582967 | -3.764 | 1.88 | 25.58 | 2.49E-05 | class_I_heat_shock_protein-like(LOC102582967) | mRNA |
| 156 | 102605801 | -4.145 | -1.16 | 25.489 | 2.52E-05 | defensin-like_protein(LOC102605801) | mRNA |
| 157 | 102584868 | -2.694 | 1.73 | 25.312 | 2.67E-05 | inorganic_pyrophosphatase_1-like(LOC102584868) | mRNA |
| 158 | 102598628 | -4.098 | -0.377 | 24.939 | 2.96E-05 | oleosin_5-like(LOC102598628) | mRNA |
| 159 | 102578188 | -3.483 | 6.553 | 24.444 | 3.39E-05 | 18.2_kDa_class_I_heat_shock_protein-like(LOC102578188) | mRNA |
| 160 | 102577597 | -2.102 | 8.05 | 24.254 | 3.57E-05 | chaperone_protein_ClpB1(LOC102577597) | mRNA |
| 161 | 102589195 | -2.448 | 0.582 | 24.224 | 3.60E-05 | 1-aminocyclopropane-1-carboxylate_oxidase_5-like(LOC102589195) | mRNA |
| 162 | 102588418 | -4.219 | 6.29 | 24.223 | 3.60E-05 | heat_shock_70_kDa_protein_5(LOC102588418) | mRNA |
| 163 | 102597956 | -4.1 | 2.741 | 24.201 | 3.62E-05 | protodermal_factor_1(LOC102597956) | mRNA |
| 164 | 102599929 | -2.262 | 0.498 | 24.161 | 3.64E-05 | RING-H2_finger_protein_ATL78-like(LOC102599929) | mRNA |
| 165 | 102592057 | -2.355 | 11.661 | 23.851 | 3.99E-05 | BAG_family_molecular_chaperone_regulator_6(LOC102592057) | mRNA |
| 166 | 107058610 | -2.676 | 1.492 | 23.84 | 4.00E-05 | photosystem_II_5_kDa_protein_chloroplastic(LOC107058610) | mRNA |
| 167 | 102583036 | -2.15 | 1.786 | 23.837 | 4.00E-05 | extensin-3-like(LOC102583036) | mRNA |
| 168 | 102586866 | -3.546 | 0.245 | 23.684 | 4.18E-05 | uncharacterized(LOC102586866) | mRNA |
| 169 | 102586553 | -2.477 | 0.957 | 23.395 | 4.53E-05 | uncharacterized_protein_DDB_G0271670-like(LOC102586553) | mRNA |
| 170 | 102588485 | -2.375 | 2.113 | 23.364 | 4.57E-05 | CASP-like_protein_2B1(LOC102588485) | mRNA |
| 171 | 102594541 | -2.002 | 7.88 | 23.186 | 4.80E-05 | delta-1-pyrroline-5-carboxylate_synthase-like(LOC102594541) | mRNA |
| 172 | 102589043 | -3.837 | 2.48 | 23.034 | 5.01E-05 | non-specific_lipid-transfer_protein_2-like(LOC102589043) | mRNA |
| 173 | 102597952 | -2.729 | 2.835 | 22.813 | 5.33E-05 | non-specific_lipid-transfer_protein_1(LOC102597952) | mRNA |
| 174 | 102598867 | -2.397 | 6.406 | 22.636 | 5.61E-05 | small_heat_shock_protein_chloroplastic-like(LOC102598867) | mRNA |
| 175 | 102598817 | -3.197 | 9.737 | 22.529 | 5.78E-05 | inositol-3-phosphate_synthase(LOC102598817) | mRNA |
| 176 | 102602048 | -3.409 | 1.943 | 22.424 | 5.96E-05 | patatin-T5-like(LOC102602048) | mRNA |
| 177 | 107062126 | -4.05 | -0.143 | 22.381 | 6.03E-05 | 14_kDa_proline-rich_protein_DC2.15-like(LOC107062126) | mRNA |
| 178 | 102581810 | -2.587 | 6.176 | 22.242 | 6.28E-05 | heat_shock_protein_83-like(LOC102581810) | misc_RNA |
| 179 | 102587597 | -2.872 | 0.826 | 22.227 | 6.31E-05 | uncharacterized(LOC102587597) | mRNA |
| 180 | 107057688 | -2.038 | 0.309 | 22.196 | 6.31E-05 | probable_long-chain-alcohol_O-fatty-acyltransferase_5(LOC107057688) | mRNA |
| 181 | 102598162 | -2.705 | -0.486 | 22.007 | 6.65E-05 | oleosin_1-like(LOC102598162) | mRNA |
| 182 | 102579619 | -2.242 | 3.881 | 21.851 | 7.03E-05 | transcriptional_activator_TAF-1-like(LOC102579619) | mRNA |
| 183 | 102583506 | -3.013 | 2.941 | 21.794 | 7.15E-05 | gibberellin-regulated_protein_10-like(LOC102583506) | mRNA |
| 184 | 102580487 | -2.326 | 5.975 | 21.623 | 7.51E-05 | pleiotropic_drug_resistance_protein_2-like(LOC102580487) | mRNA |
| 185 | 107060836 | -3.607 | -0.1 | 21.501 | 7.78E-05 | heavy_metal-associated_isoprenylated_plant_protein_26-like(LOC107060836) | mRNA |
| 186 | 107063241 | -2.132 | 1.631 | 21.396 | 8.02E-05 | uncharacterized(LOC107063241) | ncRNA |
| 187 | 102579945 | -3.548 | -1.214 | 21.346 | 8.06E-05 | GDSL_esterase/lipase_At4g01130-like(LOC102579945) | mRNA |
| 188 | 102581790 | -2.579 | 0.878 | 20.957 | 9.13E-05 | uncharacterized_membrane_protein_At3g27390(LOC102581790) | mRNA |
| 189 | 102591283 | -2.325 | 2.535 | 20.924 | 9.22E-05 | thaumatin-like_protein_1(LOC102591283) | mRNA |
| 190 | 102584159 | -2.887 | 1.523 | 20.863 | 9.39E-05 | uncharacterized(LOC102584159) | ncRNA |
| 191 | 102605425 | -3.485 | 8.736 | 20.847 | 9.43E-05 | 17.8_kDa_class_I_heat_shock_protein(LOC102605425) | mRNA |
| 192 | 102594516 | -3.773 | 0.178 | 20.568 | 1.02E-04 | branched-chain-amino-acid_aminotransferase_2_chloroplastic-like(LOC102594516) | mRNA |
| 193 | 102601036 | -2.995 | 0.488 | 20.436 | 1.07E-04 | patatin-02-like(LOC102601036) | mRNA |
| 194 | 102590866 | -2.748 | -0.87 | 20.131 | 1.16E-04 | homeobox_protein_knotted-1-like_1-like(LOC102590866) | mRNA |
| 195 | 102580235 | -2.27 | 0.623 | 20.093 | 1.18E-04 | probable_LRR_receptor-like_serine/threonine-protein_kinase_At4g36180(LOC102580235) | mRNA |
| 196 | 102596331 | -2.857 | -0.097 | 19.836 | 1.28E-04 | uncharacterized(LOC102596331) | ncRNA |
| 197 | 102606174 | -2.097 | 0.139 | 19.801 | 1.28E-04 | bZIP_transcription_factor_53-like(LOC102606174) | mRNA |
| 198 | 102589078 | -3.165 | 4.937 | 19.513 | 1.41E-04 | 17.4_kDa_class_I_heat_shock_protein-like(LOC102589078) | mRNA |
| 199 | 102585180 | -2.343 | 9.241 | 19.242 | 1.53E-04 | E3_ubiquitin-protein_ligase_MIEL1(LOC102585180) | mRNA |
| 200 | 102588746 | -2.868 | 5.44 | 19.108 | 1.60E-04 | 18.2_kDa_class_I_heat_shock_protein-like(LOC102588746) | mRNA |
| 201 | 102603518 | -3.474 | 2.976 | 18.908 | 1.70E-04 | 22.7_kDa_class_IV_heat_shock_protein(LOC102603518) | mRNA |
| 202 | 102578720 | -3.202 | 6.288 | 18.907 | 1.70E-04 | L-ascorbate_peroxidase_cytosolic(LOC102578720) | mRNA |
| 203 | 102589396 | -2.265 | 4.952 | 18.908 | 1.70E-04 | 17.4_kDa_class_I_heat_shock_protein-like(LOC102589396) | mRNA |
| 204 | 102594883 | -2.265 | 6.93 | 18.833 | 1.74E-04 | heat_shock_cognate_70_kDa_protein(LOC102594883) | mRNA |
| 205 | 102595526 | -3.078 | -0.546 | 18.786 | 1.76E-04 | abscisic_acid_and_environmental_stress-inducible_protein_TAS14(LOC102595526) | mRNA |
| 206 | 102599819 | -3.943 | 1.836 | 18.794 | 1.76E-04 | ferredoxin-like(LOC102599819) | mRNA |
| 207 | 102601415 | -2.436 | 1.889 | 18.732 | 1.79E-04 | GDSL_esterase/lipase_At1g71691-like(LOC102601415) | mRNA |
| 208 | 102593393 | -3.522 | -0.763 | 18.559 | 1.89E-04 | nectarin-1-like(LOC102593393) | mRNA |
| 209 | 102585024 | -2.243 | 0.87 | 18.538 | 1.90E-04 | AP2-like_ethylene-responsive_transcription_factor_ANT(LOC102585024) | mRNA |
| 210 | 102584003 | -2.089 | 1.293 | 18.383 | 2.00E-04 | transcription_factor_MYB82-like(TSF) | mRNA |
| 211 | 102597267 | -2.223 | 1.637 | 18.277 | 2.07E-04 | gibberellin_20_oxidase_1(LOC102597267) | mRNA |
| 212 | 102588952 | -2.61 | -0.146 | 17.825 | 2.38E-04 | PI-PLC_X-box_domain-containing_protein_DDB_G0293730-like(LOC102588952) | mRNA |
| 213 | 102586690 | -2.959 | -0.174 | 17.705 | 2.48E-04 | seed_biotin-containing_protein_SBP65-like(LOC102586690) | mRNA |
| 214 | 102578786 | -4.06 | 6.642 | 17.586 | 2.57E-04 | 17.6_kDa_class_I_heat_shock_protein-like(LOC102578786) | mRNA |
| 215 | 102582525 | -3.463 | 5.371 | 17.494 | 2.65E-04 | putative_glycine-rich_cell_wall_structural_protein_1(LOC102582525) | mRNA |
| 216 | 107059991 | -3.442 | 0.623 | 17.482 | 2.66E-04 | uncharacterized(LOC107059991) | ncRNA |
| 217 | 102600804 | -3.423 | 1.1 | 17.358 | 2.77E-04 | glycine-rich_cell_wall_structural_protein_1-like(LOC102600804) | mRNA |
| 218 | 102581815 | -2.39 | 0.317 | 17.278 | 2.84E-04 | probable_WRKY_transcription_factor_49(LOC102581815) | mRNA |
| 219 | 102590118 | -3.924 | -0.436 | 17.203 | 2.91E-04 | proteinase_inhibitor_type-2_K-like(LOC102590118) | mRNA |
| 220 | 102583955 | -2.095 | 0.078 | 17.096 | 3.00E-04 | putative_cyclin-D6-1(LOC102583955) | mRNA |
| 221 | 102578783 | -2.593 | 3.935 | 16.99 | 3.12E-04 | uncharacterized(LOC102578783) | mRNA |
| 222 | 102605006 | -2.837 | 1.397 | 16.936 | 3.17E-04 | pectinesterase-like(LOC102605006) | mRNA |
| 223 | 102583921 | -2.521 | 0.752 | 16.864 | 3.25E-04 | suberization-associated_anionic_peroxidase_2-like(LOC102583921) | mRNA |
| 224 | 102578245 | -2.589 | 1.971 | 16.85 | 3.26E-04 | universal_stress_protein_A-like_protein(LOC102578245) | mRNA |
| 225 | 102599334 | -2.18 | 1.007 | 16.822 | 3.29E-04 | histone_H3.2-like(LOC102599334) | mRNA |
| 226 | 102591379 | -2.158 | -0.388 | 16.795 | 3.30E-04 | tyramine_N-feruloyltransferase_10/30-like(LOC102591379) | mRNA |
| 227 | 102588449 | -2.296 | 6.998 | 16.715 | 3.41E-04 | heat_shock_cognate_70_kDa_protein_2-like(LOC102588449) | mRNA |
| 228 | 102578392 | -2.291 | -0.286 | 16.633 | 3.48E-04 | probable_membrane-associated_kinase_regulator_2(LOC102578392) | mRNA |
| 229 | 102592148 | -2.57 | 1.626 | 16.562 | 3.58E-04 | uncharacterized(LOC102592148) | mRNA |
| 230 | 102605593 | -2.373 | 1.829 | 16.38 | 3.81E-04 | patatin-2-Kuras_4-like(LOC102605593) | misc_RNA |
| 231 | 107062635 | -2.051 | 1.179 | 16.103 | 4.17E-04 | uncharacterized(LOC107062635) | ncRNA |
| 232 | 102605762 | -3.546 | 5.234 | 15.963 | 4.37E-04 | 17.7_kDa_class_I_heat_shock_protein-like(LOC102605762) | mRNA |
| 233 | 102589748 | -2.275 | 2.27 | 15.555 | 5.01E-04 | probable_N-acetyltransferase_HLS1(LOC102589748) | mRNA |
| 234 | 102596553 | -2.294 | 2.959 | 15.351 | 5.36E-04 | osmotin-like_protein(LOC102596553) | mRNA |
| 235 | 102586498 | -2.651 | 3.597 | 15.319 | 5.42E-04 | acanthoscurrin-1-like(LOC102586498) | mRNA |
| 236 | 102596213 | -2.067 | 0.15 | 15.295 | 5.46E-04 | kinase-interacting_family_protein-like(LOC102596213) | mRNA |
| 237 | 102590433 | -2.547 | 4.228 | 15.293 | 5.47E-04 | uncharacterized(LOC102590433) | mRNA |
| 238 | 102586946 | -2.216 | -0.087 | 15.213 | 5.61E-04 | putative_phospholipid:diacylglycerol_acyltransferase_2(LOC102586946) | mRNA |
| 239 | 102593894 | -2.784 | -0.432 | 15.216 | 5.61E-04 | zingipain-2-like(LOC102593894) | mRNA |
| 240 | 102595219 | -2.011 | 6.481 | 15.161 | 5.72E-04 | heat_shock_cognate_70_kDa_protein-like(LOC102595219) | mRNA |
| 241 | 102580665 | -2.669 | 6.506 | 15.093 | 5.86E-04 | O-acyltransferase_WSD1-like(LOC102580665) | mRNA |
| 242 | 102605134 | -2.467 | 1.976 | 14.966 | 6.12E-04 | gibberellin_2-beta-dioxygenase_1-like(LOC102605134) | mRNA |
| 243 | 102588416 | -2.881 | 4.948 | 14.944 | 6.16E-04 | 17.4_kDa_class_I_heat_shock_protein-like(LOC102588416) | mRNA |
| 244 | 102593998 | -2.297 | 0.548 | 14.813 | 6.44E-04 | B-box_zinc_finger_protein_22-like(LOC102593998) | mRNA |
| 245 | 102596484 | -2.849 | 5.625 | 14.8 | 6.47E-04 | homeobox-leucine_zipper_protein_ATHB-12(LOC102596484) | mRNA |
| 246 | 102581731 | -2.303 | 1.974 | 14.665 | 6.78E-04 | xyloglucan_endotransglucosylase/hydrolase_protein_24-like(LOC102581731) | mRNA |
| 247 | 102581269 | -2.829 | -0.824 | 14.522 | 7.10E-04 | proteinase_inhibitor_type-2(LOC102581269) | mRNA |
| 248 | 102599928 | -2.193 | 6.197 | 14.493 | 7.20E-04 | GDSL_esterase/lipase_At5g33370-like(LOC102599928) | mRNA |
| 249 | 107059433 | -2.016 | -0.069 | 14.477 | 7.21E-04 | uncharacterized(LOC107059433) | ncRNA |
| 250 | 102590558 | -3.203 | -0.68 | 14.485 | 7.22E-04 | GDSL_esterase/lipase_EXL3-like(LOC102590558) | mRNA |
| 251 | 102578143 | -2.497 | -0.64 | 14.404 | 7.39E-04 | uncharacterized(LOC102578143) | mRNA |
| 252 | 102594691 | -2.028 | 1.874 | 14.318 | 7.65E-04 | 3-ketoacyl-CoA_synthase_11-like(LOC102594691) | mRNA |
| 253 | 102588469 | -2.023 | 2.427 | 14.287 | 7.73E-04 | photosystem_II_5_kDa_protein_chloroplastic-like(LOC102588469) | mRNA |
| 254 | 102587692 | -2.601 | 3.104 | 14.278 | 7.76E-04 | uncharacterized(LOC102587692) | mRNA |
| 255 | 102604810 | -2.032 | 3.273 | 14.166 | 8.06E-04 | CO(2)-response_secreted_protease-like(LOC102604810) | mRNA |
| 256 | 102586038 | -2.326 | 1.05 | 14.116 | 8.21E-04 | protein_RAFTIN_1B-like(LOC102586038) | mRNA |
| 257 | 102605602 | -2.603 | -0.216 | 14.08 | 8.31E-04 | 3-oxoacyl-[acyl-carrier-protein]_synthase_I_chloroplastic-like(LOC102605602) | mRNA |
| 258 | 102604529 | -3.039 | 6.176 | 14.003 | 8.54E-04 | 17.3_kDa_class_II_heat_shock_protein-like(LOC102604529) | mRNA |
| 259 | 102596064 | -2.15 | 0.177 | 13.577 | 9.93E-04 | probable_trans-2-enoyl-CoA_reductase_mitochondrial(LOC102596064) | mRNA |

**E. Upregulated genes between ATL07 (Mock) and ATL07(PVY-innoculated)**

| Rank | GeneID | LogFC | LogCPM | F | P_value | Annotation | RNAtype |
| --- | --- | --- | --- | --- | --- | --- | --- |
|  |  |  |  |  |  |  |  |
| 1 | 102602952 | 2.527 | 5.743 | 23.619 | 5.52E-05 | 17.3_kDa_class_II_heat_shock_protein(LOC102602952) | mRNA |
| 2 | 102604529 | 2.419 | 4.761 | 19.123 | 1.94E-04 | 17.3_kDa_class_II_heat_shock_protein-like(LOC102604529) | mRNA |
| 3 | 102588418 | 2.236 | 2.637 | 17.972 | 2.73E-04 | heat_shock_70_kDa_protein_5(LOC102588418) | mRNA |
| 4 | 102578786 | 2.815 | 3.635 | 16.892 | 3.81E-04 | 17.6_kDa_class_I_heat_shock_protein-like(LOC102578786) | mRNA |
| 5 | 102577439 | 3.319 | 2.513 | 14.885 | 7.25E-04 | CBF2(LOC102577439) | mRNA |
| 6 | 102605762 | 2.505 | 2.247 | 14.7 | 7.70E-04 | 17.7_kDa_class_I_heat_shock_protein-like(LOC102605762) | mRNA |
| 7 | 102581787 | 4.184 | -0.291 | 14.53 | 7.95E-04 | uncharacterized(LOC102581787) | mRNA |
| 8 | 102585630 | 2.118 | 2.285 | 14.221 | 9.04E-04 | serpin-ZX-like(LOC102585630) | mRNA |

**F. Downregulated genes between ATL07 (Mock) and ATL07(PVY-innoculated)**

| Rank | GeneID | LogFC | LogCPM | F | P_value | Annotation | RNAtype |
| --- | --- | --- | --- | --- | --- | --- | --- |
|  |  |  |  |  |  |  |  |
| 1 | 102587711 | -5.724 | -0.378 | 64.517 | 2.04E-08 | MADS-box_protein_CMB1(AP1) | mRNA |
| 2 | 102605396 | -2.151 | 0.993 | 25.853 | 2.97E-05 | F-box_protein_CPR30-like(LOC102605396) | mRNA |
