# Supplementary material for: Overexpression of a modified eIF4E regulates potato virus Y resistance at the transcriptional level in potato
Source: BMC Genomics. 2020 Jan 6;21:18. doi: 10.1186/s12864-019-6423-5 (PMC6945410; doi:10.1186/s12864-019-6423-5)
Supplement: Supplementary file 8 — Additional file 8 : Table S6. GO enrichment analysis on gene sets in various treatments [file 12864_2019_6423_MOESM8_ESM.docx]

**Additional table 6: GO enrichment analysis on gene sets in various treatments**

1. **GO term enrichment in ATLWT(Mock) vs ATL07(Mock) treatments**

Biological Process

Rank GO ID Term Annotated Significant Expected P-value

**1 GO:0009408 response to heat 51 11 0.68 4.6e-11**

**2 GO:0009266 response to temperature stimulus 106 11 1.42 1.5e-07**

**3 GO:0006869 lipid transport 36 7 0.48 3.9e-07**

**4 GO:0010876 lipid localization 42 7 0.56 1.2e-06**

**5 GO:0009628 response to abiotic stimulus 370 17 4.96 7.5e-06**

**6 GO:0006950 response to stress 861 27 11.54 1.8e-05**

**7 GO:0000302 response to reactive oxygen species 29 5 0.39 3.6e-05**

**8 GO:1901700 response to oxygen-containing compound 269 13 3.61 5.9e-05**

**9 GO:0042542 response to hydrogen peroxide 10 3 0.13 0.00026**

**10 GO:0050896 response to stimulus 1596 37 21.4 0.00029**

**11 GO:0006979 response to oxidative stress 130 8 1.74 0.00034**

**12 GO:0009750 response to fructose 4 2 0.05 0.00105**

**13 GO:0009644 response to high light intensity 17 3 0.23 0.00139**

**14 GO:0044262 cellular carbohydrate metabolic process 170 8 2.28 0.00196**

**15 GO:0042221 response to chemical 548 16 7.35 0.00249**

**16 GO:0010035 response to inorganic substance 149 7 2 0.00379**

**17 GO:0010411 xyloglucan metabolic process 27 3 0.36 0.00543**

**18 GO:0009749 response to glucose 9 2 0.12 0.00603**

**19 GO:0030001 metal ion transport 92 5 1.23 0.00772**

**20 GO:0009746 response to hexose 11 2 0.15 0.00905**

**21 GO:0034284 response to monosaccharide 11 2 0.15 0.00905**

**22 GO:0009642 response to light intensity 33 3 0.44 0.00955**

**23 GO:0044036 cell wall macromolecule metabolic proces... 63 4 0.84 0.00994**

24 GO:0046839 phospholipid dephosphorylation 12 2 0.16 0.01077

25 GO:0046856 phosphatidylinositol dephosphorylation 12 2 0.16 0.01077

26 GO:0006809 nitric oxide biosynthetic process 1 1 0.01 0.01341

27 GO:0043617 cellular response to sucrose starvation 1 1 0.01 0.01341

28 GO:0045792 negative regulation of cell size 1 1 0.01 0.01341

29 GO:0046209 nitric oxide metabolic process 1 1 0.01 0.01341

30 GO:0048576 positive regulation of short-day photope... 1 1 0.01 0.01341

31 GO:0009416 response to light stimulus 146 6 1.96 0.01351

32 GO:0006073 cellular glucan metabolic process 107 5 1.43 0.01426

33 GO:0044042 glucan metabolic process 108 5 1.45 0.01480

34 GO:0010410 hemicellulose metabolic process 40 3 0.54 0.01617

35 GO:0009690 cytokinin metabolic process 15 2 0.2 0.01669

36 GO:0009314 response to radiation 154 6 2.06 0.01718

37 GO:0006457 protein folding 114 5 1.53 0.01831

38 GO:0009064 glutamine family amino acid metabolic pr... 42 3 0.56 0.01843

39 GO:0009744 response to sucrose 17 2 0.23 0.02125

40 GO:0034285 response to disaccharide 17 2 0.23 0.02125

41 GO:0010383 cell wall polysaccharide metabolic proce... 45 3 0.6 0.02214

42 GO:0006778 porphyrin-containing compound metabolic ... 46 3 0.62 0.02345

43 GO:0034754 cellular hormone metabolic process 18 2 0.24 0.02370

44 GO:0044264 cellular polysaccharide metabolic proces... 123 5 1.65 0.02453

45 GO:0006097 glyoxylate cycle 2 1 0.03 0.02664

46 GO:0009061 anaerobic respiration 2 1 0.03 0.02664

47 GO:0010345 suberin biosynthetic process 2 1 0.03 0.02664

48 GO:0010929 positive regulation of auxin mediated si... 2 1 0.03 0.02664

49 GO:0042126 nitrate metabolic process 2 1 0.03 0.02664

50 GO:0042128 nitrate assimilation 2 1 0.03 0.02664

51 GO:0080027 response to herbivore 2 1 0.03 0.02664

52 GO:1902395 regulation of 1-deoxy-D-xylulose-5-phosp... 2 1 0.03 0.02664

53 GO:1903409 reactive oxygen species biosynthetic pro... 2 1 0.03 0.02664

54 GO:2001057 reactive nitrogen species metabolic proc... 2 1 0.03 0.02664

55 GO:0033013 tetrapyrrole metabolic process 52 3 0.7 0.03224

56 GO:0000304 response to singlet oxygen 3 1 0.04 0.03969

57 GO:0006528 asparagine metabolic process 3 1 0.04 0.03969

58 GO:0006529 asparagine biosynthetic process 3 1 0.04 0.03969

59 GO:0006915 apoptotic process 3 1 0.04 0.03969

60 GO:0010271 regulation of chlorophyll catabolic proc... 3 1 0.04 0.03969

61 GO:0010322 regulation of isopentenyl diphosphate bi... 3 1 0.04 0.03969

62 GO:0017157 regulation of exocytosis 3 1 0.04 0.03969

63 GO:0042981 regulation of apoptotic process 3 1 0.04 0.03969

64 GO:0046487 glyoxylate metabolic process 3 1 0.04 0.03969

65 GO:0048281 inflorescence morphogenesis 3 1 0.04 0.03969

66 GO:0051046 regulation of secretion 3 1 0.04 0.03969

67 GO:0055129 L-proline biosynthetic process 3 1 0.04 0.03969

68 GO:0071071 regulation of phospholipid biosynthetic ... 3 1 0.04 0.03969

69 GO:0071941 nitrogen cycle metabolic process 3 1 0.04 0.03969

70 GO:1901404 regulation of tetrapyrrole catabolic pro... 3 1 0.04 0.03969

71 GO:1903530 regulation of secretion by cell 3 1 0.04 0.03969

72 GO:1903725 regulation of phospholipid metabolic pro... 3 1 0.04 0.03969

73 GO:0010817 regulation of hormone levels 57 3 0.76 0.04070

74 GO:0016567 protein ubiquitination 99 4 1.33 0.04367

75 GO:0009743 response to carbohydrate 25 2 0.34 0.04374

76 GO:0009648 photoperiodism 26 2 0.35 0.04698

77 GO:0048573 photoperiodism, flowering 26 2 0.35 0.04698

78 GO:0005976 polysaccharide metabolic process 198 6 2.65 0.04976

Cellular Compartment

Rank GO ID Term Annotated Significant Expected P-value

**1 GO:0005576 extracellular region 239 10 3.15 0.0012**

**2 GO:0000322 storage vacuole 6 2 0.08 0.0025**

**3 GO:0000326 protein storage vacuole 6 2 0.08 0.0025**

**4 GO:0000325 plant-type vacuole 27 3 0.36 0.0052**

**5 GO:0048046 apoplast 85 5 1.12 0.0052**

6 GO:0031463 Cul3-RING ubiquitin ligase complex 15 2 0.2 0.0162

7 GO:0005773 vacuole 176 6 2.32 0.0289

8 GO:0005782 peroxisomal matrix 3 1 0.04 0.0390

9 GO:0031907 microbody lumen 3 1 0.04 0.0390

10 GO:0005618 cell wall 150 5 1.98 0.0482

11 GO:0030312 external encapsulating structure 150 5 1.98 0.0482

12 GO:0005795 Golgi stack 6 1 0.08 0.0765

13 GO:0016607 nuclear speck 8 1 0.11 0.1007

14 GO:0016021 integral component of membrane 5445 79 71.76 0.1344

15 GO:0071944 cell periphery 703 13 9.26 0.1359

16 GO:0031224 intrinsic component of membrane 5464 79 72.01 0.1435

17 GO:0009705 plant-type vacuole membrane 13 1 0.17 0.1585

18 GO:0016459 myosin complex 13 1 0.17 0.1585

19 GO:0016604 nuclear body 15 1 0.2 0.1805

20 GO:0044438 microbody part 16 1 0.21 0.1914

21 GO:0044439 peroxisomal part 16 1 0.21 0.1914

22 GO:0005887 integral component of plasma membrane 18 1 0.24 0.2126

23 GO:0031461 cullin-RING ubiquitin ligase complex 66 2 0.87 0.2160

24 GO:0044425 membrane part 5698 80 75.09 0.2351

25 GO:0000151 ubiquitin ligase complex 86 2 1.13 0.3136

26 GO:0016020 membrane 6311 86 83.17 0.3515

27 GO:0031226 intrinsic component of plasma membrane 35 1 0.46 0.3719

28 GO:0015629 actin cytoskeleton 38 1 0.5 0.3965

29 GO:0005886 plasma membrane 544 8 7.17 0.4272

30 GO:0009522 photosystem I 48 1 0.63 0.4717

Molecular Function

Rank GO ID Term Annotated Significant Expected P-value

**1 GO:0010333 terpene synthase activity 33 5 0.44 6.8e-05**

**2 GO:0008289 lipid binding 136 9 1.8 8.3e-05**

**3 GO:0016838 carbon-oxygen lyase activity, acting on ... 39 5 0.52 0.00016**

**4 GO:0046527 glucosyltransferase activity 90 6 1.19 0.00125**

**5 GO:0043169 cation binding 2175 44 28.86 0.00222**

**6 GO:0000287 magnesium ion binding 108 6 1.43 0.00315**

**7 GO:0046872 metal ion binding 2164 43 28.72 0.00355**

**8 GO:0016762 xyloglucan:xyloglucosyl transferase acti... 27 3 0.36 0.00532**

9 GO:0004067 asparaginase activity 1 1 0.01 0.01327

10 GO:0004411 homogentisate 1,2-dioxygenase activity 1 1 0.01 0.01327

11 GO:0043546 molybdopterin cofactor binding 1 1 0.01 0.01327

12 GO:0050464 nitrate reductase (NADPH) activity 1 1 0.01 0.01327

13 GO:0080013 (E,E)-geranyllinalool synthase activity 1 1 0.01 0.01327

14 GO:0004722 protein serine/threonine phosphatase act... 72 4 0.96 0.01533

15 GO:0016835 carbon-oxygen lyase activity 119 5 1.58 0.02114

16 GO:0004349 glutamate 5-kinase activity 2 1 0.03 0.02637

17 GO:0004350 glutamate-5-semialdehyde dehydrogenase a... 2 1 0.03 0.02637

18 GO:0004474 malate synthase activity 2 1 0.03 0.02637

19 GO:0008940 nitrate reductase activity 2 1 0.03 0.02637

20 GO:0015140 malate transmembrane transporter activit... 2 1 0.03 0.02637

21 GO:0015204 urea transmembrane transporter activity 2 1 0.03 0.02637

22 GO:0016713 oxidoreductase activity, acting on paire... 2 1 0.03 0.02637

23 GO:0018685 alkane 1-monooxygenase activity 2 1 0.03 0.02637

24 GO:0046857 oxidoreductase activity, acting on other... 2 1 0.03 0.02637

25 GO:0046910 pectinesterase inhibitor activity 2 1 0.03 0.02637

26 GO:0004857 enzyme inhibitor activity 133 5 1.76 0.03215

27 GO:0005506 iron ion binding 335 9 4.45 0.03483

28 GO:0016491 oxidoreductase activity 1355 26 17.98 0.03496

29 GO:0004066 asparagine synthase (glutamine-hydrolyzi... 3 1 0.04 0.03929

30 GO:0004109 coproporphyrinogen oxidase activity 3 1 0.04 0.03929

31 GO:0015556 C4-dicarboxylate transmembrane transport... 3 1 0.04 0.03929

32 GO:0016427 tRNA (cytosine) methyltransferase activi... 3 1 0.04 0.03929

33 GO:0016428 tRNA (cytosine-5-)-methyltransferase act... 3 1 0.04 0.03929

34 GO:0019905 syntaxin binding 3 1 0.04 0.03929

35 GO:0004721 phosphoprotein phosphatase activity 145 5 1.92 0.04400

36 GO:0046906 tetrapyrrole binding 406 10 5.39 0.04443

37 GO:0035251 UDP-glucosyltransferase activity 62 3 0.82 0.04918

1. **GO term enrichment in ATLWT (Mock) vs ATLWT (PVY) treatments**

Biological Process

Rank GO ID Term Annotated Significant Expected P-value

**1 GO:0009408 response to heat 51 12 1.02 2.2e-10**

**2 GO:0009266 response to temperature stimulus 106 13 2.12 1.6e-07**

**3 GO:0010876 lipid localization 42 8 0.84 1.4e-06**

**4 GO:0006950 response to stress 861 38 17.21 1.7e-06**

**5 GO:0006869 lipid transport 36 7 0.72 5.8e-06**

**6 GO:0006979 response to oxidative stress 130 12 2.6 1.0e-05**

**7 GO:0010411 xyloglucan metabolic process 27 6 0.54 1.2e-05**

**8 GO:0000302 response to reactive oxygen species 29 6 0.58 1.9e-05**

**9 GO:0042542 response to hydrogen peroxide 10 4 0.2 2.9e-05**

**10 GO:1901700 response to oxygen-containing compound 269 16 5.38 9.0e-05**

**11 GO:0010410 hemicellulose metabolic process 40 6 0.8 0.00013**

**12 GO:0010383 cell wall polysaccharide metabolic proce... 45 6 0.9 0.00025**

**13 GO:0009644 response to high light intensity 17 4 0.34 0.00030**

**14 GO:0071555 cell wall organization 170 11 3.4 0.00058**

**15 GO:0045229 external encapsulating structure organiz... 174 11 3.48 0.00071**

**16 GO:0009628 response to abiotic stimulus 370 17 7.4 0.00112**

**17 GO:0006073 cellular glucan metabolic process 107 8 2.14 0.00131**

**18 GO:0044042 glucan metabolic process 108 8 2.16 0.00139**

**19 GO:0044036 cell wall macromolecule metabolic proces... 63 6 1.26 0.00154**

**20 GO:0042546 cell wall biogenesis 65 6 1.3 0.00182**

**21 GO:0044262 cellular carbohydrate metabolic process 170 10 3.4 0.00211**

**22 GO:0009750 response to fructose 4 2 0.08 0.00232**

**23 GO:0010035 response to inorganic substance 149 9 2.98 0.00293**

**24 GO:0044264 cellular polysaccharide metabolic proces... 123 8 2.46 0.00316**

**25 GO:0009642 response to light intensity 33 4 0.66 0.00401**

**26 GO:0005976 polysaccharide metabolic process 198 10 3.96 0.00627**

**27 GO:0071554 cell wall organization or biogenesis 235 11 4.7 0.00738**

**28 GO:0050896 response to stimulus 1596 45 31.9 0.00768**

**29 GO:0006560 proline metabolic process 7 2 0.14 0.00781**

**30 GO:0009064 glutamine family amino acid metabolic pr... 42 4 0.84 0.00954**

31 GO:0030001 metal ion transport 92 6 1.84 0.01009

32 GO:0042221 response to chemical 548 19 10.95 0.01294

33 GO:0009749 response to glucose 9 2 0.18 0.01304

34 GO:0009746 response to hexose 11 2 0.22 0.01941

35 GO:0034284 response to monosaccharide 11 2 0.22 0.01941

36 GO:0006809 nitric oxide biosynthetic process 1 1 0.02 0.01999

37 GO:0010344 seed oilbody biogenesis 1 1 0.02 0.01999

38 GO:0030417 nicotianamine metabolic process 1 1 0.02 0.01999

39 GO:0030418 nicotianamine biosynthetic process 1 1 0.02 0.01999

40 GO:0043617 cellular response to sucrose starvation 1 1 0.02 0.01999

41 GO:0046209 nitric oxide metabolic process 1 1 0.02 0.01999

42 GO:0072351 tricarboxylic acid biosynthetic process 1 1 0.02 0.01999

43 GO:0001101 response to acid chemical 204 9 4.08 0.02101

44 GO:0046839 phospholipid dephosphorylation 12 2 0.24 0.02298

45 GO:0046856 phosphatidylinositol dephosphorylation 12 2 0.24 0.02298

46 GO:0042545 cell wall modification 36 3 0.72 0.03459

47 GO:0009753 response to jasmonic acid 37 3 0.74 0.03711

48 GO:0043436 oxoacid metabolic process 533 17 10.65 0.03722

49 GO:0006082 organic acid metabolic process 535 17 10.69 0.03836

50 GO:0009061 anaerobic respiration 2 1 0.04 0.03958

51 GO:0010929 positive regulation of auxin mediated si... 2 1 0.04 0.03958

52 GO:0032780 negative regulation of ATPase activity 2 1 0.04 0.03958

53 GO:0042126 nitrate metabolic process 2 1 0.04 0.03958

54 GO:0042128 nitrate assimilation 2 1 0.04 0.03958

55 GO:0043462 regulation of ATPase activity 2 1 0.04 0.03958

56 GO:0080027 response to herbivore 2 1 0.04 0.03958

57 GO:1903409 reactive oxygen species biosynthetic pro... 2 1 0.04 0.03958

58 GO:2001057 reactive nitrogen species metabolic proc... 2 1 0.04 0.03958

59 GO:0009611 response to wounding 38 3 0.76 0.03971

60 GO:0009744 response to sucrose 17 2 0.34 0.04438

61 GO:0034285 response to disaccharide 17 2 0.34 0.04438

62 GO:0006629 lipid metabolic process 512 16 10.24 0.04973

Cellular Component

Rank GO ID Term Annotated Significant Expected P-value

**1 GO:0005576 extracellular region 239 22 4.63 1.3e-09**

**2 GO:0048046 apoplast 85 11 1.65 6.8e-07**

**3 GO:0005618 cell wall 150 11 2.91 0.00016**

**4 GO:0030312 external encapsulating structure 150 11 2.91 0.00016**

**5 GO:0012511 monolayer-surrounded lipid storage body 4 2 0.08 0.00219**

**6 GO:0000322 storage vacuole 6 2 0.12 0.00533**

**7 GO:0000326 protein storage vacuole 6 2 0.12 0.00533**

8 GO:0005811 lipid droplet 10 2 0.19 0.01518

Molecular Function

Rank GO ID Term Annotated Significant Expected P-value

**1 GO:0003700 DNA binding transcription factor activit... 393 27 7.79 1.9e-08**

**2 GO:0140110 transcription regulator activity 468 28 9.28 1.9e-07**

**3 GO:0016762 xyloglucan:xyloglucosyl transferase acti... 27 6 0.54 1.2e-05**

**4 GO:0005509 calcium ion binding 205 14 4.07 6.0e-05**

**5 GO:0046527 glucosyltransferase activity 90 9 1.78 7.2e-05**

**6 GO:0030170 pyridoxal phosphate binding 98 9 1.94 0.00014**

**7 GO:0070279 vitamin B6 binding 98 9 1.94 0.00014**

**8 GO:0019842 vitamin binding 122 9 2.42 0.00072**

**9 GO:0016831 carboxy-lyase activity 59 6 1.17 0.00108**

**10 GO:0004084 branched-chain-amino-acid transaminase a... 4 2 0.08 0.00229**

**11 GO:0052654 L-leucine transaminase activity 4 2 0.08 0.00229**

**12 GO:0052655 L-valine transaminase activity 4 2 0.08 0.00229**

**13 GO:0052656 L-isoleucine transaminase activity 4 2 0.08 0.00229**

**14 GO:0008289 lipid binding 136 8 2.7 0.00573**

**15 GO:0016830 carbon-carbon lyase activity 82 6 1.63 0.00573**

**16 GO:0005516 calmodulin binding 40 4 0.79 0.00789**

17 GO:0045735 nutrient reservoir activity 23 3 0.46 0.01019

18 GO:0003677 DNA binding 1294 37 25.66 0.01476

19 GO:0003720 telomerase activity 1 1 0.02 0.01983

20 GO:0003721 telomerase RNA reverse transcriptase act... 1 1 0.02 0.01983

21 GO:0004837 tyrosine decarboxylase activity 1 1 0.02 0.01983

22 GO:0030410 nicotianamine synthase activity 1 1 0.02 0.01983

23 GO:0043546 molybdopterin cofactor binding 1 1 0.02 0.01983

24 GO:0050464 nitrate reductase (NADPH) activity 1 1 0.02 0.01983

25 GO:0080013 (E,E)-geranyllinalool synthase activity 1 1 0.02 0.01983

26 GO:0010333 terpene synthase activity 33 3 0.65 0.02716

27 GO:0008483 transaminase activity 37 3 0.73 0.03652

28 GO:0016769 transferase activity, transferring nitro... 37 3 0.73 0.03652

29 GO:0004349 glutamate 5-kinase activity 2 1 0.04 0.03927

30 GO:0004350 glutamate-5-semialdehyde dehydrogenase a... 2 1 0.04 0.03927

31 GO:0004512 inositol-3-phosphate synthase activity 2 1 0.04 0.03927

32 GO:0004657 proline dehydrogenase activity 2 1 0.04 0.03927

33 GO:0008940 nitrate reductase activity 2 1 0.04 0.03927

34 GO:0042030 ATPase inhibitor activity 2 1 0.04 0.03927

35 GO:0046857 oxidoreductase activity, acting on other... 2 1 0.04 0.03927

36 GO:0046910 pectinesterase inhibitor activity 2 1 0.04 0.03927

37 GO:0016838 carbon-oxygen lyase activity, acting on ... 39 3 0.77 0.04174

38 GO:0016758 transferase activity, transferring hexos... 391 13 7.75 0.04836

39 GO:0004867 serine-type endopeptidase inhibitor acti... 18 2 0.36 0.04866

**C: GO term enrichment in ATL07(Mock) vs ATL07(PVY) treatments**

Biological Process

Rank GO ID Term Annotated Significant Expected P-value

1 GO:0045944 positive regulation of transcription fro... 42 1 0.01 0.010

2 GO:0006351 transcription, DNA-templated 1025 2 0.25 0.016

3 GO:0097659 nucleic acid-templated transcription 1035 2 0.25 0.016

4 GO:0032774 RNA biosynthetic process 1036 2 0.25 0.016

5 GO:0006357 regulation of transcription from RNA pol... 71 1 0.02 0.017

6 GO:0034654 nucleobase-containing compound biosynthe... 1159 2 0.28 0.020

7 GO:0045893 positive regulation of transcription, DN... 85 1 0.02 0.021

8 GO:1902680 positive regulation of RNA biosynthetic ... 85 1 0.02 0.021

9 GO:1903508 positive regulation of nucleic acid-temp... 85 1 0.02 0.021

10 GO:0051254 positive regulation of RNA metabolic pro... 87 1 0.02 0.021

11 GO:0045935 positive regulation of nucleobase-contai... 95 1 0.02 0.023

12 GO:0010557 positive regulation of macromolecule bio... 97 1 0.02 0.024

13 GO:0018130 heterocycle biosynthetic process 1270 2 0.31 0.024

14 GO:0019438 aromatic compound biosynthetic process 1273 2 0.31 0.024

15 GO:0031328 positive regulation of cellular biosynth... 100 1 0.02 0.024

16 GO:0006366 transcription from RNA polymerase II pro... 102 1 0.02 0.025

17 GO:0009891 positive regulation of biosynthetic proc... 102 1 0.02 0.025

18 GO:0010628 positive regulation of gene expression 105 1 0.03 0.025

19 GO:1901362 organic cyclic compound biosynthetic pro... 1341 2 0.33 0.027

20 GO:0051173 positive regulation of nitrogen compound... 134 1 0.03 0.032

21 GO:0010604 positive regulation of macromolecule met... 140 1 0.03 0.034

22 GO:0031325 positive regulation of cellular metaboli... 140 1 0.03 0.034

23 GO:0009893 positive regulation of metabolic process 152 1 0.04 0.037

24 GO:0044271 cellular nitrogen compound biosynthetic ... 1687 2 0.41 0.042

25 GO:0034645 cellular macromolecule biosynthetic proc... 1704 2 0.42 0.043

26 GO:0009059 macromolecule biosynthetic process 1739 2 0.42 0.045

27 GO:0048522 positive regulation of cellular process 195 1 0.05 0.047

Cellular Compartment

Rank GO ID Term Annotated Significant Expected P-value

1 GO:0005615 extracellular space 18 1 0.01 0.011

2 GO:0044421 extracellular region part 21 1 0.01 0.013

Molecular Function

Rank GO ID Term Annotated Significant Expected P-value

1 GO:0003700 DNA binding transcription factor activit... 393 2 0.11 0.0047

2 GO:0140110 transcription regulator activity 468 2 0.14 0.0067

3 GO:0000977 RNA polymerase II regulatory region sequ... 26 1 0.01 0.0076

4 GO:0001012 RNA polymerase II regulatory region DNA ... 26 1 0.01 0.0076

5 GO:0000976 transcription regulatory region sequence... 35 1 0.01 0.0102

6 GO:1990837 sequence-specific double-stranded DNA bi... 40 1 0.01 0.0116

7 GO:0000975 regulatory region DNA binding 52 1 0.02 0.0151

8 GO:0001067 regulatory region nucleic acid binding 52 1 0.02 0.0151

9 GO:0044212 transcription regulatory region DNA bind... 52 1 0.02 0.0151

10 GO:0003690 double-stranded DNA binding 109 1 0.03 0.0314

11 GO:0003677 DNA binding 1294 2 0.38 0.0469
